# The Five-Decade Journey of Small Cell Lung Cancer

**DOI:** 10.34133/cancomm.0052

**Published:** 2026-10-06

**Authors:** Runzhi Chen, Chang Xue, Yi Zhong, Qing Huang, Qianyu Fan, Yuanxiang Li, Jingyi Wu, Shuang Dong, Xiaoyu Li, Tianlin Xiao, Sheng Hu

**Affiliations:** ^1^ Department of Medical Oncology, Tongji Medical College, Hubei Cancer Hospital, Huazhong University of Science and Technology, Wuhan, Hubei, P. R. China.; ^2^ Xiangyang Central Hospital, Affiliated Hospital of Hubei University of Arts and Science, Xiangyang, Hubei, P. R. China.

## Abstract

Over the past 5 decades, small cell lung cancer (SCLC) has persisted as one of the most formidable malignancies, largely attributed to its aggressive early metastasis, limited therapeutic arsenal in both first-line and subsequent treatments, and rapid onset of treatment resistance. The dawn of a new era in the treatment of SCLC appeared in 2018, with the introduction of first-line chemotherapy combined with immune checkpoint inhibitors, as well as tarlatamab for later-line treatment. However, the improvements in survival, although tangible, were still unsatisfactory. Therefore, this review focuses on unraveling the mechanisms underlying cancer initiation, progression, and particularly metastasis, as well as emerging advances in molecular subtyping and drug resistance from the epigenetic perspective. Simultaneously, we outline their hallmark characteristics—high plasticity, heterogeneity, redundancy, and ambiguity driven by intercellular crosstalk under spatiotemporal dependency. Furthermore, we highlight the evolving progress of various successful drugs or therapeutic strategies and molecular biomarkers in clinical practice for SCLC, as well as promising agents under investigation. Finally, we also discuss how to leverage artificial intelligence and innovative biomedical technologies, as well as learn from various challenges in order to improve the therapeutic landscape of SCLC.

## Introduction

Small cell lung cancer (SCLC) first emerged as a diagnostic term in the 1940s and coexisted for years with alternative nomenclatures, such as oat-cell carcinoma, small round cell cancer, or small cell anaplastic lung cancer. It was only formally standardized with the publication of the third edition of the World Health Organization (WHO) classification of lung tumors at the end of the 20th century (Fig. 1). Over the past 5 decades, SCLC has remained one of the most infamous and recalcitrant malignancies, characterized by its widespread early metastasis driven by high levels of circulating tumor cells (CTCs), rapid development of resistance to chemotherapy or chemoimmunotherapy, and a striking paucity of effective later-line treatment options [1,2].

**Fig. 1.**
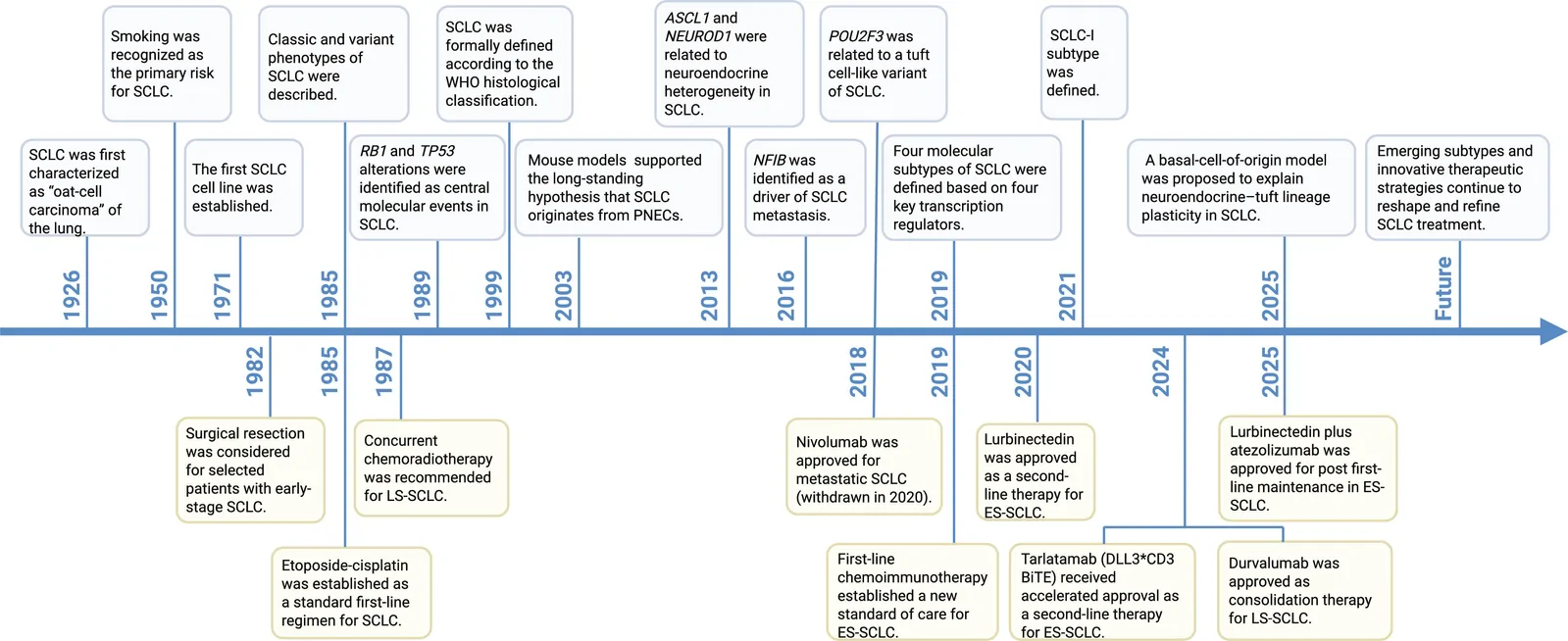
The timeline of milestone events in SCLC. The horizontal arrow separates fundamental research milestones above from significant clinical treatment advancements below, clearly indicating a more pronounced progress in the past decade. This figure was created using BioRender. *ASCL1*, achaete-scute homolog 1; BiTEs, bispecific T cell engagers; DLL3, delta-like ligand 3; CD3, cluster of differentiation 3; ES-SCLC, extensive-stage small cell lung cancer; SCLC-I, small cell lung cancer-inflamed subtype; LS-SCLC, limited-stage small cell lung cancer; *NEUROD1*, neurogenic differentiation factor 1; *NFIB*, nuclear factor I B; PNECs, pulmonary neuroendocrine cells; *POU2F3*, pou class 2 homeobox 3; *RB1*, retinoblastoma 1; *TP53*, tumor protein 53; WHO, World Health Organization.

Although marked progress has been made in basic research and clinical trials, drug options for SCLC have seen few major breakthroughs beyond standard platinum-based chemotherapy. This therapeutic landscape began to change in 2018 with the approval of first-line immune checkpoint inhibitors (ICIs) in combination with platinum-based chemotherapy [3], which established a new standard of care for patients with extensive-stage SCLC and significantly extended overall survival (OS)—from a maximum of 11 months [4] to the current range of 13 to 19 months. However, there are still significant challenges in treating SCLC, such as the insufficiency of benefits from treatment—despite the 3-year survival rate for patients with extensive-stage disease increasing from 9% to 17.6% [3], while the 5-year OS rate remains around 12% [4], and the extremely limited arsenal of available therapeutics, particularly in the field of targeted drugs. Moreover, data obtained from clinical trials are from carefully selected patients, which are inevitably subjected to some degree of real-world applicability discount when extrapolated to the general population, especially for high-risk patients [5], even though the exact magnitude remains unclear.

The SCLC population accounts for 13% to 15% of all lung cancer cases [6]. In view of this, we propose this review to provide an in-depth exploration of the mechanisms underlying cancer initiation, progression, metastasis, and drug resistance of SCLC, and critically synthesize recent advances in molecular classification and biomarker development—with a dedicated emphasis on epigenetic underpinnings. Additionally, we highlight the unique features of biological systems—high plasticity, heterogeneity, redundancy, and ambiguity—which emerge from intercellular or systemic crosstalk under spatiotemporal dependency. We also reveal various successful drugs and treatment strategies in clinical research, as well as those currently under investigation. Importantly, we examine the challenges encountered, extract valuable lessons from the failures across multiple studies, and highlight the need for future research to be spurred by the large-scale integration of innovative biomedicine technologies empowered by artificial intelligence (AI).

## Epidemiology of SCLC

The number of SCLC patients is substantial, with approximately 250,000 new cases being diagnosed each year globally [7]. Overall, the incidence of SCLC has gone through the 3 stages of rapid increase, gradual decrease, and stabilization over the past 50 years; however, this is based on reported data that lacked comprehensiveness, as most studies were based on data from the United States. The continuity of the data was also limited, as there were even fewer annual updates available than those for non-small cell lung cancer (NSCLC). Specifically, the incidence rate increased from 9.5 per 100,000 in 1975 to a peak of 15.3 per 100,000 in 1988, then declined to a low of 6.5 per 100,000 in 2019, and has remained stable since then. The proportion of SCLC among all lung cancer cases has followed a similar trend, increasing from 13.3% in 1975 to a peak of 17.5% in 1986, and then gradually declining to 11.1% in 2019 [8], although minor differences do exist across the different populations [9]. Some data from China suggest that the incidence of SCLC gradually increased to 13.6% between 2008 and 2017 [10]; however, whether the incidence also experienced fluctuations remains unclear, as there was a lack of data prior to 2008 [11].

The fluctuations in incidence over time may be related to variations in the prevalence of tobacco. With the strengthening of tobacco control in China, it is estimated that the epidemiological features of SCLC will become similar to those in the United States [11]. There are clear regional and population differences in SCLC cases. The male high-incidence area is Izmir, Turkey, with an incidence rate of 12.4 per 100,000, while for females, the high-incidence group is Māori individuals in New Zealand, with an incidence rate of 14.5 per 100,000 [12]. Similarly, individuals of African descent may have a lower risk of SCLC compared to those of European descent, and they tend to have a higher 5-year survival rate. This suggests that genetic background based on ancestry—more likely characterized by retinoblastoma 1 (*RB1*) and tumor protein p53 (*TP53*) wild-type status, along with serine/threonine kinase 11 (*STK11*) mutations [13]—may compensate for the disadvantages from access to cancer treatment [14]. Additionally, individuals from low socioeconomic status groups show a higher incidence of SCLC, which aligns with patterns of tobacco consumption, recently endorsed by data from the UK population [15].

Data from Surveillance, Epidemiology, and End Results (SEER) 8 indicated that, between 1975 and 1979, males accounted for the majority (68.6%) of SCLC cases, which gradually declined to 50.2% in 2010–2014, and then reversed, with females accounting for 51.2% of cases during 2014–2019. However, in East Asian countries such as China and Japan, males still constituted the vast majority of cases, similar to the sex distribution observed in squamous cell carcinoma. Like other lung cancer subtypes, such as adenocarcinoma and squamous cell carcinoma, the median age at diagnosis for both males and females has gradually increased, reaching 69 years. Nevertheless, some data from China have suggested that peak incidence occurs around age 50, with the risk decreasing with increasing age [10].

In terms of molecular epidemiology, more than 90% of SCLC patients exhibit biallelic inactivation of the tumor suppressor genes *RB1* and *TP53* [16], which is strongly associated with younger age and smoking history, but less clearly linked to clinical stage, treatment response, or prognosis. Some SCLC cases also show inactivation of functional homologs of *TP53* and *RB1* such as tumor protein p63/73 (*TP63/TP73)* and retinoblastoma-like protein 1/2 (*RBL1/2)*, as well as mutations or amplifications in the NOTCH pathway and oncogenes such as *MYC* and the B cell lymphoma 2 (*BCL2*) gene family, along with differential expressions of key transcriptional regulators. Although these molecular alterations may vary among individuals of different genetic backgrounds, for instance, in younger individuals or non-white populations, wild-type *RB1* and/or *TP53* SCLC are more commonly observed, even though overall tumor mutation burden (TMB) levels remain comparable [13]. Nevertheless, the genetic susceptibility to SCLC remains poorly understood, despite germline pathogenic variants in radiation-sensitive protein 51 paralog D (*RAD51D*), checkpoint kinase 1 (*CHEK1*), breast cancer susceptibility gene 2 (*BRCA2*), and mutY homolog (*MUTYH*) having been reported [17].

Due to the lack of a single dominant pathogenic cause in SCLC, creating highly targeted preventive strategies is still a significant challenge, and smoking cessation and low-dose computed tomography (CT) screening are currently considered the most effective approaches. Although the implementation of lung cancer screening since 2013 has resulted in a higher proportion of early-stage lung cancer diagnoses, whether this screening improves survival outcomes for SCLC patients remains unclear, as most cases are still diagnosed at an advanced stage [8]. At present, early detection through the analysis of CTCs or circulating tumor DNA (ctDNA), including DNA mutations [18], methylation, and fragmentome profiles [19], as well as microRNA (miRNA) and proteomic profiling, has shown promising results.

## Preclinical Research Advances in SCLC

### Origins of SCLC

SCLC most commonly originates from pulmonary neuroendocrine cells (PNECs), which exhibit both neuronal and endocrine characteristics, accounting for approximately 0.41% of the airway epithelium. In some cases, SCLC may also arise from basal cells [20], club cells [although SCLC has not been shown to develop from Clara cell 10-kDa protein (CC10) positive goblet secretory cells], alveolar type 2 (AT2) cells [21], and tuft cells [22]. Notably, the distinct cellular origins of SCLC may determine or shape different evolutionary trajectories or fates [20], leading to significant heterogeneity. Additionally, SCLC may result from the transformation of lung adenocarcinoma following the inhibition of such driver genes as epidermal growth factor receptor (EGFR) (Fig. 2).

**Fig. 2.**
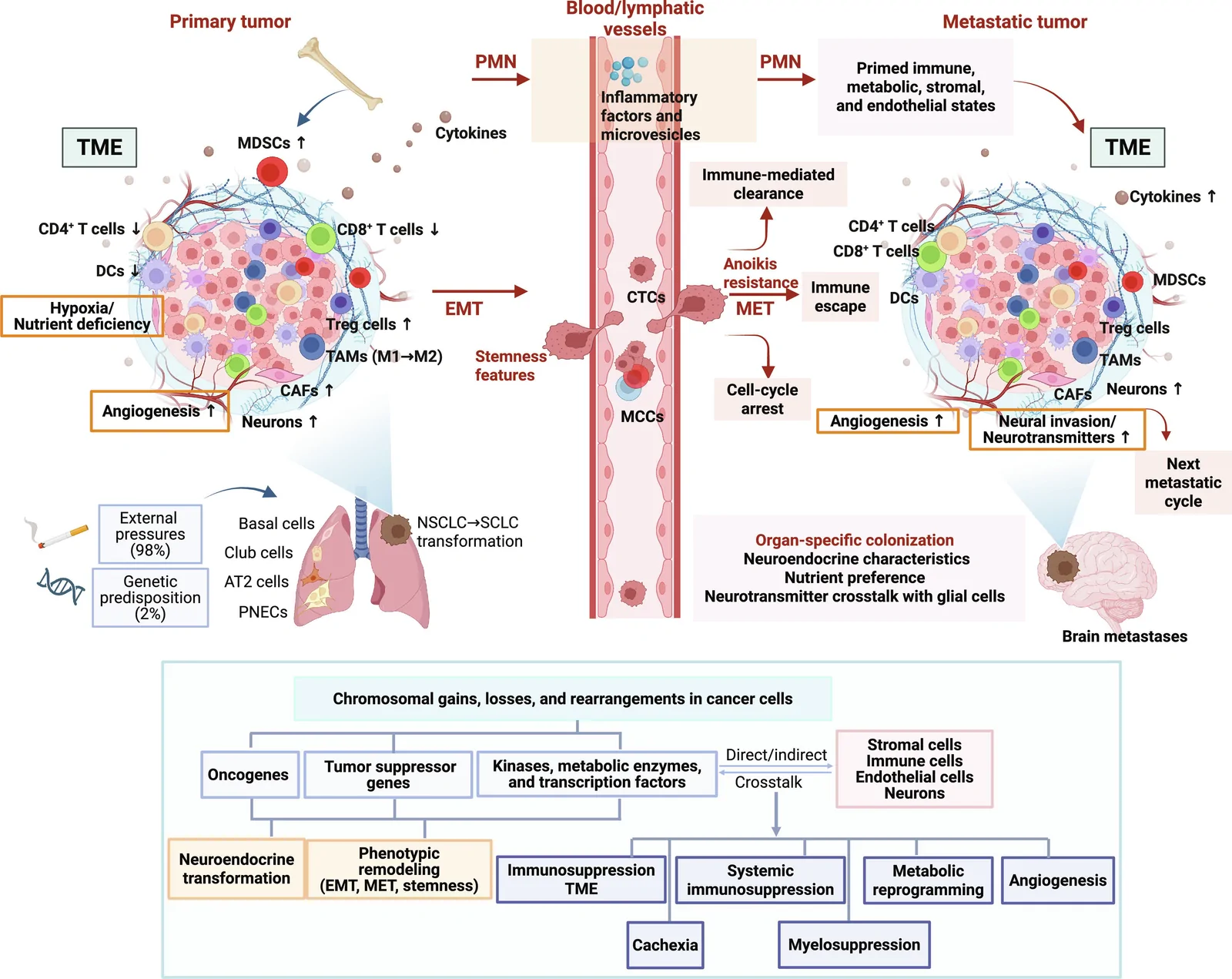
Comprehensive regulatory mechanisms of SCLC metastasis. Although the metastasis of SCLC involves multiple cell types and the synchronized regulation across various signaling pathways, there may be varying degrees of differences in terms of temporal dynamics and the involved components among different patients or tumor sites. SCLC tends to prefer brain parenchymal metastasis over leptomeningeal metastasis, a pattern that is frequently observed in NSCLC following TKI therapy. The mechanisms depicted in the box reflect a general conceptual framework and are not intended to imply association with any specific biological process. The term “immune elimination” denotes the elimination of disseminated cancer cells by the host’s immune system. This figure was created using BioRender. AT2, alveolar type II; CAFs, cancer-associated fibroblasts; CTCs, circulating tumor cells; DCs, dendritic cells; EMT, epithelial–mesenchymal transition; MDSCs, myeloid-derived suppressor cells; MCCs, metastatic cancer cells; NSCLC, non-small cell lung cancer; PNECs, pulmonary neuroendocrine cells; PMN, premetastatic niche; TME, tumor microenvironment; TAMs, tumor-associated macrophages; Treg, regulatory T cell; TKI, tyrosine kinase inhibitor.

### Formation of cancer cells

SCLC’s development is mostly driven by various external carcinogenic pressures (98%), such as toxins from tobacco or e-cigarettes and other combustible materials, which result in acquired genetic and epigenetic alterations. Chromosomal losses are frequently observed in SCLC, with 22 chromosomes being affected, the most common of which are 3p (77.6%), 17p (64.5%), 5q (52.3%), 16q (51.7%), and 10q (51.6%). Chromosomal gains are also frequently observed, particularly on 5p (64.0%), 3q (55.0%), 18q (45.0%), 18p (43.8%), and 8q (41.8%) [13].

Amplifications, deletions, mutations, and rearrangements in the genome involve tumor suppressor genes, such as *TP53* (91.6%), *RB1* (73.5%), phosphatase and tensin homolog (*PTEN*) (9.9%), cyclic adenosine monophosphate response element-binding protein (*CREBBP*) (6.1%), E1A binding protein p300 (*EP300*) (1.6%), *TP73* (13%), *RBL1/2* (6%), cyclin-dependent kinase inhibitor 2A (*CDKN2A*) (4.1%), and *CDKN2B* (1.8%) [23], as well as oncogenes or other cancer-related genes, such as lysine methyltransferase 2D (*KMT2D*) (12.9%), *NOTCH* (10.9%), *MYCL* (7.2%), *MYC* (6.0%), phosphatidylinositol-4,5-bisphosphate 3-kinase catalytic subunit α (*PIK3CA*) (5.6%), rapamycin-insensitive companion of mechanistic target of rapamycin (mTOR) (*RICTOR*) (5.6%), cyclin E1 (*CCNE1*) (4.3%) [13], fibroblast growth factor 10 (*FGF10*) (4.3%), fibroblast growth factor receptor 1 (*FGFR1*) (3.9%), AT-rich interactive domain 1A (*ARID1A*) (3.5%) [24], and other rare mutations, but the frequency of these aberrances is dependent on the study, sample type, and disease stage. While the biological significance of some mutations in SCLC remains unclear, they collectively support the notion that SCLC is largely driven by the loss of tumor suppressor genes and is characterized by a high TMB.

Research into SCLC began with the establishment of the first human cell line in 1971 [25]. Subsequent emerging technologies, such as in vitro culture in 3-dimensional (3D) environments or multicellular organoids [26], and particularly patient-derived xenografts (PDXs) from human tissues, not only allow for a high-resolution and comprehensive match to the genetics, epigenetics, and even the landscape of the tumor microenvironment (TME) but also facilitate tracking of dynamic changes in drug sensitivity. In recent years, the increasing availability of CTCs for SCLC patients [27] has enabled direct longitudinal analysis of CTCs or the construction of circulating tumor cell-derived xenograft (CTC-DX) models, which could provide deeper insights into the mechanisms underlying SCLC’s progression, metastasis, and treatment resistance. Furthermore, immune-competent models incorporating T cell chimerism have increasingly been applied in SCLC research.

In terms of mechanism, Meuwissen et al. [28] utilized a Cre/LoxP-regulated recombination system to inactivate *TP53* (exons 2 to 10) and *RB1* (exon 19) in PNECs and AT2 cells, resulting in the formation of small cell neuroendocrine tumors that closely resembled human SCLC, thereby confirming the cellular origins of SCLC. Later, in an Rbl1^−/−^ mouse background, the deletion of *Rb1*, *PTEN*, and transformation related protein 53 (*Trp53*) genes induced the transformation of basal cells into SCLC exhibiting neuroendocrine (NE) features, marked predominantly by achaete-scute homolog 1 (*ASCL1*) expression [29]. Loss of tumor suppressor genes such as *Rbl2/p130* [30], *CREBBP* [31] and liver kinase B1 (*LKB1*), and downstream effectors of the phosphatidylinositol 3-kinase (PI3K)–AKT–mTOR pathway, such as tuberous sclerosis complex 1 (*TSC1*) [32], also contribute to the progression of SCLC. Recent data suggested that although the inactivation of tumor suppressors could occur in a continuous pattern, *TP53* and *RB1* in SCLC could follow a classical “two-hit” model. Notably, approximately 5.5% of SCLC tumors retain wild-type *TP53* and *RB1*, and a large number of oncogenes—mainly *MYC* and *NOTCH* genes—as well as splice variants of focal adhesion kinase (*FAK*) [33] also participate in malignant transformation.

*MYC*, including *MYC/c-MYC*, *MYC-N*, and *MYC-L*, involved in the regulation of target gene transcription, was first recognized as an oncogene over 40 years ago [34]. In SCLC, *MYC* function becomes dysregulated due to gene translocations or amplifications (with c-*MYC* mutation in 9%, *MYC-L* in 6%, and *MYC-N* in 4%), or through persistent activation of *NOTCH*, *Wnt/β-catenin*, *Ras-PI3K-AKT-GSK3*, and *Ras-Raf-ERK1*. Notably, *MYC* orchestrates persistent protein synthesis, angiogenesis, and immune suppression [35] through sustained signaling activity rather than elevated protein levels. *MYC-L* expression is predominant and high in NE cells, whereas in non-NE cells, *MYC* tends to be more highly expressed [36]. Amplifications of *MYC* family genes frequently occur in extrachromosomal DNA [37], correlating with heightened tumor aggressiveness and cross-resistance to different chemotherapy regimens [38]. In addition to driving the emergence of POU class 2 homeobox 3 (*POU2F3*)^+^ tumors, *MYC* facilitates transitions from *ASCL1*^+^ to neurogenic differentiation factor 1 *(NEUROD1*)^+^ and subsequently to yes-associated protein 1 (*YAP1*)^+^ states—a phenomenon known as non-NE plasticity, which may also be linked to the TME features with T cell infiltration.

The NOTCH receptors, along with their ligands Jagged1/2 (JAG1/2) and delta-like (DLL) 1, 3, and 4 [39], are transmembrane proteins primarily composed of epidermal growth factor (EGF)-like repeats. Upon ligand binding, the NOTCH receptor is cleaved by γ-secretase, releasing the Notch intracellular domain (NICD), which translocates to the nucleus to regulate transcription [40], and is involved in epithelial–mesenchymal transition (EMT), cancer stem cell (CSC) features, angiogenesis, metabolic reprogramming, and the modulation of immune and stromal cell functions, thereby remodeling the TME and serving as a key driver of tumor heterogeneity. Notably, the regulation of the NOTCH signaling pathway depends not only on intercellular signaling and specific transcriptional contexts but also on distinct temporal and spatial dynamics, enabling diverse levels and patterns of signal transmission. In SCLC, NOTCH signaling, in collaboration with the transcriptional co-regulators HES1 and HEY, induces the transcription of target genes and can also promote the transcriptional repression of the RE1-silencing transcription factor (*REST*) through *Yap1* [41], thereby suppressing *ASCL1* and *NEUROD1* expression and contributing to an irreversible fate switch from NE to non-NE cells in a portion of SCLC cases (10% to 50%) [42]. NOTCH signaling exhibits negative feedback regulation with its ligand DLL, as evidenced by the low NOTCH activity commonly associated with suppression of lysine-specific demethylase 5A/retinoblastoma-binding protein 2 (KDM5A/RBP2) [43] and high expression of DLL3, which are observed in most NE-SCLC (approximately 83%) [16]. The NOTCH receptor and NICD undergo extensive posttranslational modifications, including glycosylation, phosphorylation, and precise regulation of ubiquitination and degradation. Furthermore, NOTCH signaling engages in crosstalk with other molecular pathways, such as *Hedgehog*, *Ras*, *HIF1*, *WNT*, *TGF-β*, *MAPK*, and *EGFR*. However, NOTCH signaling can also directly induce significant G1 phase cell cycle arrest, which is known as a tumor-suppressive effect [44]. Additionally, overexpression, mutation, or pharmacological activation of NOTCH [45], together with mutations in zinc finger homeobox 3 (*ZFHX3*) [46], can enhance sensitivity to immunotherapy by activating the STING pathway and up-regulating major histocompatibility complex (MHC) class I molecules.

In SCLC, abnormal expressions of epigenetic modifying enzymes such as DNA methyltransferases (DNMTs) 1 to 3, or histone demethylases/methyltransferases such as lysine (K)-specific demethylase 6A (KDM6A), lysine methyltransferase 2A (KMT2A), and lysine-specific demethylase 1 (LSD1), regulate target gene expression through enhancer-, transcription factor (TF)-, or promoter-dependent or independent mechanisms [47], thereby contributing to cancer initiation [46]. Additionally, the loss of immune constraints or surveillance mechanisms, in conjunction with metabolic reprogramming and neurogenic factors [48], like stathmin 2-driven perineural invasion [49], plays a critical role in promoting the catastrophic development of SCLC. Notably, metastasis and treatment resistance in SCLC also share most of these abnormal regulatory mechanisms [50], with differences likely to be found only in terms of degree and scope.

### Potential mechanisms in the development and metastasis of SCLC

Metastasis of SCLC is a systemic process of cancer cells adapting to a dynamically changing microenvironment, such as hypoxia and nutrient deprivation, or environments rich in hydrogen peroxide [51], detaching themselves from the primary tumor, and then entering into circulation [52] to colonize distant organs. In general, the dominant mechanism involves the clonal evolution of cancer cells, or trans-differentiation among different cell lineages—termed phenotypic plasticity, encompassing the transitions in molecular subtypes, cellular or tissue types, as well as EMT, hallmarked by the loss and acquisition of NE and stem-like features [53]. Additionally, cancer cells can re-educate surrounding cells to gain supportive signals and evade immune surveillance. At the mechanistic level, this process involves multiple layers of regulation, including long-term and complex genetic and epigenetic reprogramming across all related cell types [54]. Most notably, not all the metastatic mechanisms discussed here are solely based on evidence derived from SCLC, as research on SCLC remains challenging due to its aggressive nature and the limitations of experimental models [54].

An early step in the acquisition of migratory and invasive capabilities by cancer cells, although not universally observed [55], is EMT, characterized by the loss of epithelial basement polarity, reduced cell–cell adhesion, and enhanced motility, which can occur even prior to the diagnosis of the primary tumor. EMT is a gradual process that proceeds through multiple tumor subpopulations toward a fully mesenchymal state, driven by a set of core TFs, including snail family transcriptional repressor 1/2 (*SNAIL1/2*), *TWIST1/2* (bHLH family), *YAP*, and zinc finger E-box-binding homeobox 1/2 (*ZEB1/2*) [56,57]. In contrast, loss of function of *FAT1* [46] leads to the suppression of epithelial-related genes, such as E-cadherin and other adhesion molecules, while promoting the expression of mesenchymal markers, including vimentin, fibronectin, and N-cadherin. This is accompanied by the secretion of factors associated with extracellular matrix degradation [58,59]. Most importantly, the neuronal-like features of SCLC—capable of mimicking the behavior of neuroblasts by forming long, axon-like protrusions (typically 50 to 100 μm) [55], as well as exhibiting electrical activity [60]—confer a selective advantage for cell migration [61], extravasation, and intravasation. In addition, the up-regulation of high mobility group protein box 3 (*HMGB3*), tropomyosin-related kinase B receptor (*TRKB*) [62], and L1 cell adhesion molecule (*L1CAM*) [63], along with the down-regulation of caspase-10 (*CASP10*) [46], Ras GTPase-activating protein 4 (*RASA4*) [64], and E-cadherin (*CDH1*), as well as noncoding RNAs [57], induces EMT and contributes to SCLC metastasis. Within the TME of SCLC, crosstalk among cancer-associated fibroblasts (CAFs), peripheral innervation, and cancer cells also influences tumor plasticity [60], thereby promoting metastasis [54].

The multi-organ metastasis of SCLC also involves epigenetic regulation [65]. Increased chromatin accessibility may be regulated by C-X-C chemokine receptor type 4 (CXCR4)/forkhead box m1 (FOXM1)/ribonucleotide reductase M2 (RRM2) activation [66], nuclear factor I B (*NFIB*)-dependent coactivator-associated arginine methyltransferase 1 (*CARM1*) overexpression [67], or an alternative NFIB-independent transcriptional network, which can be activated through the cooperation of forkhead box A 1/2 (*FOXA1*/*2*) with ASCL1 [68], thereby permitting the up-regulation of pro-metastatic programs. Similarly, mutations in DNMT1–3 or KMT1–3, leading to hypomethylation of DNA and histones, which activates metastasis-promoting genes such as myeloid ecotropic viral integration site/hox (*MEIS/HOX*), or the suppression of poly(adenosine diphosphate-ribose) polymerase 1 (PARP1) ubiquitination, maintaining DNA damage repair capacity [69], all contribute to the progression of SCLC.

In the EMT process of SCLC cells [70], an immunosuppressive microenvironment may also be established, characterized by the up-regulation of programmed cell death ligand 1 (PD-L1) and B7-H3 (also known as CD276) [71], accompanied by the secretion of various cytokines such as transforming growth factor-β (TGFβ), interleukin-6 (IL-6), tumor necrosis factor (TNF), and interleukin-8 (IL-8), as well as chemokines. At the same time, antigen-presenting related genes, including MHC-I molecules, STING, and interferon (IFN) pathways, are down-regulated [50], leading to reduced infiltration and function of CD8^+^ T cells, dendritic cells (DCs), and natural killer (NK) cells [71]. In turn, immunosuppressive cells such as regulatory T cells (Tregs) and myeloid-derived suppressor cells (MDSCs) are recruited by caspase 8 [72] and activated upon taking up extracellular vesicles containing mitochondrial DNA secreted by cancer cells [73], with immature tissue-resident macrophages polarizing toward an immunosuppressive M2 phenotype, and secreting TGFβ and/or IL-6, as well as nitric oxide synthase 2 (NOS2). These induce a robust EMT and display hallmarks of a positive feedback loop between EMT and myeloid cell recruitment [70]. Notably, infiltrating monocytic MDSCs promote the spread of tumor cells from the primary site by inducing EMT/CSC phenotypes, whereas granulocytic MDSCs support postmetastatic growth by reversing the EMT/CSC phenotypes. Therefore, the origin and spatiotemporal distribution of MDSCs may confer distinct functional roles in tumor progression.

In the hypoxic TME, in addition to endothelial cell-driven angiogenesis induced by various cytokines such as vascular endothelial growth factor/receptor (VEGF/R) and dopamine/R [74], cancer cells themselves can mimic endothelial cell functions, forming an independent vascular network known as vasculogenic mimicry, which promotes the invasion and metastasis of SCLC [75]. Furthermore, NOTCH-activated non-NE SCLC cells also possess the capacity for vasculogenic mimicry, characterized by integrin β1-mediated remodeling of the extracellular matrix and the formation of hollow tubular structures [75].

Upon entry into the vasculature, SCLC-derived CTCs (≥50 CTCs/7.5 ml) exist predominantly as single cells with a mesenchymal phenotype. However, less than 30% may aggregate through intercellular adhesion mediated by cellular protrusions [76], or form clusters that are enveloped by sinusoidal endothelial cells or platelets, traveling alongside tumor-associated fibroblasts, macrophage-like cells, neutrophils, and polymorphonuclear MDSCs. These clusters not only provide a survival mechanism against anoikis but also contribute to immune evasion and resistance to therapeutic interventions [77].

Evidence has shown that, before the arrival of disseminated tumor cells (DTCs), cancer cells secrete tumor-derived mediators (TDMs) either directly or by encapsulation in microvesicles, such as soluble cytokines, chemokines, growth factors, free RNA, and metabolites, modulating the extracellular matrix, including the proteoglycan versican, or hijacking other immune cells and CAFs to form a premetastatic niche (PMN) in distant tissues [78,79]. The features of this niche minimally include up-regulation of angiogenic factors and fibronectin, which activate bone marrow-derived endothelial progenitor cells expressing VEGFR1/2 [80], thereby inducing vascularization [81]. TDMs also actively recruit MDSCs into distant organs, and even lymph nodes [82] trigger immunosuppressive functions of resident monocytes, macrophages, neutrophils, and regulatory DCs [83]. Additionally, they stimulate fibroblast-driven matrix remodeling and elevate prostaglandin E2 (PGE2) production [84], which collectively suppresses immune effector cell function.

After DTCs infiltrate distant organs, beyond inducing MET, they also interact with various microenvironmental components [85] and myeloid-derived factors, such as triggering receptor expressed on macrophage subsets, thereby inhibiting the recruitment and activation of T cells and NK cells [86,87]. In addition, tumor cells express inhibitory ligands, such as PD-L1, which directly suppress effector T (Teff) cell function, or lose or down-regulate antigen-presenting pathways, such as MHC-I and IFN-I signal pathways, to escape recognition and attack by T cells. As a result, DTCs are able to evade immune surveillance and maintain organ-specific metastatic populations. Notably, chronic and persistent STING signaling driven by genomic instability and chromosomal abnormalities, along with increased endoplasmic reticulum stress [88], generates an immunosuppressive TME [89] enriched in M2 macrophages and granulocytic MDSCs, which consequently leads to T cell exhaustion featured by impaired antitumor function [88].

Metastasis has long been considered to occur in a nonrandom pattern. Specifically, organ-specific metastatic dissemination in SCLC involves key survival- and phenotype-regulating molecules, including NFIB, PDGFRB, and ANGPTL4 in brain metastasis, β3-integrin [90], CXCR4, and iron-transporting macrophages [91] in bone metastasis, and the periostin–NOTCH1 signaling in liver metastasis [92]. Furthermore, the differences in the metabolic reprogramming of cancer cells, such as the regulation of glutaminase (GLS1) and phosphoribosyl pyrophosphate amidotransferase (PPAT), combined with the availability of nutrients in distant organs, may confer a selective advantage for metastatic cells to colonize and proliferate in specific sites [93]. Regarding SCLC brain metastasis, this intracranial invasion of cancer cells may be attributed to assistance from brain-resident macrophages (namely, microglia) [94], which are mediated by tumor-secreted inflammatory cytokines or miRNA-containing exosomes that can activate the CCL2–CCR2 signaling pathway [95]. Furthermore, glutamatergic and gamma-aminobutyric acid (GABA)-ergic cortical neurons within the brain form authentic synaptic connections with SCLC cells [61], or act through paracrine signaling to induce membrane depolarization of tumor cells [96,97], thereby driving the proliferation of SCLC in the brain. Similarly, cancer cells up-regulate neuronal programs—referred to as neuronal mimicry—by secreting brain developmental factors such as Reelin, which recruit astrocytes. In turn, astrocytes promote the growth of SCLC by secreting neuronal survival factors, such as serpin family E member 1 (SERPINE1) [98]. In addition, the unique blood–brain barrier-like vascular gate (BVG) structures inherent to cancer cells [94], along with the simultaneous induction of M2 polarization of microglia or activation of astrocytes—such as through tissue inhibitor of metalloproteinases-1 (TIMP1) [99] secreted by cancer cells—collectively impair T cell migration, activation, and proliferation, thereby promoting the formation of immunologically desolate ecotypes, which may underlie the high prevalence of brain metastasis in SCLC. However, microglia also facilitate the recruitment and activation of T cells and NK cells, highlighting the importance of understanding the unique immunobiological mechanisms within the central nervous system (CNS) in developing specific antitumor strategies against brain metastasis [100]. Overall, the organotropism of SCLC brain metastasis relies on an interactive network of cancer cells–astrocytes–neurons [98], which is regulated through various signaling pathways. Although the genetic profiles of SCLC metastatic sites are highly similar to those of the primary tumors, high TMB and mutations in PTEN are more frequently observed in brain metastases, and key differences are also evident in the expression of phospholipase C γ2 (*PLCG2*), *DLL3*, and *NEUROD1* [101–103], suggesting environmental dependency and variability in metastatic regulation.

Disseminated cancer cells, in the absence of a supportive microenvironment, can enter a reversible state of cell cycle arrest or quiescence that may persist for years, while retaining their EMT phenotype [104]. Accumulated evidence suggests that this quiescent state is driven by interactions between cancer cells, immune cells, and stromal cells, involving genetic and epigenetic reprogramming of each cell type. Not only does this provide cancer cells with a survival or metastatic advantage but also contributes to the development of treatment resistance [105,106].

### Subtyping of SCLC

The subtyping of SCLC is a gradually deepening and complex endeavor from macroscopic to microscopic levels, witnessing various achievements in SCLC research (Fig. 3). Approximately 40 years ago, 2 SCLC phenotypes—classic and variant—were described based on morphology and growth behavior [107]. Subsequently, they were further classified into high-NE or low-NE subtypes (the definition may vary slightly when compared to the NE or non-NE subtype across most studies) based on the expression of markers [22], with the latter exhibiting more mesenchymal features, even though both subtypes typically involve inactivation of the *RB1* and *TP53* tumor suppressor genes. High-NE SCLC is characterized by low immune cell infiltration, whereas low-NE SCLC is associated with increased immune cell infiltration and is referred to as an “immune oasis” [108]. SCLC exhibits marked heterogeneity, serving as both the basis and the rationale for subtyping. However, due to the dynamic spatial and temporal regulation in the TME—including genomic (intrachromosomal or extrachromosomal), transcriptomic, proteomic, and immune-related features [37,54,102,109], molecular subtypes often display significant ambiguity and inconsistency, both within and across subtypes.

**Fig. 3.**
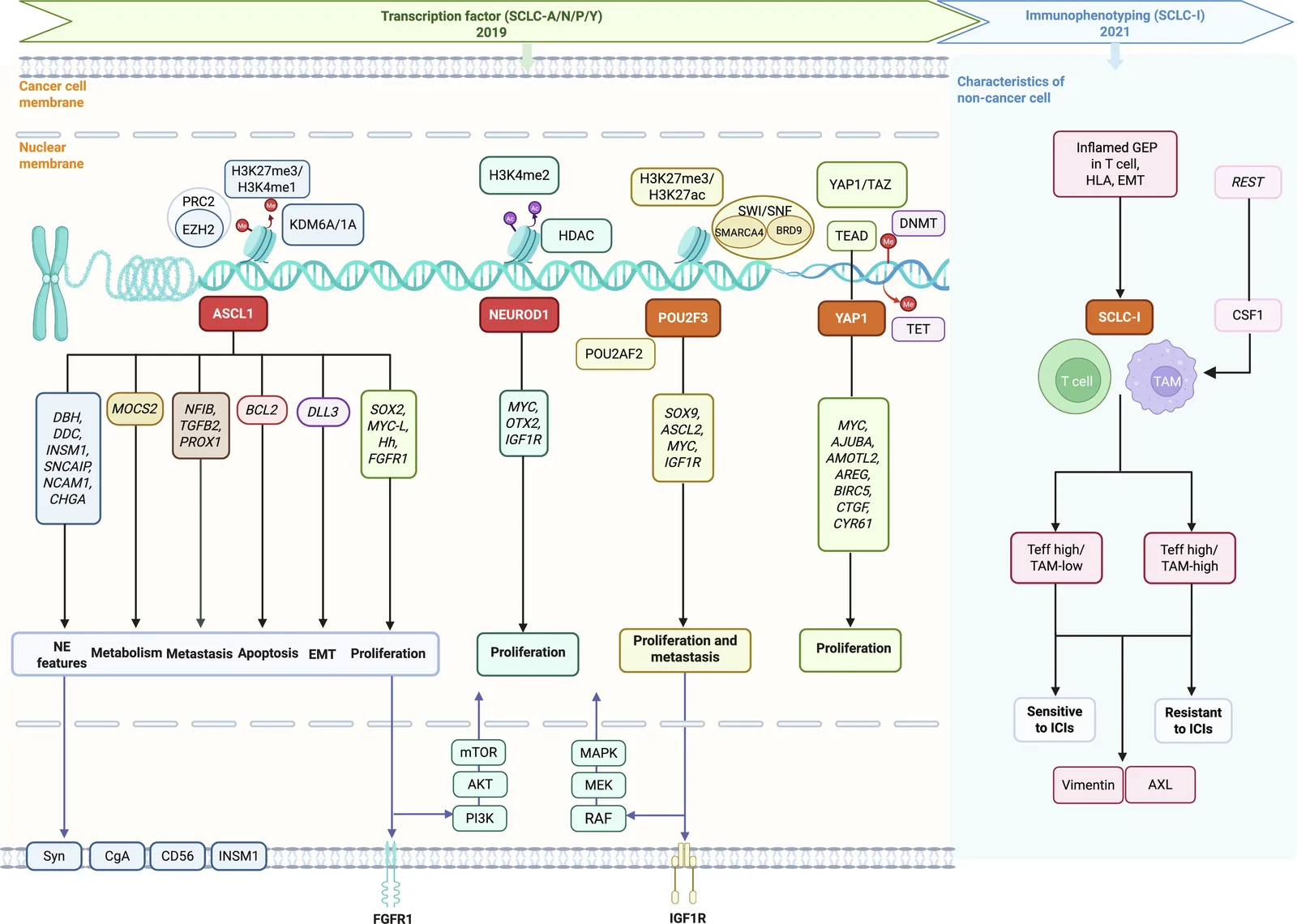
Comprehensive classification of SCLC subtypes. Transcription factors regulate downstream target genes; however, their regulatory networks are highly interconnected and exhibit extensive crosstalk. Immune subtyping is mainly determined by the phenotypic features of immune cells, and therefore exists in parallel with transcriptional subtyping, thereby also contributing to the possibility of cross-association. The molecules depicted in the figure may be shuttled across the nucleus, cytoplasm, or cell membrane, depending on their synthesis, functional engagement, and subsequent degradation or recycling, rather than being confined to a fixed subcellular localization. The term “extracellular to the cancer cell” refers to the immune microenvironment surrounding the cancer cell, but located outside of it. This figure was created using BioRender. ASCL1, achaete-scute homolog 1; *AJUBA*, Ajuba LIM protein; *AMOTL2*, angiomotin-like 2; *AREG*, amphiregulin; *ASCL2*, achaete-scute complex homolog 2; *BCL2*, B cell lymphoma 2; *BIRC5*, baculoviral IAP repeat-containing protein 5; BRD9, bromodomain-containing protein 9; *CHGA*, chromogranin A; CgA, chromogranin A; CSF1, colony-stimulating factor 1; *CTGF*, connective tissue growth factor; *CYR61*, cysteine-rich 61; *DBH*, dopamine β-hydroxylase; *DDC*, dopa decarboxylase; *DLL3*, delta-like ligand 3; DNMT, DNA methyltransferase; EMT, epithelial–mesenchymal transition; EZH2, enhancer of zeste homolog 2; FGFR1, fibroblast growth factor receptor 1; GEP, gene expression profile; H3K4me2, histone H3 lysine 4 dimethylation; H3K27ac, histone H3 lysine 27 acetylation; H3K27me3, histone H3 lysine 27 trimethylation; HDAC, histone deacetylase; Hh, Hedgehog signaling; HLA, human leukocyte antigen; H3K27; histone H3 lysine 27; *IGF1R*, insulin-like growth factor receptor; ICIs, immune checkpoint inhibitors; *INSM1*, insulinoma-associated protein 1; KDM6A, lysine demethylase 6A; MAPK, mitogen-activated protein kinase; Me, methylation; MEK, MAPK kinase; *MOCS2*, molybdenum cofactor synthesis 2; mTOR, mechanistic target of rapamycin; *MYC*, MYC proto-oncogene; NEUROD1, neurogenic differentiation factor 1; *NCAM1*, neural cell adhesion molecule 1; NE, neuroendocrine cell; *NFIB*, nuclear factor I B; *OTx2*, orthodenticle homeobox 2; POU2F3, pou class 2 homeobox 3; PI3K, phosphoinositide 3-kinase; PRC2, polycomb repressive complex 2; *PROX1*, prospero homeobox 1; RAF, rapidly accelerated fibrosarcoma kinase; *REST*, RE1-silencing transcription factor; SWI/SNF, Switch/Sucrose Non-Fermentable; SCLC, small cell lung cancer; *SOX2*, SRY-box transcription factor 2; *SOX9*, SRY-box transcription factor 9; Syn, synaptophysin; SCLC-I, small cell lung cancer-inflamed subtype; TAM, tumor-associated macrophage; TAZ, transcriptional coactivator with PDZ-binding motif; TEAD, transcriptional enhanced associate domain; TET, ten-eleven translocation; *TGFB2*, transforming growth factor β2; *YAP1*, yes-associated protein 1.

Transcriptional epigenetics plays a central role in the intricacies of SCLC, governing gene expression through a myriad of sophisticated control mechanisms at diverse hierarchical levels. Specifically, histone modifications include histone methylation [mediated by polycomb repressive complex 2 (PRC2) components such as SUZ12, EED, and EZH1/2, as well as KMT2, KMT3, KMT4, euchromatic histone lysine methyltransferase 2 (EHMT2), and myeloid-Nervy-DEAF-1 (MYND)], histone acetylation (such as H3K27Ac), and enzymes involved in phosphorylation and ubiquitination. Additionally, histone deacetylases (HDACs) and histone demethylases (such as KDM1A/2/4/5/6 and Gis1p) also play important roles, influencing the 3D structure and accessibility of chromatin. In SCLC, several key TFs, including ASCL1, NEUROD1, POU2F3, and YAP1, also orchestrate gene-expression programs. In addition, the regulation of gene expressions involves DNA methylation of the genes themselves—which is modulated by DNMT1/3 and enzymes that reverse DNA methylation, such as ten-eleven translocation (TET) 1–3—as well as gene loss, amplification, mutation, and rearrangement. The transcribed mRNA can also be posttranscriptionally modified, such as through N6-methyladenosine (m6A) modification, which is regulated by various methyltransferases, demethylases, and m6A-binding proteins. These modifications rapidly reshape the transcriptome and proteome of both cancer and immune cells [110]. Furthermore, besides noncoding RNAs such as miRNAs, circular RNAs (circRNAs), and long noncoding RNAs (lncRNAs) [111] that regulate RNA-to-amino-acid translation, there are some RNAs that also directly bind to proteins, control protein oligomerization, and regulate enzyme activity, or simply act as scaffolds in metabolic pathways, promoting the assembly of metabolons [112]. In SCLC, extensively characterized TFs and transcriptional processes govern the lineage-specific traits of cancer cells, as well as other biological processes—many of which overlap with those of noncancerous cells, including immune populations, constituting a major bottleneck for precision targeted intervention.

In 2019 [23], based on the differential expression of key neurotrophic TFs—*ASCL1*, *NEUROD1*, *POU2F3*, and *YAP1*—SCLC was classified into molecular subtypes, including SCLC-A, SCLC-N, SCLC-P, and SCLC-Y, which accounted for 70.0%, 11.0%, 16.0%, and 2.0%, respectively [4,113]. These proportions show minor variations across different populations and samples. Recent immunohistochemical studies have identified a quadruple-negative SCLC subtype (SCLC-QN) characterized by low or absent expression of the 4 TFs [114], as well as rare human papillomavirus (HPV)-positive cases. However, whether these represent a distinct genetic subtype remains to be confirmed [13]. Additionally, the studies identified SCLC-Aα, SCLC-Aσ [115], and SCLC-A2 (NEv2) subclusters, as well as a novel atonal homolog 1 (ATOH1) subtype [116].

ASCL1 and NEUROD1 are 2 basic helix-loop-helix (bHLH) TFs that act as major regulators of NE differentiation in SCLC [117], and they promote the survival, proliferation, and migration of cancer cells. In contrast, the deletion of ASCL1 significantly inhibits tumor initiation and blocks progression to the NEUROD1^+^ SCLC subtype [118], leading to it also being referred to as an oncogenic driver. Clinical data indicate that the SCLC-A and SCLC-N subtypes are associated with high chemosensitivity [23], with SCLC-A showing a more pronounced response [117] despite ASCL1 overexpression being a poor prognostic marker in early SCLC. Overall, SCLC exhibits stronger immune exclusion and reduced immune infiltration, with the SCLC-N subtype with high DLL3 expression [119], showing a more pronounced immunosuppressive phenotype compared to SCLC-A. However, a subset of SCLC-A was identified that exhibits immune infiltration by NK and T cells, which indicated an immune-responsive profile [120].

In SCLC, one of the key phenotypic regulatory mechanisms is the inactivation of KDM6A, which leads to increased levels of H3K27me3 and decreased levels of enhancer-associated H3K4me1. This specifically induces a plasticity shift from ASCL1 to NEUROD1, resulting in the formation of SCLC subtypes that coexpress both ASCL1 and NEUROD1 [121]. Similarly, in ASCL1- or NEUROD1-expressing cells, inhibition of LSD1 (KDM1A) or circRMST, a circRNA [122], activates the NOTCH pathway while suppressing ASCL1-associated NE features. The inhibition of the SMARCA4 catalytic subunit of the Switch/Sucrose Non-Fermentable (SWI/SNF) complex, which blocks chromatin accessibility at ASCL1 and NEUROD1 loci, or the up-regulation of lamin A/C (*LMNA*) [123] may promote a transition toward a non-NE phenotype. These alterations may exert synergistic effects with ICIs [124]. Conversely, the up-regulation of the secretory neuropeptide precursor VGF or the depletion of capping protein inhibiting regulator of actin dynamics (*CRACD*) [125] in SCLC leads to the promotion of NE differentiation and escape from immune surveillance [126].

Similarly, ASCL1 per se regulates multiple downstream pathways, including NE-related genes, neurodevelopmental related genes, as well as NOTCH pathway, and metabolic regulatory pathways. On the contrary, ASCL1 loss in conjunction with high MYC and PTEN loss promotes the development of tuft-like tumors, characterized by POU2F3^+^ and YAP1^+^, as well as subtype-unclear populations enriched for proliferative/stem-like or mesenchymal signatures. All of these are involved in phenotype plasticity, EMT, and stem cell characterization, together with antigen presentation and processing. In contrast, NEUROD1-regulated transcriptional targets include insulin-like growth factor 1 receptor (*IGF1R*) [127,128], the receptor *TRKB*, and neural cell adhesion molecule 1 (*NCAM1*). Collectively, these genetic and epigenetic alterations represent potential therapeutic vulnerabilities in SCLC, although the functional significance of many of these regulatory networks has not been completely elucidated.

POU2F3 defines a non-NE subtype characterized by low expression of canonical NE markers such as thyroid transcription factor-1 (TTF-1) and DLL3. However, this subtype occasionally exhibits reduced levels of gastrin-releasing peptide (GRP) and calcitonin-related polypeptide α (CALCA, which encodes CGRP), tuft cell-specific expression of *SOX9* and achaete-scute homolog 2 (*ASCL2*) [22,23,117], and IGF1R. These features have been associated with improved survival outcomes. In POU2F3-positive SCLC, the cofactor POU2AF2 (also known as C11orf53) is recruited to chromatin [129], where it functions to enhance H3K27 acetylation (H3K27ac) and chromatin accessibility at enhancer regions through engagement of the SWI/SNF complex [130]. Conversely, disruption of SWI/SNF adenosine triphosphatase (ATPase) subunits SMARCA4/2 or the bromodomain-containing protein 9 (BRD9) suppresses tumor cell proliferation. These findings suggest that the SWI/SNF complex serves as a critical survival dependency in the non-NE subtype—a role potentially distinct from its involvement in immune regulation observed in the NE subtype [131].

Another non-NE SCLC subtype is defined by YAP1 expression. This subtype exhibits low levels of NE markers, but elevated expression of laminins and integrins, and has been associated with chemotherapy resistance and poor prognosis [132]. YAP1 is activated through mutations or dysregulation of upstream regulators such as Wnt family ligands, IGF-1, large tumor suppressor 1 and 2 (LATS1/2), and G protein-coupled receptors (GPCRs), and then, by binding to transcription factors, regulates downstream growth signaling pathways and acts as an oncogene [133,134]. Most notably, YAP1 is rarely detected in clinical specimens or xenografts [114,117], and YAP1-expressing SCLC cells are difficult to distinguish from primary undifferentiated thoracic tumors, as both share SMARCA4 deficiency [135] and overlapping clinical and histopathological features, all of which lead to controversy regarding the YAP1 subtype. However, recent studies have shown that YAP1-positive cell populations significantly increase with treatment resistance, characterized by up-regulation of B7-H3 and trophoblast cell surface antigen 2 (TROP2), but lack of DLL3 and seizure-related 6 homolog (SEZ6), providing clear evidence to resolve the controversy [136]. SCLC-P and SCLC-Y, characterized by up-regulation of IGF-1 [133] and MYC [113]—especially the latter subtype—are sensitive to inhibition of aurora kinase A and B (AURK A/B) and MYC, and may also exhibit synergistic responses to combination chemotherapy [36].

Recently, a novel non-NE subtype—inflammatory SCLC (SCLC-I)—was identified [2] based on IFN-γ activation, human leukocyte antigen (HLA) typing, increased expression of immune checkpoints, and the association between EMT and immune-related gene expression [137], which may hugely benefit from immunotherapy combined with chemotherapy. Furthermore, SCLC-I can be further subdivided into 2 clusters: one with fewer tumor-associated macrophages (TAMs) and more Teff cells, associated with better clinical outcomes after chemotherapy and ICI treatment, and the other with more TAMs and Teff cells, which is linked to poor therapeutic response. New subtypes, such as relatively immune-desert non-negative matrix factorization 1 (NMF1) and NMF2, and relatively immune-inflamed NMF3 and NMF4—identified through NMF [138], or the immune hot group (IHG) and the immune cold group (ICG)—identified through immune-related gene-targeted transcriptomic analysis [139], show distinct responses to immunotherapy. These subtypes are associated with the NE state but independent of the TF subtype. However, most studies on SCLC have focused on the correlation between phenotypic transformation and prognosis, while research on the mechanisms of cancer cell transformation, expansion, and metastasis remains limited, thereby hindering the identification of specific drug targets.

Although the initiating cell determines the subtype of SCLC, during tumor progression or under treatment stress [46,138], different subclones communicate through paracrine signaling via the Fgf2 and mitogen-activated protein kinase (MAPK) pathways, or through epigenetic regulators or MYC activation, leading to the transformation of SCLC-A into SCLC-N or SCLC-Y dominant tumors [121,140], or vice versa [44,102]. As a result, SCLC is represented by a continuum of various molecular subtypes, capable of executing a wide range of functions—from proliferation and migration to metabolism, secretion, and immune evasion—thereby contributing to its overall heterogeneity and plasticity [53,103,141]. Moreover, non-NE cells support the action potential firing of NE cells metabolically, through a metabolic substrate shuttle similar to that between astrocytes and neurons, directly promoting the malignant progression of SCLC [60].

Therefore, molecular subtypes defined by discrete transcription factors or other biological features in primary tumors may not fully reflect the complexity of the disease [53], and using them to guide clinical treatment may be impractical [142]. The phenotypic plasticity of SCLC [143] underscores the need for periodic deconvolution of ctDNA or CTCs—that is, liquid biopsy—combined with single-cell transcription factor sequencing (scTF-seq) [144] and radiomic data to more precisely characterize and track the evolution during treatment [145]. Nevertheless, tissue samples still remain the gold standard, as they can address the covariation of immune cells and stroma, and provide valuable clinical insights for mechanistic research—so-called reverse translation [146]—to identify novel therapeutic targets, such as ATPase family AAA domain-containing protein 2 (ATAD2) [146].

Secondly, the current transcription factor-based classification of SCLC remains functionally ambiguous. This is because the regulation of protein function—in terms of both quality and quantity—involves, beyond transcription factors, a wide range of other regulatory mechanisms, such as various genetic abnormalities upstream, and posttranslational modifications downstream. Which mechanism dominates has yet to be clarified. Moreover, downstream genes are subject to nonspecific crosstalk regulated by transcription factors, as they are extensively scattered across diverse pathways and within both normal and cancer cells. Additionally, these target genes may reciprocally alter the levels and functionality of transcription factors. Notably, although recent DNA methylation profiling based on cell-free DNA (cfDNA) has shown comparable subtyping performance [147], clustering still largely relies on transcription factor expression profiles.

Finally, the definition of cell phenotypes may also depend on other parameters, such as proteomic, phosphoproteomic, or metabolomic profiles, as well as spatial positioning of cells and their interactions with other cell types within the TME, including immune cells [148], neuronal cells, and stromal cells [23]. This underscores the need to fully leverage AI technologies—integration of radiographic imaging [149] and digital pathological imaging [150] with molecular omics and immune signatures—to better understand the high variability and ambiguity of SCLC molecular subtypes driven by environment dependency and, in so doing, address the long-standing challenge of clinical integration. Recently, using a combination of imaging-based multiplexed detection (CODEX) and multi-omics analysis [151], a cell aggregate detection algorithm called ColonyMap was developed to define a spatial immune microenvironment composed of antitumor macrophages, CD8^+^ T cells, and NK T cells that would be strongly associated with improved immunotherapy response and favorable prognosis.

### Mechanisms of drug resistance in SCLC

Approximately 75% to 80% of untreated SCLC tumors are initially highly sensitive to DNA-damaging agents; however, resistance inevitably emerges rapidly [152], possibly driven by the plasticity of cancer cells, as well as surrounding accomplice, like immunosuppressive cells. This review primarily addressed the mechanisms of resistance to immunotherapy and chemotherapy, although other treatment approaches are also relevant (Fig. 4).

**Fig. 4.**
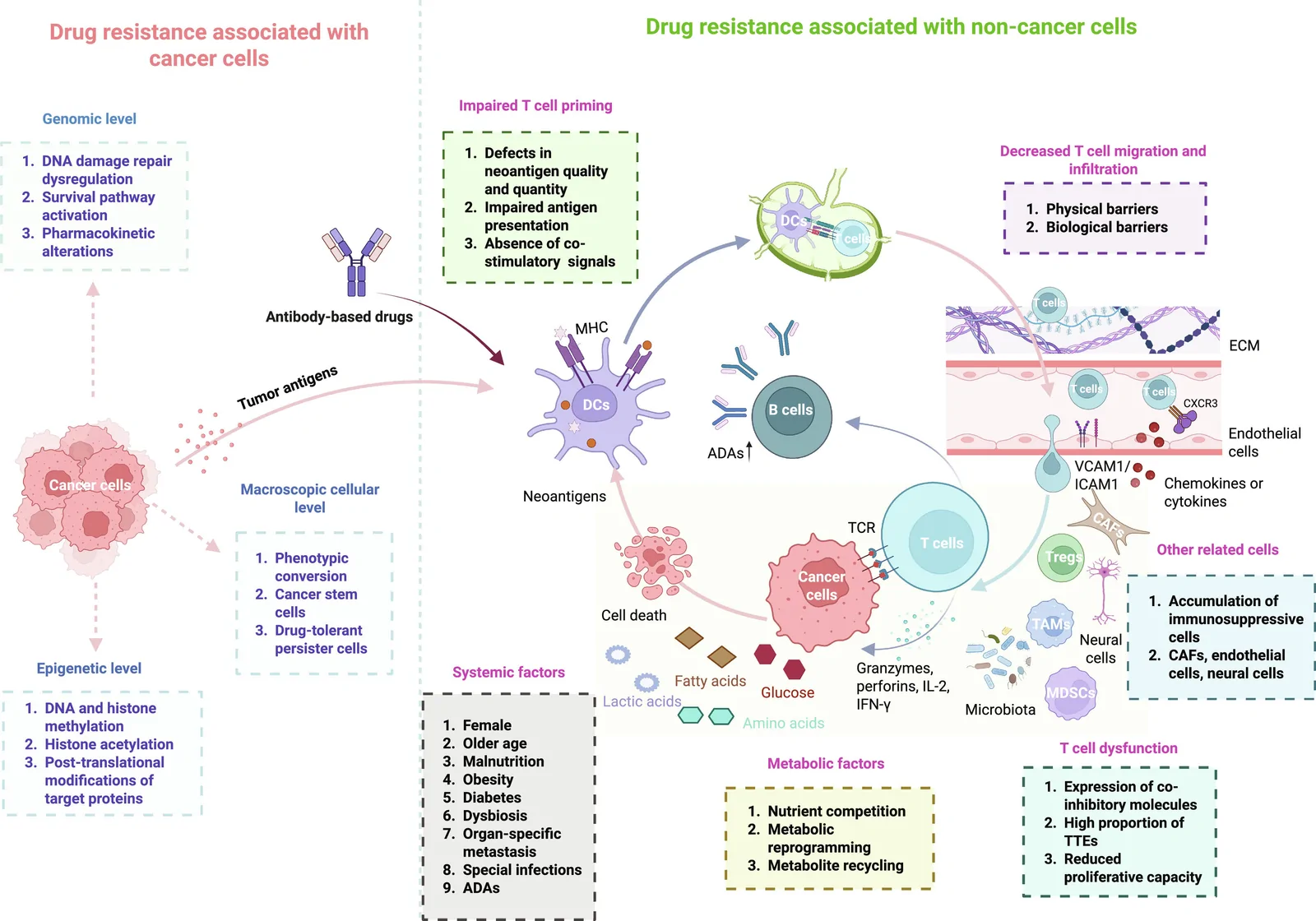
Mechanisms related to treatment resistance in SCLC. Drug resistance in cancer cells manifests as a multifaceted and redundant mechanism, with varying degrees and scopes of involvement that differ across distinct temporal, spatial, and patient-specific contexts. The emergence of resistance to chemotherapy and immunotherapy is not characterized by mutual exclusivity or parallel development, but is instead underpinned by a spectrum of interdependent and intertwined mechanisms. Furthermore, their combined therapeutic use, or the inclusion of additional modalities, serves to amplify this complexity of resistance. This figure was created using BioRender. ADAs, anti-drug antibodies; CAFs, cancer-associated fibroblasts; CXCR3, C-X-C motif chemokine receptor 3; DCs, dendritic cells; ECM, extracellular matrix; ICAM-1, intercellular adhesion molecule; IFN-γ, interferon-γ; IL-2, interleukin-2; MHC, major histocompatibility complex; MDSCs, myeloid-derived suppressor cells; TAMs, tumor-associated macrophages; TCR, T cell receptor; Tregs, regulatory T cells; TTEs, terminally exhausted t cells; VCAM, vascular cell adhesion molecule.

#### Drug resistance associated with cancer cells

Drug resistance in SCLC is a progressive process where cancer cells gradually enhance their adaptive capacity, resulting in a drug-resistant continuum driven by dynamic network changes involving gene interactions, coordination, and reconfiguration. The complexity of drug resistance is also reflected in the ambiguity regarding the degree of ineffectiveness of drugs or therapies, which is challenging to quantify, as clinical practice typically involves combination treatments [153].

##### Macroscopic cellular levels

SCLC cells exhibit significant plasticity and instability—transitioning from one established lineage/clone to another, which are the main causes of heterogeneity outbreaks within tumors [154] and also participate in drug resistance formation.

Before treatment, SCLC exhibits clonal homogeneity, characterized by the presence of one or more cell types marked by ASCL1, NEUROD1, or YAP1. However, under the influence of initial oncogenic drivers within the TME and extrinsic selection pressures such as chemotherapy and radiation [155], persistent output NOTCH and REST signaling may promote increased *MYCN* expression [156] and extrachromosomal DNA amplification of *MYC* [38]. This disrupts SWI/SNF function and alters the cellular fate trajectory of SCLC—shifting from an ASCL1-driven state to a NEUROD1-driven state, or toward epithelial–mesenchymal plasticity (EMP) [57], which provides a reservoir of drug-resistant cells [154,155], yet is accompanied by up-regulation of MHC class I molecules [137,157]. Therefore, mesenchymal phenotypic conversion may constitute a paradoxical biological state, characterized by chemoresistance but sensitivity to immunotherapy.

In addition, SCLC recurrence is driven by a subset of chemoresistant and immunoresistant progenitor/stem cell populations or quiescent cell populations that are in a state of proliferative arrest [158]. EphA2-mediated activation of MYC, CD44, and SOX2 [159], together with IL-1 release and the suppression of Hippo signaling [41], induces stem-like features that play a key role in the development of chemoresistance in SCLC. Other signaling cascades associated with CSCs, including, but not limited to, NOTCH, Hedgehog (Hh) [160], podocalyxin-like protein 1 (PODXL-1), patched (PTCH), CD87, and Wnt/β-catenin [161], also contribute to drug resistance. Indeed, the combination of the Hh inhibitor sonidegib with chemotherapy results in more durable treatment responses and improved clinical outcomes in SCLC patients. Moreover, the drug-tolerant persister (DTP) subpopulation, which harbors stem-like properties yet differs from canonical CSCs, may also drive therapeutic resistance in SCLC [162]. In summary, the identification of CSCs in SCLC remains challenging, and our understanding of mechanisms underlying treatment resistance is still limited.

##### Genomic levels

Drug resistance, especially to ICIs, in different SCLC subclones may arise from allelic loss in chromosomal regions such as 17p (harboring *TP53*), 5q (*APC*), and 13q (*RB1*), or allelic gain in regions such as 3q (*SOX2*) and 8q (*MYC*), as found in chromosomal copy number aberrations (CNAs) [163], or aneuploidy [164].

Firstly, up-regulation of pathways related to chromosomal or extrachromosomal [165] DNA damage repair contributes to drug resistance in SCLC. These include glutathione (GSH), PARP, and DNA repair regulator PRKDC (DNAPKcs) [166] involved in base excision repair (BER) and nucleotide excision repair (NER) [167,168], as well as high mobility group box 1 (HMGB1) [169]. Additionally, EZH2-induced methylation and down-regulation of DNA damage repair factors such as Schlafen11 (*SLFN11*) [170], as well as the loss of MSH2, MSH6, or USP22 [171], also contribute to the development of drug resistance in SCLC.

Secondly, processes related to cell survival, proliferation, and apoptosis also contribute to platinum resistance. These include the activation of the WNT signaling pathway [172], the kinase suppressor of Ras 1 (KSR1)-Raf/MAPK kinase (MEK)/extracellular signal–regulated kinase (ERK) signaling cascade [173], the PI3K/AKT pathway [174], and the CDH1high/Nfiblow signature, as well as the up-regulation of *YAP1* [41], lipoprotein receptor-related protein 1b (*LRP1B*), ryanodine receptor (*RYR2*), usherin *(USH2A*), *BCL2*, *TP53*, *TP73*, *MET*, *TWIST1*, *BAX*, X-linked inhibitor of apoptosis protein (*XIAP*), *MYCL*, *FMN2* [109], microtubule-associated serine/threonine kinase 1 (*MAST1*) [175], *MET* [176], S100 calcium binding protein A9 (*S100A9*) [177], and survivin [137]. In addition, the up-regulation of the ETS variant transcription factor 4 (*ETV4*) and *ETV5* in the FGFR–PEA3 signaling axis [178] or the interference of eIF6–CD104–FAK axis [179] induces chemoresistance in SCLC, whereas the differences in therapeutic response may vary depending on cellular origin background [180].

Thirdly, the drug resistance advantage of SCLC subclones may also be reflected in the molecular pathways involved in drug absorption, transport, efflux, and metabolism, such as the copper transporter 1 (CTR1), multidrug resistance protein (MDR) [181], P-glycoprotein (MDR1), and MDR-related proteins (MRP1 and MRP2), adenosine triphosphate (ATP) binding cassette subfamily C member 1 (ABCC1) (encoding a glycoprotein associated with the multidrug resistance phenotype), copper-transporting ATPases, and CYP3A4/CYP3A5 or uridine diphosphate (UDP) glucuronosyltransferase family 1 member A1 (UGT1A1).

Finally, in SCLC, defects in the quality and quantity of neoantigens, as well as abnormalities in antigen presentation mechanisms [182], contribute to resistance. Indeed, up to 85% of cases exhibit reduced expression of either MHC-I or MHC-II, with the NE subtype exhibiting the lowest levels [137]. In addition, the activation of oncogenic pathways, like Erb-B2 receptor tyrosine kinase 2 *(ERBB2*) [183], UBA1–STUB1 axis, or down-regulation of tumor suppressor genes in SCLC cells, along with the up-regulation of CD47 or annexin A1 [184], and secretion of immunosuppressive cytokines, such as IL-10, are associated in varying degrees with resistance to immunotherapy. Conversely, chromosomal instability and accumulation of DNA damage or genomic scars [145] induced by cytotoxic chemotherapy and radiotherapy, coupled with suppression of *FOXM1*, which alters AURKB signaling [185], can promote adaptive antitumor immune responses [186,187].

##### Epigenetic levels

In SCLC, dysregulation of pathways involved in DNA and histone methylation, such as PRC2–EZH2, DNMT1–3, KMT2A, and KMT2D (MLL and MLL2), as well as KDM4B [188], KDM6A, and LSD1/KDM1A, can alter gene expression or function in ways that may synergize with transcription factors such as ASCL1, NEUROD1 [121], REST, TCF2/HNF1B, NFIB, early flowering 3 (ELF3), BMX-E2F1 [189], and retinoic acid receptor β (RARB). These interactions, occasionally involving noncoding RNAs [190], regulate tumor suppressor genes like ras association domain family 1 (*RASSF1A*) [191] and *SLFN11* [170], as well as oncogenes such as *MYC* or *BCL2*, and are associated with resistance to chemotherapy or ICIs. Other histone-modifying enzymes that influence chromatin 3D structure or accessibility—including acetyltransferases CREBBP or CREB-binding protein (CBP) and EP300, PBRM1 (encoding BAF-180), and the chromodomain helicase DNA-binding protein 7 gene (CHD7), as well as bromodomain and extraterminal domain (BET) family molecules that recognize acetylated lysine residues [192]—are frequently mutated or up-regulated in human SCLC. These alterations contribute to drug resistance and can be reversed by HDAC inhibitors [31] or bromodomain inhibitors. In addition, epigenetic pathways also down-regulate the expression of MHC-I or other molecules in the MHC–STING pathway [193], leading to resistance to immunotherapy. In summary, although the abovementioned drug resistance-related alterations are widespread in SCLC and often interact with each other, they do not represent easily targetable therapeutic opportunities, even though there are some unique pathways and molecules, such as DLL-3 [152], that are exceptions.

#### Drug resistance associated with immune cells

The TME is an evolving, adaptive, and memory-capable ecosystem [194], characterized by a unique structural and physical profile. As a whole, the TME is immunosuppressive, dynamically shaped by tumor cells and nontumor components—primarily tumor-associated immune cells (including various T cells, B cells, granulocytes, macrophages, DCs, MDSCs, and NK cells), CAFs, the extracellular matrix, vascular endothelial cells, and a variety of soluble molecules such as pro-inflammatory or anti-inflammatory cytokines, growth factors, and chemokines [195], which contributes to immune evasion by cancer cells and therapeutic resistance.

Retrospective studies of samples from SCLC patients prior to the introduction of ICIs found that patients with high tumor-infiltrating lymphocytes (TILs) had better prognoses. Under prolonged antigen exposure, proteotoxic stress response [196], or the up-regulation of STUB1–CHIC2 and thymic high mobility group box (TOX), naive T cells gradually formed distinct-fate T cells with impaired function driven by fine-tuning alterations in epigenetic [197] and transcriptional programming. These include precursor exhausted T cells (TPE), early exhausted T cells, and terminal/late exhausted T cells (TTE), characterized by the up-regulation of co-inhibitory molecules. Notably, the exhaustion of CD8^+^ T cells is a progressive state, and the functional status of exhausted T cells should not be regarded as inert, because early exhausted T cells retain substantial expression of effector function-related genes, such as perforin 1 (*PRF1*) and granzyme B (*GZMB*), suggesting that they may serve as potential therapeutic targets for ICIs. Indeed, in clinical trials combining chemotherapy and immunotherapy, such as IMpower133 [198] and CASPIAN [199], not all patients with inflammatory subtypes or POU2F3 non-NE subtypes responded equally well to treatment. This also explains, at least in part, why the number of TILs alone cannot fully predict response to immunotherapy. Admittedly, in addition to CD8^+^ T cells, extensive preclinical and clinical data support that deficiencies in the quantity and function of CD4^+^ helper T cells, CD4^+^ cytotoxic Teff cells, NK cells [200,201], invariant NK T cells, γδ T cells, DCs (DNASE1L3 positive), or inflammatory monocytes [202] are also correlated with immunotherapy resistance.

T cells must first be activated by antigens presented to them by antigen-presenting cells within secondary lymphoid organs before infiltrating the TME, eventually leading to the formation of tumor-infiltrating T cells and exerting their antitumor effects [203]. In fact, accumulating clinical data support an association between extensive lymph node dissection and poor prognosis or reduced response rates to ICIs. However, there is currently no large-scale clinical evidence supporting this association in SCLC. Therefore, physical barriers within the TME, such as increased cancer cell stiffness, extracellular matrix deposition and cross-linking, and elevated interstitial pressure, as well as biological barriers, such as insufficient chemokine secretion by immune cells, limited cytokine signaling, the lack of chemokine receptor expression on T cells, and endothelial cell anergy, may lead to difficulties in T cell infiltrating. Additionally, the inappropriate expression of ligands or chemokines by tumor cells and vascular STING signaling [201] can collectively hinder the recruitment and penetration of T cells or NK cells into the TME or even promote their egress from the TME [204]. In certain contexts, infiltrating CD8^+^ T cells, CD4^+^ helper T cells, and CD20^+^ B cells can assemble into tertiary lymphoid structures, which potentiate the therapeutic response to ICIs [205]. Conversely, immune-regulatory cells that maintain host homeostasis and immune balance, such as CD4^+^ Tregs, mast cells, eosinophils, MDSCs, and lymphatic endothelial cells (LECs) [206], can be hijacked or reprogrammed by cancer cells within the TME to initiate immune evasion and support cancer cell survival.

Myeloid cells represent the most abundant nucleated hematopoietic cells in the human body, comprising the 3 terminally differentiated cell populations of macrophages, DCs, and granulocytes. Tumors not only give rise to an elevated number of immature myeloid cells, but they also have the capacity to recruit [207] and convert these into potent immune suppressive cells, commonly known as MDSCs [208], which impede T cell trafficking, proliferation, and effector functions [209]. Specifically, the up-regulation of M2-TAM (predominantly CD68^+^/CD163^−^) function and migration may represent an important determinant of immunotherapy resistance in certain subtypes of SCLC [138], which may involve cancer cell secretion of various cytokines, such as erythropoietin (EPO) [210], to promote the expressions of *REST*, *MYC*, and *ZEB2* [211] as well as IL-33 [212]. In contrast, M1- or IFN-TAMs [213] exhibit opposing immunostimulatory functions.

Treg cells, whose discovery and characterization were honored with the 2025 Nobel Prize in Physiology or Medicine, represent a functionally and phenotypically highly heterogeneous population, which can be divided into resting (sometimes termed naive) Tregs, effector Tregs, inflammatory Tregs, and T follicular regulatory (TFR) cells. Most Tregs are capable of suppressing the functionality of Teff cells directly or indirectly, whereas a few Tregs can secrete pro-inflammatory cytokines, such as IFN-γ, thereby enhancing immune responses [214]. Indeed, an increase in the number of intratumoral Treg cells has been associated with a better prognosis [215]. Notably, other immune cells, such as neutrophils [216], macrophages, DCs, innate lymphoid cells (ILCs), innate-like T cells (ILTCs), and even NK cells [217], also exhibit diverse, partially redundant, and sometimes opposing functions. Therefore, in SCLC, a spatiotemporal perspective is imperative to further elucidate the roles of diverse immune cells as recipients or sources of activating and inhibitory signals [218], facilitating the development of targeted therapeutic strategies.

#### Drug resistance associated with other cell types

In addition to cancer cells and various immune cells, stromal cells, endothelial cells, smooth muscle cells, adipocytes, and neurons also contribute to immunotherapy resistance. Stromal cells, such as CAFs, consist of multiple subpopulations with distinct spatial organization patterns, neighboring cell compositions, interaction networks, and transcriptional profiles [219], and can not only directly support cancer cell survival by metabolic coupling [126] but also restrict immune cell infiltration and recruit immunosuppressive cell populations.

Over the past decade, several landmark reports have demonstrated that the CNS and peripheral nervous system (PNS), including the sympathetic nervous system, parasympathetic nervous system, and sensory nervous system, regulate tumor cell growth and metastasis—that is, neuroimmuno-oncology [220]. True neuron–cancer cell synapses in CNS, as well as neurotransmitters, neurotrophic factors, or miRNAs released by nerves in the PNS, or even SCLC cells directly hijacking synaptic signals [97], collectively may support the occurrence and invasion of cancer or interact with immune cells in the TME to impair immune surveillance [221]. Similarly, sustained inflammatory reactions triggered by tumor-induced neuronal injury (TINI)—initially intended to facilitate nerve repair and regeneration—can remodel the overall immune landscape of the TME into an immunosuppressive, exhaustion-prone phenotype, ultimately conferring resistance to immunotherapeutic regimens.

#### Drug resistance associated with metabolism

For more than 100 years, studies have shown that the proliferation, dissemination, and metastasis of cancer cells require high metabolic activity [93], which inevitably disrupts the local and systemic metabolic balance. This is further exacerbated by the production of large amounts of metabolic byproducts, leading to impaired antitumor functions of immune cells and the efficacy of immunotherapy [60,222]. Notably, the genes involved in metabolic pathways, as well as their genetic and epigenetic regulatory mechanisms, are likely to overlap and interact with the aforementioned drug resistance mechanisms, forming a redundant network of drug resistance. Moreover, the purpose of discussing drug resistance mechanisms from both cancer cell and noncancer cell perspectives is to enhance understanding, as the 2 are not strictly distinct but rather interconnected nodes within the immune circuit. For example, mitochondria harboring DNA mutations and attached by molecules that inhibit autophagy derived from cancer cells can be transferred to TILs, where they resist autophagy, leading to metabolic abnormalities, impaired effector function, and defective memory formation in T cells [223]. Cancer cells themselves, when combined with external stressors such as high-cholesterol diets, can independently or synergistically activate the expansion of RORγ-dependent MDSCs and M2-TAMs [224], thereby suppressing specific antitumor immunity.

##### Cancer cell-related metabolic pathways

Cancer cells adopt metabolic reprogramming featuring activated oncogenic signaling and elevated expression and activity of metabolic enzymes and functional proteins governing glucose, lipid and amino acid transport, catabolism, and anabolism [225]. Such metabolic rewiring not only sustains tumor cell viability but also endows them with superior nutrient competitive capacity against T lymphocytes, eventually inducing therapeutic resistance to immunotherapy and immunochemotherapy. Metabolic reprogramming associated with therapeutic resistance in SCLC also entails a shift from glucose-dependent metabolism to lipid-dependent metabolism [226]. Furthermore, some glycolytic enzymes possess noncanonical functions, namely, they can act as protein kinases, promoting transcriptional mechanisms that contribute to tumor growth and immune evasion, ultimately leading to treatment resistance [227].

##### Non-cancer cell-related metabolic pathways

Once activated, immune cells also undergo metabolic reprogramming to enable them to compete for nutrients with cancer cells. This precisely regulated process involves the glucose metabolism, amino acid transport and metabolism, and the uptake, transcription, or synthesis of lipid and cholesterol, along with mitochondrial respiratory function [228]. However, the immune effector cells are at a disadvantage, whether in terms of breadth or depth, which leads to impaired proliferation, migration, differentiation, and receptor signaling functions. Similarly, to exert immunosuppressive functions, MDSCs, N1/N2-tumor-associated neutrophils (TANs), or M1/M2-TAMs also meet their nutrient requirements through metabolic reprogramming, although there may be subtle differences [222]. Furthermore, in advanced tumors, the impaired migratory capacity of DCs [229], along with T cell dysfunction characterized by telomere [230] and mitochondrial damage, as well as reduced mannose metabolic capability [231], collectively attenuates antitumor immune responses.

##### Toxic metabolic products and their impact on immune cells

In the TME, large amounts of metabolites, such as lactate, produced through aerobic glycolysis in cancer cells, can be utilized by certain immune cells (such as Tregs and MDSCs) to promote their proliferation and maintain their immunosuppressive functions. In contrast, hypoxia, various metabolites, and their induced low pH, either alone or in combination, can not only directly inhibit the proliferation and cytotoxic function of NK cells [232], CD8^+^ memory T cells, and Teff cells but also promote the recruitment and activation of MDSCs, thereby disrupting antitumor effects [233,234]. Nevertheless, enhanced glycolytic activity induced by hypoxia-inducible factor-1α (HIF-1α) favors the formation of long-lived memory T cells, which potentially exerts antitumor activity [235]. A similar contradictory phenomenon is that prolonged exposure to succinic acid promotes the survival of CD8^+^ T cells and contributes to the generation and maintenance of stem-like subpopulations [236].

Overall, metabolic reprogramming influences the functional states of virtually all types of cells to enhance their survival, representing a key mechanism underlying tumor immune evasion and therapeutic resistance [237]. Importantly, however, truly cell-type-specific metabolic pathways are rare, and different cancer and immune cells display distinct dependencies on and sensitivities to various nutrients and metabolites [238]. Furthermore, enzymes and metabolites within diverse metabolic pathways often serve multiple or even opposing biological functions [42]. Finally, beyond intrinsic metabolic reprogramming, tumor cells leverage chronic inflammatory signaling—primarily through growth differentiation factor-15 (GDF-15), TNF, IL-1β, IL-6, and IL-8—to activate NF-κB and c-Jun N-terminal kinase (JNK) pathways, thereby driving angiogenesis, metastasis, immunosuppression, and therapy resistance. These systemic inflammatory signals also impact both the immune system and the hypothalamic neural circuits that regulate appetite, leading to reduced food intake and contributing to the development of cancer-associated cachexia [239].

#### Resistance induced by inherent properties of drugs and treatments

The inherent immunogenicity of antibody-based therapeutics—initially observed with murine-derived monoclonal antibodies—leads to the development of anti-drug antibodies (ADAs), representing a key mechanism underlying secondary resistance to chimeric antigen receptor T (CAR-T) cells, antibody–drug conjugates (ADCs), monoclonal or polyclonal antibodies, bispecific T cell engager (BiTE) molecules, or oncolytic viruses. However, whether nucleic acid vaccines are similarly affected remains unclear. Unfortunately, even fully human antibodies, humanized single-chain variable fragments (scFvs), or modifications in Fc glycosylation fail to completely eliminate immunogenicity and ADA formation. Regarding resistance to ADCs, the potential mechanistic underpinnings include hydrophobicity of ADCs, tumor penetration capability, target-binding affinity, internalization efficiency and the efflux of payload, as well as pharmacokinetics related to the Fc region.

#### Systemic factors at a macro level

Systemic factors in patients, such as female sex, advanced age, poor nutritional status, obesity [240], diabetes, dysbiosis of the gut microbiota [241], specific sites of metastasis (e.g., brain and liver metastases), use of antibiotics or certain targeted therapies, high tumor burden, specific infections, pain or negative emotions, and autoimmune diseases, are all associated with poor treatment response. The majority of SCLC patients are elderly, characterized not only by the redistribution and accumulation of fat in bone marrow, muscle, liver, and other ectopic sites but also by metabolic disturbances in senescent cells, including increased reactive oxygen species (ROS) production, mitochondrial dysfunction, and enhanced aerobic glycolysis. Furthermore, these senescent cells secrete a range of inflammatory cytokines and proteases into the local environment, attracting immune cells and altering the biological behavior of neighboring cells, thereby creating a microenvironment that is unfavorable for tumor suppression [222].

#### Lessons and challenges in SCLC drug resistance research

The ultimate goal of precision medicine for SCLC lies in clarifying the heterogeneity of treatment responses. First, there is an urgent need to improve in vitro and in vivo research models (such as the use of air–liquid interface culture systems, microfluidic tumor-on-a-chip devices [242]), immune cell-assembled organoids, and genetically engineered mouse models to better mimic human immune and vascular components, microbial diversity, clonal selection pressures, and other relevant biological features. The integration of AI models for analyzing high-content imaging data and metabolomic data [243], in parallel, processing the SCLC-CellMiner database and the Cancer Dependency Map that contain drug sensitivity profile information [46,237,244], has the potential to enhance the reproducibility and scalability of experiments, providing unprecedented insights into the mechanisms underlying cancer metastasis and dissemination [245] and for therapeutic strategies. Second, translating the results of mouse experiments into clinical applications requires a detailed analysis of the structure and function of various proteins, as well as the relationships and spatial organization of cells [246]. This can be achieved using tools such as π-HuB (proteomic navigator of the human body) and iterative AlphaFold [247], in combination with spatial transcriptome-wide profiling [248], RARE-seq (random priming and affinity capture of cfRNA fragments for enrichment analysis) [249], and reverse padlock amplification-based fluorescent in situ hybridization (RAEFISH), which offers whole-genome coverage and achieves single-molecule level spatial resolution [250]. Moreover, novel gene-editing technologies such as single-cell CRISPR [251] and RNA interference (RNAi) can be integrated with AI [252] to rapidly and comprehensively screen for new functional genes, and even map the fate trajectories of T cells in relation to ICI treatment responses. The novel RECODR (REsistance through COntext DRift) computational approach, which goes beyond differential coexpression analysis—such as single-cell DRUG and Beyondcell methods—by capturing dynamic changes in gene relationships over time, enables the identification of hidden drivers of drug resistance and has the potential to reshape patient stratification strategies and the design of combination therapies [253].

In summary, greater efforts powered by AI are needed in deepening our understanding of how specific chromatin modifications or accessibility, transcription factors, and oncogenic pathways are regulated in cancer cells at primary and metastatic sites—alongside their biochemical interactions with noncancerous cells, including immune cell exhaustion and biodistribution. Additionally, based on the fact that in SCLC drug resistance is characterized by redundancy, developing and implementing carefully designed combination treatments is an irrefutable strategy [154,254,255].

## Treatment of SCLC

### Pretreatment evaluation of SCLC

The WHO recognizes 2 histological subtypes of SCLC—pure and mixed types. The mixed type is generally more aggressive, with no requirement in terms of specific proportions of squamous cell carcinoma, adenocarcinoma, or spindle/polygonal cell carcinoma, whereas large cell carcinoma must constitute at least 10%. NE differentiation markers of SCLC include serotonin, GRP, neuron-specific enolase (NSE), bombesin, neurofilaments, neural cell adhesion molecule (NCAM, CD56), neuronal voltage-gated calcium channels (VGCCs), and synaptic proteins [61], as well as insulinoma-associated protein 1 (INSM1) [256], CGRP, chromogranin A, and ASCL1. In rare cases where none of the markers are expressed, and given that at least one marker is positive in approximately 10% of NSCLC, combining POU2F3 [257], the adenocarcinoma marker Napsin A, and the squamous cell carcinoma markers p40 (or p63) is necessary to distinguish poorly differentiated NSCLC from mixed-type SCLC. In the context of contrast-enhanced CT, SCLC typically presents as a large central bronchial mass with enlarged mediastinal lymph nodes. However, a solitary peripheral nodule rather than a central mass may occasionally be observed, which possibly is identified by an AI classifier. SCLC may be associated with rare paraneoplastic syndromes. Among them, the neurological symptoms include myasthenic syndrome, sensory neuropathy, and encephalomyelitis [258]—whether the underlying mechanisms overlap with those for cognitive dysfunction from SCLC treatment is unclear.

Due to its aggressive biological behavior and the significant challenges to local therapy, the 2-stage staging system originally proposed by the Veterans Administration Lung Cancer Study Group has been widely adopted for SCLC and continues to be the cornerstone for clinical decision-making [16]. The limited stage (LS) is characterized by tumor localization within the ipsilateral thorax, which can be safely encompassed within the radiation field, although the clinical boundaries may be indistinct. In contrast, the extensive stage (ES) denotes disease that extends beyond the ipsilateral thoracic cavity, including malignant pleural or pericardial effusion, and hematogenous metastases. For the majority of SCLC cases, ^18^F-fluorodeoxyglucose (FDG) positron emission tomography/computed tomography (PET/CT), as well as the novel 68Ga-satoreotide trizoxetan PET/CT [259], demonstrates superior staging accuracy compared with conventional CT imaging alone. For example, approximately 19% of patients with limited-stage disease were upstaged to extensive disease, while 8% were down-staged, prompting modifications in treatment planning, such as alterations of the radiation field [260].

The more refined tumor–node–metastasis (TNM) system (AJCC Cancer Staging Manual, 8th edition) is generally applied to patients with T1–T2, N0 disease who are candidates for surgery and/or radiation therapy [261]. Given the rapid cell proliferation and pronounced systemic symptoms of SCLC, diagnosis and staging should be performed within 1 week for treatment to be initiated as soon as possible, and this is even in the absence of comprehensive immunological features (such as PD-L1 expression) or molecular profiling results, as the clinical relevance of these markers in guiding therapeutic strategies is still unclear. Foreseeably, applying AI models with large-scale pretraining and ultra-large-context capabilities, such as Prov-GigaPath [150] or digital pathology imaging technologies [262], which can identify genomic mutation burden, methylation status, PD-L1 expression, TIL density, and the spatial relationships of immune cells within the TME [263], is expected to rapidly produce accurate and fine-grained results [264].

### Treatment of limited-stage SCLC

Only approximately 5% of LS-SCLC patients with T1–2, N0, M0 disease are considered suitable for surgical resection, despite the preoperative pathological status often being unknown. Nevertheless, most of the data supporting surgical benefits come from retrospective studies [265]. For LS-SCLC patients outside this range—such as those with T3–4, N0, M0 or T1–4, N1–3, M0 disease—chemotherapy combined with concurrent thoracic radiotherapy [266–268], or sequential radiotherapy, is generally recommended to achieve curative outcomes. In the future, AI-assisted preoperative surgical planning, intraoperative robotic real-time visual feedback, and postoperative complication risk prediction for SCLC may expand the surgical indications and improve prognosis [264]. Although surgery may still be considered after chemotherapy, patients with lymph node involvement do not appear to derive a survival benefit. Fortunately, surgical resection following immunotherapy in patients with stage I–III SCLC has yielded promising results [269]. Adjuvant chemotherapy following surgery is indicated for patients with pathologically node-negative disease (R0 resection) who have stage I–IIA (T1–2N0) SCLC [270], and may also be considered in patients undergoing surgery after induction chemotherapy. Etoposide plus cisplatin (EP) is the most commonly used regimen for LS-SCLC patients [271], resulting in a response rate of 70% to 90%, a median OS of 25 to 30 months, and a 5-year OS rate of 31% to 34%.

For LS-SCLC patients who achieve disease-free survival after concurrent chemoradiotherapy, consolidation therapy with durvalumab has been shown to provide significant benefits, as demonstrated by the ADRIATIC trial [272], which reported a median OS of 55.9 months in the durvalumab group compared to 33.4 months in the placebo group (Table 1). This benefit may also be extrapolated to patients receiving sequential systemic therapy followed by radiotherapy [273]. Prophylactic cranial irradiation (PCI) is typically recommended prior to consolidation with durvalumab (Fig. 5). Nonetheless, in the context of patients with stage I SCLC (T1–2a, N0, M0) or elderly patients, extreme prudence should guide the administration of PCI due to an increased risk of cognitive decline [274]. For patients with lymph node involvement (N^+^), or positive resection margins (R1/R2 resection), local thoracic radiotherapy combined with chemotherapy is recommended either concurrently or sequentially. However, the role of durvalumab consolidation in such patients remains unclear.

**Table 1.** Current therapies for LS-SCLC

Phases	Treatment regimens	Primary endpoints ^a^	ORR	PFS	OS	AEs (≥3 grade)	Approval times (years)	Trial names or NCT ID
II	EP	NA	86.00%	MDR = 39.00 weeks	70.00 weeks	NA	NA	NA [271]
II	EC	ORR	63.00%	NA	11.60 months	Leukopenia = 60.00%, thrombocytopenia = 51.00%	NA	NA [530]
Retrospective study	Surgery + adjuvant therapy (platinum vs. nonplatinum regimens)	NA	NA	NA	5-year OS = 68.00% vs. 32.20% (*P* = 0.04)	NA	NA	NA [531]
Retrospective study	Surgery	NA	NA	5-year DFS = 46.00%	5-year OS = 52.00%	NA	NA	NA [532]
Retrospective study	Chemotherapy + 60 Gy once-daily thoracic radiation	NA	NA	3-year PFS = 25.00%	3-year OS = 23.00%	NA	NA	NA [282]
II	Paclitaxel + topotecan → EC + 70 Gy once-daily radiotherapy	ORR	92.00%	FFS = 13.40 months	22.40 months	Dysphagia = 21.00%, febrile neutropenia = 16.00% dyspnea = 5.00%	NA	CALGB 39808 [278]
Retrospective study	Surgery + adjuvant therapy vs. surgery	OS (*P* = 0.05)	NA	NA	66.00 months vs. 42.10 months (*P* < 0.01)	NA	NA	NA [270]
II	60 Gy (once-daily) vs. 45 Gy (twice-daily) + EP/EC	2-year OS	82.10% vs. 81.60%	18.60 months vs. 10.90 months (*P* = 0.13)	2-year OS = 74.20% vs. 48.10% (*P* < 0.001), OS = 37.20 months vs. 22.60 months (*P* = 0.012)	Neutropenia = 81.00% vs. 81.00%, thrombocytopenia = 24% vs. 25%, anemia = 16% vs. 20%	NA	THORA [283]
II	65 Gy (once-daily) vs. 45 Gy (twice-daily) + EC	PFS	91.00% vs. 92.60%	17.20 months vs. 13.40 months (*P* = 0.031)	39.30 months vs. 33.60 months (*P* = 0.137)	Acute lymphopenia = 71.70% vs. 40.20% (*P* < 0.001)	NA	NCT02337712 [533]
III	Durvalumab vs. Placebo	PFS (*P* = 0.028), OS (*P* = 0.017)	NA	16.60 months vs. 9.20 months (*P* = 0.02)	55.90 months vs. 33.40 months (*P* = 0.01)	24.40% vs. 24.20%	2024/FDA	ADRIATIC [272]
II	TQB2450 + EC → surgery or RT → TQB2450	ORR	92.50%	16.20 months	NR	47.50%	NA	LungMate-005 [269]
II	Durvalumab + EP/EC → RT + durvalumab	PFS	82.40%	17.00 months	32.00 months	33.40%	NA	NCT06371482 [275]
III	SDR-RT + CT vs. CF-RT + CT	PFS	NA	16.00 months vs. 16.00 months (*P* = 0.015)	40.00 months vs. 40.00 months	54.50% vs. 66.90% (*P* = 0.020)	NA	SDR-RT trial [281]

AEs, adverse events; CCTRT, chemotherapy and concurrent thoracic radiotherapy; CF-RT, conventional fractionated radiotherapy; CT, chemotherapy; DFS, disease-free survival; EC, etoposide/carboplatin; EP, etoposide/cisplatin; FDA, Food and Drug Administration; FFS, failure-free survival; LS-SCLC, limited-stage small cell lung cancer; MDR, median duration of response; NA, not applicable; NR, not reached; ORR, objective response rate; OS, overall survival; PFS, progression-free survival; RT, radiotherapy; SDR-RT, simultaneous integrated dose reduction-radiotherapy

^a^ All results are considered statistically significant at *P* < 0.05, unless otherwise indicated.

**Fig. 5.**
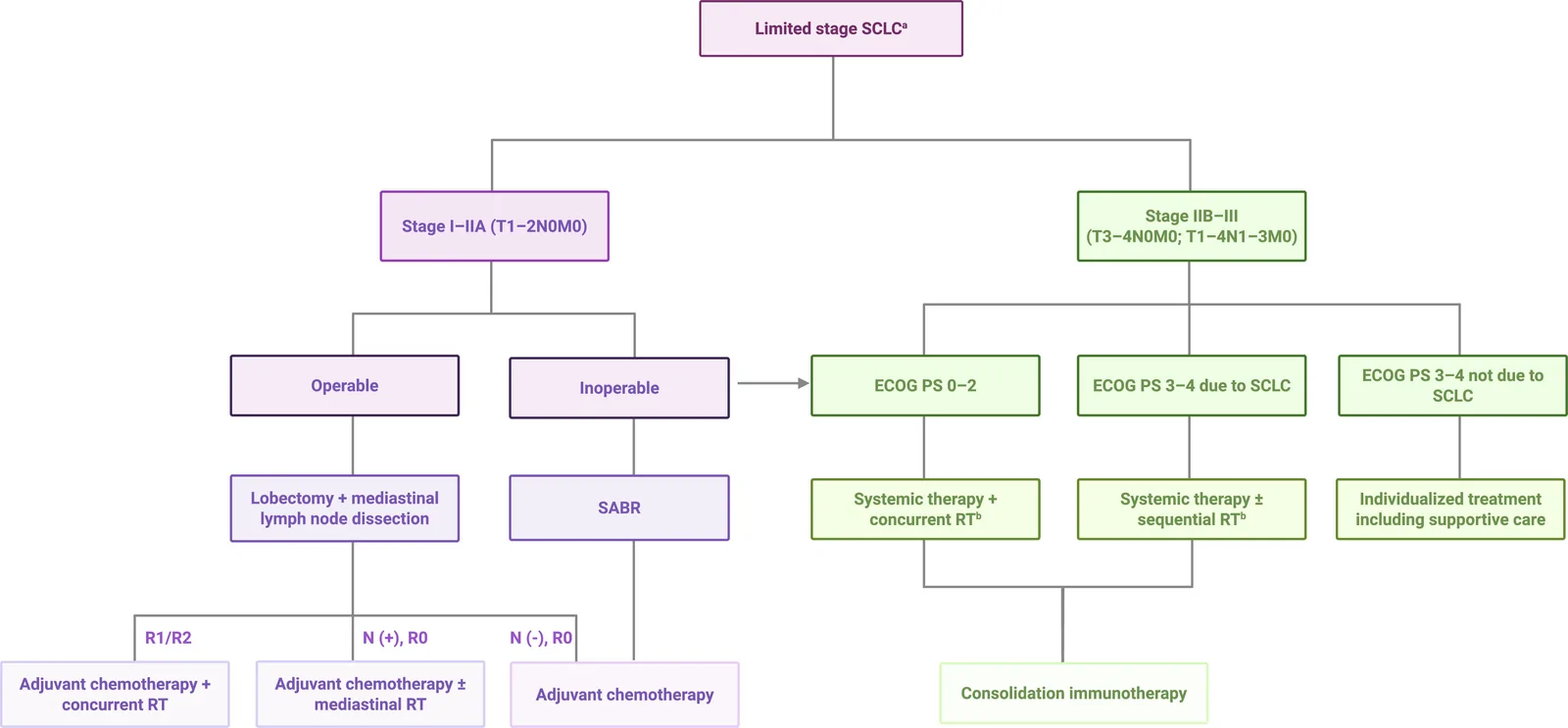
Treatment flowchart for LS-SCLC. Stage-adapted treatment strategies are recommended for limited-stage SCLC. Patients with early-stage disease (T1–2N0M0) may be considered for surgical resection or stereotactic ablative radiotherapy (SABR). For locally advanced disease, treatment selection is guided by ECOG performance status, with options including concurrent or sequential chemoradiotherapy and individualized management incorporating best supportive care. ^a^FDG PET/CT is preferred for staging when available. bConsider PCI or brain MRI surveillance after completion of concurrent or sequential chemoradiotherapy. This figure was created using BioRender. ECOG, Eastern Cooperative Oncology Group; MRI, magnetic resonance imaging; PCI, prophylactic cranial irradiation; PS, performance status; FDG PET/CT, ^18^F-fluorodeoxyglucose positron emission tomography/computed tomography; RT, radiotherapy; SABR, stereotactic ablative body radiotherapy; SCLC, small cell lung cancer.

Given the favorable outcomes of the ADRIATIC trial, we propose several questions that merit further exploration in future studies. First, whether adjuvant chemotherapy combined with immunotherapy or immunotherapy alone as a consolidation strategy should be considered in patients with SCLC following surgery. Second, whether immunotherapy can be integrated earlier into the treatment sequence, such as in combination with concurrent chemoradiotherapy—preliminary promising results have been reported [275], or whether immunotherapy in combination with chemotherapy should be followed by localized interventions such as surgery or radiotherapy. Third, given that only 30.3% of patients in the ADRIATIC trial responded to durvalumab, whether other agents or treatment strategies for advanced-stage disease—such as angiogenesis inhibitors, lurbinectedin, or tarlatamab—could be considered for enhanced consolidation therapy, despite the lack of synergistic effect with durvalumab plus tremelimumab. Fourth, given that the 60-month survival rate in the placebo group remains as high as approximately 40%, whether minimal residual disease (MRD) could be used to identify patients who may not require durvalumab consolidation therapy, representing a highly promising and intriguing area of research.

For LS-SCLC patients who are not eligible for surgery or refuse surgery, thoracic radiotherapy is recommended after 1 to 2 cycles of induction chemotherapy [276]. Encouragingly, the combination of ICIs and chemoradiotherapy has yielded promising antitumor efficacy with a favorable safety profile [277]. The radiation field should be defined based on prechemotherapy PET/CT scans to ensure coverage of the involved lymph node regions [278]. However, compared to chemotherapy alone, the addition of radiotherapy merely improves local control by 25% to 30%, and results in a 5% to 7% improvement in 2-year OS, with a 14% reduction in overall mortality [266,268]. Fortunately, the consolidation of durvalumab rather than atezolizumab [279] has significantly improved outcomes in such patients, even approaching those seen in patients with similar stages of NSCLC. Furthermore, a more intensive regimen—the addition of durvalumab to chemoradiotherapy—exhibited favorable tolerability and promising efficacy [275].

Optimally, thoracic radiotherapy [276] is recommended to be delivered in combination with early concurrent chemotherapy, rather than with late concurrent therapy, even though the former is associated with a higher incidence of severe hematologic toxicity and more frequent severe esophagitis (9% versus 4%). There is no significant difference in median OS when various delivery strategies are compared, such as low-dose fractionated radiotherapy at 45 Gy with twice-daily fractions [268,280], conventional once-daily fractionated radiotherapy with a total dose of 50 Gy [281] or 66 to 70 Gy [278,282], or hypofractionated regimens such as 42 Gy in 15 fractions. However, high-dose accelerated radiotherapy—either 65 Gy in 26 daily fractions or 60 Gy in 40 twice-daily fractions [283]—may offer survival benefits without an increase in toxicity. Larger phase III trials are still needed to validate which thoracic radiotherapy modality is superior for SCLC. Until then, the specific dose and fractionation schedule in clinical practice may need to be individualized to balance the treatment efficacy and adverse effects [284]. Given the toxicity profile, intensity-modulated radiotherapy (IMRT), 4D-CT, and PET/CT-guided techniques, as well as proton therapy, may also offer advantages over conventional approaches. Moreover, AI-driven optimal target volume delineation and dose delivery, such as Limbus Contour, as well as adaptive radiotherapy planning [264], when integrated with the above physical technologies at a fine-grained level, represent promising areas of research [285].

### Treatment of extensive-stage SCLC

#### First-line systemic therapy

Due to a high response rate (ranging from 51% to 61%) [286] and significant improvements in patient prognosis (median OS of approximately 1 year), platinum-based combination of etoposide has served as the cornerstone first-line treatment for SCLC for over 40 years, even though resistance develops within a short period. In the era of chemotherapy, other combination regimens have also been explored, but overall, they have not shown a clear advantage in terms of efficacy or toxicity compared to the EP regimen. Although initial clinical trials and meta-analyses suggested that irinotecan plus cisplatin could be superior to EP, with a median OS of 12.8 months versus 9.4 months (*P* = 0.002) and a 2-year survival rate of 19.5% versus 5.2% [287], multiple large phase III trials have since shown no significant difference in response rate or OS between the 2 regimens [288,289]. Similar results were also observed when carboplatin replaced cisplatin [290]. Given the poor bone marrow function in SCLC patients, the dose of irinotecan needs to be significantly reduced—at least 30% lower than that used in colorectal cancer. Similarly, high-dose chemotherapy or alternating non-cross-resistant regimens such as paclitaxel or cyclophosphamide [291] have shown higher complete and partial response rates in SCLC patients, but they have not improved survival [292] and are associated with significant toxicities.

In SCLC, chemotherapy induces immunogenic cell death of cancer cells, leading to the presentation or cross-presentation of tumor neoantigens to T cells, and thereby initiating an antitumor immune response [293], forming the basis for synergistic effects when combined with immunotherapy (Table 2). In 2018, a breakthrough emerged in the first-line treatment of SCLC—the Food and Drug Administration (FDA) approved atezolizumab in combination with chemotherapy, followed by atezolizumab maintenance therapy [198,294] for patients with previously untreated ES-SCLC, based on the results of the phase III IMpower133 trial. The atezolizumab-containing regimen significantly improves OS compared to chemotherapy alone, with 12.3 months versus 10.3 months (*P* = 0.0154) [198], although the response rates were similar between the 2 groups (60% versus 64%). Subsequently, the FDA approved durvalumab in combination with chemotherapy followed by durvalumab maintenance therapy as a first-line treatment for ES-SCLC, including patients with asymptomatic, untreated brain metastases [199,295]. Furthermore, in clinical trials registered in China, serplulimab (ASTRUM-005) [296], adebrelimab (CAPSTONE-1) [297], toripalimab (EXTENTORCH) [298], tislelizumab (RATIONALE-312) [299], socazolimab [300], and benmelstobart plus anlotinib [301], when used in combination with standard chemotherapy, all demonstrated improved OS and have been approved by the National Medical Products Administration (NMPA). In summary, the addition of most ICIs in treating ES-SCLC can improve OS by 1.3 to 3.0 months, with benmelstobart plus anlotinib gaining an outstanding extension of 7.4 months. The exploration of immunotherapy as monotherapy in the first-line treatment of SCLC has not been considered, mainly due to its rapid disease progression, which contrasts with the gradual activation of antitumor immune responses (Fig. 6).

**Table 2.** Current therapies for ES-SCLC or relapsed SCLC

Lines of treatment	Stages	Phases	Treatment regimens	Primary endpoints ^a^	ORR	PFS	OS	AEs (≥3 grade)	Approval times (years)	Trial names or NCT ID
First line	ES-SCLC	II	EP	NA	86.00%	MDR = 26.00 weeks	39.00 weeks	NA	NA	NA [271]
	ES-SCLC	II	EC	ORR	85.00%	NA	10.10 months	Leukopenia = 60.00%, thrombocytopenia = 51.00%	NA	NA [530]
	ES-SCLC	III	IFO + EP vs. EP	OS	73.00% vs. 67.00%	6.80 months vs. 6.00 months (*P* = 0.041)	9.10 months vs. 7.30 months (*P* = 0.045)	Anemia = 42.00% vs. 13.00%, leukopenia = 57.00% vs. 39.00%	NA	HOG LUN93-2 [534]
	ES-SCLC	III	CTX + EPI + EP vs. EP	OS	76.00% vs. 61.00% (*P* = 0.02)	7.20 months vs. 6.30 months (*P* < 0.001)	10.50 months vs. 9.30 months (*P* = 0.007)	Neutropenia = 99.00% vs. 85.00%, anemia = 51.00% vs. 18.00%, thrombocytopenia = 78.00% vs. 18.00% (*P* < 0.001)	NA	NA [535]
	ES-SCLC	III	IP vs. EP	OS	84.40% vs. 67.50% (*P* = 0.02)	6.90 months vs. 4.80 months (*P* = 0.003)	12.80 months vs. 9.40 months (*P* = 0.002)	Neutropenia = 65.30% vs. 92.20% (*P* < 0.001), leukopenia = 26.70% vs. 51.90% (*P* = 0.002), thrombocytopenia = 5.30% vs. 18.20% (*P* = 0.02), diarrhea 16.00% vs. 0% (*P* < 0.001)	NA	JCOG9511 [287]
	ES-SCLC	III	IP vs. EP	ORR	67.00% vs. 59.00% (*P* = 0.24)	9.00 months vs. 6.00 months (*P* = 0.03)	NA	Thrombopenia = 17.00% vs. 48.00% (*P* < 0.01), neutropenia = 26.00% vs. 51.00% (*P* < 0.01), diarrhea = 18.00% vs. 6.00% (*P* = 0.133)	NA	NCT00168896 [536]
	ES-SCLC	III	IC vs. EC	OS	NA	NA	8.50 months vs. 7.10 months (*P* = 0.02)	Thrombocytopenia = 15.00% vs. 26.00% (*P* = 0.05), diarrhea = 11.00% vs. 1.00% (*P* = 0.003)	NA	NA [290]
	ES-SCLC	III	Atezolizumab + EC vs. EC	PFS (*P* ≤ 0.005) and OS (*P* ≤ 0.045)	60.20% vs. 64.40%	5.20 months vs. 4.30 months (*P* = 0.02)	12.30 months vs. 10.30 months (*P* = 0.007)	58.10% vs. 57.60%	2019/FDA	IMpower133 [294]
	ES-SCLC	III	Durvalumab + EP/EC vs. EP/EC	OS (*P* ≤ 0.04)	68.00% vs. 58.00%	5.10 months vs. 5.40 months (HR 0.78)	13.00 months vs. 10.30 months (*P* = 0.005)	62.00% vs. 62.00%	2020/FDA	CASPIAN [199]
	ES-SCLC	III	Adebrelimab + EC vs. EC	OS (*P* = 0.025)	70.40% vs. 65.90%	5.80 months vs. 5.60 months (*P* < 0.001)	15.30 months vs. 12.80 months (*P* = 0.002)	86.00% vs. 85.00%	2023/NMPA	CAPSTONE-1 [297]
	ES-SCLC	III	Serplulimab + EC vs. EC	OS (*P* = 0.046)	80.20% vs. 70.40%	5.70 months vs. 4.30 months (HR = 0.48)	15.40 months vs. 10.90 months (*P* < 0.001)	82.50% vs. 80.10%	2023/NMPA 2025/EMA	ASTRUM-005 [296]
	ES-SCLC	III	Tislelizumab + EP/EC vs. EP/EC	OS (*P* = 0.021)	68.00% vs. 62.00%	4.70 months vs. 4.30 months (*P* < 0.001)	15.50 months vs. 13.50 months (*P* = 0.004)	86.00% vs. 86.00%	2024/NMPA	RATIONALE-312 [299]
	ES-SCLC	III	Toripalimab + EP vs. EP	PFS (*P* = 0.002) and OS (*P* = 0.048)	78.00% vs. 73.10%	5.80 months vs. 5.60 months (*P* < 0.001)	14.60 months vs. 13.30 months (*P* = 0.03)	89.60% vs. 89.40%	2024/NMPA	EXTENTORCH study [298]
	ES-SCLC	III	Socazolimab + EC vs. EC	OS	75.50% vs. 68.10%	5.55 months vs. 4.37 months (*P* < 0.001)	13.90 months vs. 11.58 months (*P* = 0.016)	80.30% vs. 75.70%	2025/NMPA	NCT04878016 [300]
	ES-SCLC	III	Benmelstobart + anlotinib + EC vs. anlotinib + EC vs. EC	OS and PFS	81.30% vs. 81.20% vs. 66.8% (*P* = 0.0001 and *P* = 0.0003)	6.90 months vs. 5.60 months vs. 4.20 months (*P* < 0.001)	19.30 months vs. 13.30 months vs. 11.90 months (comparative *P < 0.001* and *P* = 0.172)	93.10% vs. 94.30% vs. 87.00%	2024/NMPA	ETER701 trial [301]
	ES-SCLC	II	Atezolizumab + EP/EC + concurrent LDRT	ORR	87.50%	6.90 months	16.90 months	Neutropenia = 60.70%, leukopenia = 58.90%	NA	MATCH [325]
Consolidation/maintenance treatment	LS- SCLC and ES- SCLC	Prospective study	PCI vs. observation	Occurrence of brain metastasis	2-year occurrence rate = 40.00% vs. 67.00% (*P* <10^−13^)	NA	2-year OS = 29.00% vs. 21.50% (*P* = 0.14)	NA	NA	NA [537]
	ES-SCLC	Ib	Tarlatamab + atezolizumab/durvalumab		NA	5.60 months	25.30 months	57.00%	NA	DeLLphi-303 [310]
	ES-SCLC	III	Lurbinectedin + atezolizumab vs. atezolizumab	PFS (*P* = 0.001), OS (*P* = 0.049)	NA	5.40 months vs. 2.10 months (*P* < 0.001)	13.20 months vs. 10.60 months (*P* = 0.017)	38.00% vs. 22.00%	2024/FDA	IMforte [311]
Second-line and later-line	Relapsed SCLC	II	Gemcitabine	ORR	11.90%	NA	7.10 months	65.90%	NA	ECOG Trial 1597 [538]
	Relapsed sensitive or refractory	II	Temozolomide	ORR	23.00% (sensitive) 13.00% (refractory)	TTP 1.60 months vs. 1.00 months	6.00 months vs. 5.60 months	14.00%	NA	NCT00740636 [539]
	Relapsed SCLC	III	Topotecan + BSC vs. BSC	OS	7.00% vs. NA	TTP 16.30 weeks vs. NA	25.90 weeks vs. 13.90 weeks (*P* = 0.01)	Neutropenia = 61% vs. NA, thrombocytopenia = 38% vs. NA, anemia = 35% vs. NA	NA	NCT00276276 [338]
	Sensitive or relapsed SCLC	III	Topotecan vs. intravenous topotecan	ORR	18.30% vs. 21.90%	11.90 weeks vs. 14.60 weeks	33.00 weeks vs. 35.00 weeks (HR = 0.98)	NA	NA	NCT00003917 [339]
	Sensitive or relapsed SCLC	III	EP + irinotecan vs. topotecan	OS	84.00% vs. 27.00% (*P* < 0.001)	5.07 months vs. 3.60 months (*P* < 0.001)	18.20 months vs. 12.50 months (*P* = 0.008)	Febrile neutropenia = 31.00% vs. 7.00%, thrombocytopenia = 41.00% vs. 28.00%, SAE = 10.00% vs. 4.00%		JCOG0605 [540]
	Sensitive or relapsed SCLC	III	EC rechallenge vs. topotecan	PFS	49.00% vs. 19.00% (*P* = 0.002)	4.70 months vs. 2.70 months (*P* = 0.004)	7.50 months vs. 7.40 months (*P* = 0.94)	SAE = 37.00% vs. 43.00%	NA	NCT02738346 [328]
	Relapsed ES-SCLC	II	Lurbinectedin	ORR	35.20%	3.50 months	9.30 months	SAE = 10.00%	2020/FDA	NCT02454972 [334]
	Relapsed ES-SCLC (CTFI ≥ 180 d)	II	Lurbinectedin	ORR	60.00%	4.60 months	16.20 months	Neutropenia = 55.00%, anemia = 10.00%, thrombocytopenia = 10.00%	2020/FDA	NCT02454972 [541]
	Relapsed ES-SCLC	II	Tarlatamab,10 mg (cohort 1) or 100 mg (cohort 2)	ORR	40.00% in cohort 1, 32.00% in cohort 2	4.90 months in cohort 1, 3.90 months in cohort 2	14.30 months in cohort 1, NR in cohort 2	59.00% in cohort 1, 64.00% in cohort 2	2024/FDA	DeLLphi-301 [331]
	Relapsed ES-SCLC	III	Tarlatamab vs. chemotherapy (topotecan, lurbinectedin, or amrubicin)	OS	35.00% vs. 20.00%	4.20 months vs. 3.70 months (*P* = 0.002)	13.60 months vs. 8.30 months (*P* < 0.001)	54.00% vs. 80.00%	2024/FDA	DeLLphi-304 [332]
	Relapsed SCLC	Ib	Izalontamab brengitecan	ORR, safety/tolerability	48.10%	4.10 months	12.20 months	75.00%	NA	BL-B01D1-101 [482]
	Relapsed SCLC	II	Ifinatamab deruxtecan	ORR	48.20%	4.90 months	10.30 months	62.00%	NA	IDeate-Lung01 [479]
	Relapsed ES-SCLC	II	Sacituzumab govitecan	ORR	41.90%	4.40 months	13.60 months	74.60%	NA	TROPiCS-03 [354]
	Relapsed ES-SCLC	II	Nab-paclitaxel + simvastatin vs. nab-paclitaxel	DCR	50.00% vs. 11.10% (*P* = 0.017)	113 d vs. 62 d (*P* = 0.029)	204 d vs. 208 d (*P* = 0.504)	Leukopenia = 7.10% vs. 0%, neutropenia = 22.20% vs. 7.10%	NA	NCT04698941 [355]
	Relapsed ES-SCLC	I/II	Pembrolizumab + lurbinectedin	ORR	46.40%	4.60 months	10.50 months	71.40%	NA	LUPER[335]
	Relapsed ES-SCLC	II	Sintilimab + anlotinib + nab-paclitaxel	ORR	60.00%	6.00 months	13.40 months	12.00%	NA	ChiCTR2100049390 [353]
	Relapsed/refractory SCLC	II	Pembrolizumab + paclitaxel	ORR	23.10%	5.00 months	9.10 months	NA	NA	NCT02551432 [542]
	Relapsed ES-SCLC	II	Camrelizumab + apatinib	ORR	34.00%	3.60 months	8.40 months	72.90%	NA	PASSION [543]
	Relapsed ES-SCLC	II	Sintilimab + anlotinib	PFS	56.80%	6.10 months	12.70 months	26.00%	NA	NCT04055792 [544]

AEs, adverse events; BSC, best supportive care; CT, chemotherapy; CTFI, chemotherapy-free interval; DCR, disease control rate; EC, etoposide/carboplatin; EP, etoposide/cisplatin; ES-SCLC, extensive-stage small cell lung cancer; HFRT, hypofractionated radiation therapy; IC, irinotecan/carboplatin; IFO, ifosfamide; LS-SCLC, limited-stage small cell lung cancer; LDRT, low-dose radiation therapy; NA, not applicable; ORR, objective response rate; PCI, prophylactic cranial irradiation; PFS, progression-free survival; OS, overall survival; TTP, time to progression

^a^ All results are considered statistically significant at *P* < 0.05, unless otherwise indicated.

**Fig. 6.**
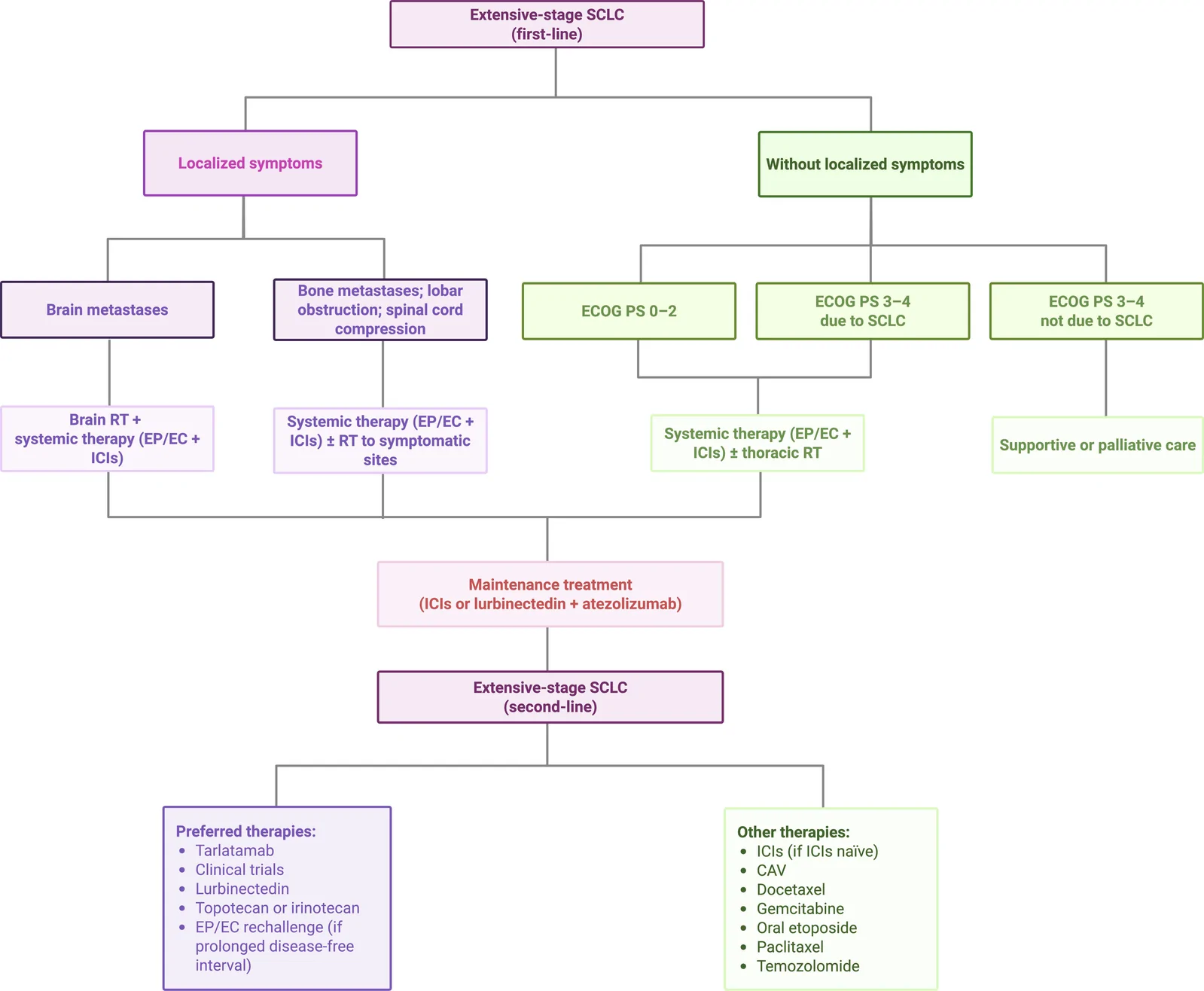
Treatment flowchart for ES-SCLC. The treatment plans for ES-SCLC are highly variable and require greater individualization and flexibility. Nevertheless, there is a lack of robust biomarkers to guide the optimization of therapeutic strategies. The upper panel presents first-line and maintenance treatment strategies for ES-SCLC, stratified by localized symptom status, whereas the lower panel summarizes subsequent-line treatment options. This figure was created using BioRender. CAV, cyclophosphamide, doxorubicin, vincristine; ECOG, Eastern Cooperative Oncology Group; EC, etoposide/carboplatin; EP, etoposide/cisplatin; ICIs, immune checkpoint inhibitors; PS, performance status; RT, radiotherapy; SCLC, small cell lung cancer.

In addition, other immunotherapy combinations with chemotherapy have been investigated, and some have shown promising results. For example, in the first-line treatment of ES-SCLC, ICIs combined with standard-dose [302] or dose-adjusted [303] chemotherapy, as well as the quadruple regimen of surufatinib plus toripalimab [304] or bevacizumab plus atezolizumab combined with chemotherapy [305], have preliminary findings of significantly prolonged OS. Nevertheless, these findings need to be confirmed by larger phase III trials.

After completing the recommended 4 to 6 cycles of chemoimmunotherapy, monotherapy with immunotherapy, rather than maintenance chemotherapy, is recommended, as the latter has not been shown to improve OS and is associated with a greater risk of cumulative toxicity [306]. In contrast, low-toxicity regimens, such as single-agent chemotherapy, or ADCs [307], anti-angiogenic agents [308], radiotherapy [309], and the latest tarlatamab [310] in combination with maintenance immunotherapy, are being further explored, with most showing promising results. Indeed, the IMforte trial, a randomized phase III study, demonstrated that patients without disease progression after 4 cycles of induction therapy (atezolizumab, carboplatin, and etoposide), and who received lurbinectedin (3.2 mg/m^2^) in combination with atezolizumab for maintenance treatment, experienced prolonged progression-free survival (PFS) and OS, both of which were superior to those in the atezolizumab monotherapy group. Despite the higher incidence of adverse events, this regimen may represent a new preferred maintenance strategy [311].

For special populations, such as patients with organ dysfunction or HIV infection, as well as those who resume treatment after recovery from toxicities, individualized chemotherapy guidance is needed in clinical decision-making [303], rather than relying solely on age or tumor burden [312–314]. For example, the subgroup analysis in the CONVERT trial showed that patients younger than 70 years and those aged 70 years or older had similar median survival times when receiving concurrent chemoradiotherapy for LS-SCLC (29.0 months versus 30.0 months, *P* = 0.38). However, close monitoring during treatment was recommended for patients aged 70 years or older to avoid excessive risk [312]. Nevertheless, setting the target area under the curve (AUC) of carboplatin at 5 instead of 6 may be more appropriate for patients aged 70 years or older.

#### Radiotherapy for extensive-stage SCLC

Radiotherapy is not the preferred initial treatment for patients with ES-SCLC; consolidative or palliative radiotherapy, however, may be considered. Limited data support the use of consolidative thoracic radiation in patients with low tumor burden and extrathoracic metastatic disease who achieve a complete or near-complete response to initial systemic therapy prior to maintenance immunotherapy [315], yet negative results from the phase II TREASURE trial [316] underscore the need for larger trials employing alternative chemoimmunotherapy regimens and radiotherapy strategies to confirm these findings. More than 50% of SCLC patients develop intracranial metastases, and PCI can effectively reduce the incidence of brain metastasis, rather than significantly prolonging survival [317]. Whether the neurologic sequelae induced by PCI [274] are associated with the interaction between NE cancer cells and CNS cells remains unclear. Therefore, in ES-SCLC, a short course or low total radiation dose of PCI (e.g., 20 Gy in 5 fractions [318], or ≤30 Gy), administered via hippocampal-avoidance intensity-modulated radiotherapy (HA-IMRT) [319], may be appropriate, although evidence continues to show that HA fails to preserve delayed memory [320]. Regarding memantine, an *N*-methyl-d-aspartate (NMDA) receptor antagonist with inconsistent results on cognitive preservation [321,322], further large-scale randomized trials are needed for evaluation.

For locally symptomatic disease, such as pain from bone metastases, spinal cord or tracheal compression, and brain metastases, local ablation and internal or external beam radiotherapy—such as IMRT, stereotactic ablative radiotherapy (SABR), or stereotactic radiosurgery (SRS)—can provide excellent palliative outcomes [323]. Moreover, continuous maintenance of prior systemic therapies is appropriate in such clinical settings [324]. In general, low-dose [325] and short-term regimens, such as 30 Gy in 10 fractions, are preferred, as these may avoid interfering with the systemic treatment for ES-SCLC, being that localized radiotherapy has only a limited impact on OS.

#### Second-line systemic treatment

Once SCLC becomes resistant to treatment, patients often face tough challenges related to rapid disease progression, a modest efficacy of second-line treatment—even with the emergence of new agents such as the DLL3-targeted BiTE, tarlatamab—and a limited number of available therapeutic options. Therefore, participation in clinical trials is often considered the primary option for SCLC patients. In general, second-line systemic treatment for SCLC depends on the duration of the interval from the end of first-line treatment to disease progression to determine sensitivity to initial treatment; this is defined by a 6-month cutoff, though occasionally a 3-month cutoff is applied [326,327]. If the interval exceeds 3 to 6 months, previously used regimens may be reutilized [328]; otherwise, non-cross-resistant agents should be considered. However, in the era of immunotherapy, whether the previous chemotherapy-free interval (CFI) definition is still appropriate to guide subsequent treatment strategies remains to be investigated. For patients currently experiencing disease progression during single-agent immunotherapy maintenance, given the limited availability of tarlatamab, continuing immunotherapy across lines, by either adding the current standard chemotherapy or combining with lurbinectedin, may be considered [329]; however, further randomized studies are needed for confirmation.

##### Tarlatamab

Tarlatamab is a bispecific T cell engaging antibody targeting DLL3, a NOTCH-inhibitory ligand that is aberrantly expressed on the surface of most SCLC cells (85% to 94%), particularly in SCLC-A and SCLC-N subtypes, with limited expression in normal cells. In phase I trials, tarlatamab demonstrated promising efficacy and acceptable safety in patients with refractory or relapsed SCLC, and updated results showed an OS of 20.3 months [330]. In the phase II DeLLphi-301 trial, patients receiving 10- and 100-mg doses of tarlatamab had PFS of 4.9 and 3.9 months, respectively, with manageable safety profiles [331]. Consequently, in May 2024, the FDA granted accelerated approval for tarlatamab as a second-line treatment for SCLC. Larger phase III studies have also confirmed that tarlatamab significantly prolonged OS compared with single-agent chemotherapy (13.6 versus 8.3 months, *P* < 0.001) [332]. These results suggest that even though SCLC is considered a “cold tumor” with low immune infiltration and T cell dysfunction [333], appropriate therapies can still overcome immune tolerance in terms of both quantity and function. Due to the limited expression of DLL3 in non-NE tumors, these tumors may be less sensitive to various DLL3-targeted therapies. However, recent stratification of the SCLC-I subtype into NE (expressing ASCL1 and/or NEUROD1) and non-NE subgroups revealed that the former exhibits a low immunosuppressive profile, suggesting that there could be potential benefit from DLL3-targeted T cell engaging molecules. In contrast, patients with high immunosuppressive features in the SCLC-I non-NE subgroup may derive greater benefit from DLL3-targeted ADCs [138]. Cytokine release syndrome (CRS), reported in 56% of patients treated with tarlatamab, was the most common treatment-related adverse event and typically occurred after the first or second dose, requiring close monitoring and prompt management [322]. In addition, other trials are evaluating tarlatamab alone in patients with limited-stage disease who have progressed after chemoradiotherapy or combined with chemotherapy and immunotherapy (including durvalumab or atezolizumab) in treatment-naïve patients with extensive-stage disease.

##### Lurbinectedin

In patients with ES-SCLC, another noteworthy second-line treatment is lurbinectedin, which binds to DNA and selectively inhibits the transcription of RNA polymerase II [334]. A phase II basket trial evaluated the efficacy of lurbinectedin (3.2 mg/m^2^ every 3 weeks) in 105 chemotherapy-experienced SCLC patients and achieved 1- and 2-year survival rates of 60.9% and 27.1%, respectively. In addition, the overall response rate was 35%, with a higher response rate of 45% observed in patients with a CFI longer than 3 months, than the 22% in those with a CFI of less than 3 months. Based on these results, the FDA granted accelerated approval for lurbinectedin for patients with SCLC who have progressed after first-line platinum-based chemotherapy. Similarly, the phase I/II LUPER study demonstrated promising efficacy of the combination of lurbinectedin plus pembrolizumab in relapsed SCLC, particularly in platinum-sensitive patients, with a known and manageable safety profile [335]. However, the phase III trial ATLANTIS found that the median OS showed no significant difference between lurbinectedin combined with doxorubicin and physician’s choice of control therapy (8.6 months versus 7.6 months, *P* = 0.70) [336]. The discrepancy may be attributed to the lower dose of lurbinectedin used in the phase III trial (2 mg/m^2^) compared to that in the previous trial [334].

##### Other treatment options

For patients with short-term (less than 3 to 6 months) recurrence, FDA and European Medicines Agency (EMA) have approved oral or intravenous topotecan as a second-line treatment [4]. Dose-fractionated administration may result in lower toxicity. Several regimens, including cyclophosphamide, doxorubicin, vincristine [337], temozolomide, paclitaxel, docetaxel, gemcitabine, irinotecan, or topotecan [338,339], can be used in patients with refractory or recurrent SCLC [327,340,341]. Overall, however, these treatments do not significantly improve survival, despite response rates ranging from 20% to 47%. Moreover, the combination of irinotecan with other agents has also failed to improve survival in patients with relapsed or refractory SCLC [342].

Several ICIs have been evaluated in patients with recurrent SCLC, but their efficacy has not been satisfactory [154,343,344]. Initially, nivolumab and pembrolizumab received FDA accelerated approval for patients who had disease progression after first-line systemic therapy, based on the encouraging response rates and durable responses observed in the CheckMate 032, KEYNOTE-158 (G cohort), and KEYNOTE-028 (C1 cohort) trials [343,345]. However, subsequent confirmatory trials—CheckMate 451 and CheckMate 331—failed to demonstrate a significant OS benefit regardless of PD-L1 expression levels [346,347], while pembrolizumab did not report confirmatory OS data within the required timeframe [348]. As a result, the FDA negotiated with the pharmaceutical companies to withdraw the second- or later-line indications for nivolumab and pembrolizumab in patients with SCLC. Similarly, the combination of durvalumab and tremelimumab, with or without stereotactic body radiotherapy (SBRT), did not demonstrate sufficient efficacy signals in relapsed SCLC [349]. However, for patients receiving third-line or later treatment, who did not previously receive immunotherapy, monotherapy with ICIs remains a reasonable and prudent option, as supported by the results of trials involving nivolumab or pembrolizumab, in which a subset of patients still showed responses (19.3%), and some even achieved complete response (CR) [345,350,351]. Additionally, considering the favorable safety profile of anti-angiogenic therapy, individualized late-line treatment may be considered, such as with anlotinib [352], particularly when combined with ICIs [353].

In addition to monotherapy with ICIs, alternative therapeutic regimens, such as single-agent sacituzumab govitecan [354] and fluoropyrimidine S-1, or simvastatin [355] incorporating cytotoxic agents, have demonstrated encouraging clinical outcomes. Moreover, the concomitant administration of sintilimab with anlotinib, alongside monotherapy with conventional chemotherapy [353], has yielded a PFS of 6 months and an OS of 13.4 months. Nevertheless, these findings require validation through large-scale, phase III randomized controlled trials. Furthermore, systemic metabolic improvements, such as dietary interventions, can enhance anticancer immune responses, which is particularly important in SCLC patients due to their poorer treatment tolerance.

In summary, our central argument emphasizes that individually tailored treatment plans should be developed through a comprehensive consideration of factors such as drug toxicity, comorbidities, patients’ economic status, and preferences, beyond the biological characteristics of the tumor itself. Moreover, patient quality of life should be given due attention, such as the management of pain or fatigue [356]; after all, not all patients prioritize a survival benefit of 1 to 2 months.

### Predictive biomarkers

Although the combination of ICIs with standard chemotherapy elicits durable responses in only 15% to 20% of patients for up to 3 years [1,198,199,357], there is an urgent need for predictive biomarkers that can identify those patients most likely to benefit from such regimens, thereby avoiding unnecessary toxicity in nonresponders [154]. In general, despite extensive research aimed at stratifying treatment strategies and predicting outcomes in SCLC based on molecular subtypes, TMB, microsatellite instability (MSI), PD-L1 expression, and methylation [358], genomic, or radiomic profiles [359], or even the fragmentomic or genomic profiles of cfDNA, no consistently effective biomarkers have yet emerged to guide clinical decision-making [360]. AI is increasingly being applied in medical prediction [361], spanning the entire spectrum of health from the molecular level—such as spatial multiomics [362]—to the cellular [363], organ system, individual, and even population or global levels. Indeed, AI models that integrate multimodal parameters, such as Lunit SCOPE IO, MANIFEST [364], the Predictive Biomarker Modeling Framework (PBMF) [365], and single-cell transcriptomics analysis and multimodal profiling (STAMP) [366], have been used to identify predictive biomarkers, enabling real-time adaptive adjustment of treatment strategies [367], which represents an inevitable trend in the future.

At the cellular level, distinct molecular subtypes of SCLC are associated with heterogeneous patterns of immunotherapy responsiveness. SCLC with NE-low phenotype, particularly the SCLC-I subtype, exhibits a heightened infiltration of cytotoxic CD8^+^ T cells, T follicular helper cells, CD8^+^CD103^+^ tissue-resident memory T cells, NK cells, antitumor macrophages [102], as well as B cells, alongside elevated expression of checkpoint molecules [343,368], which correlates with a favorable response to immunotherapy. Conversely, an increase in TAMs is linked to diminished antitumor immune activity [369], representing a prognostic factor independent of molecular subtypes [151,182]. Moreover, the presence of CD45RO^+^ memory T cells within cerebral metastases of SCLC is associated with prolonged median OS. During the evolutionary transition of SCLC toward a non-NE phenotype, epigenetic reprogramming leads to an up-regulation of MHC class I expression—a hallmark of immunogenicity [182], which represents one of the most robust predictive markers for response to ICIs [182,193].

Furthermore, within the SCLC cohort, a subset of patients with paraneoplastic neurological syndromes exhibit heightened tumor-infiltrating T cell levels, as well as prolonged PFS and OS [258,370]. However, the emergence of immune-related adverse events (irAEs), particularly those involving neurologic or psychiatric manifestations, is often a diagnostic challenge in terms of distinguishing them from preexisting or newly developed paraneoplastic symptoms [371]. In summary, although the immune microenvironment may play a pivotal role in determining immunotherapy outcomes in SCLC, current conclusions remain largely derived from retrospective analyses and are yet to be validated by prospective studies.

At the molecular levels, comprehensive genomic profiling of 324 cancer-related genes via FoundationOne analysis revealed extensive copy number alterations and chromosomal rearrangements in SCLC cells, characterized by high TMB with a median of 7.8 mutations per megabase (mut/Mb). Notably, brain metastases exhibited the highest TMB, with a median of 10.0 mut/Mb [13]. Wild-type status of TP53 or RB1 was associated with lower TMB, with the lowest values observed when both genes were wild type. However, TMB has proven to be an unreliable biomarker for predicting response to immunotherapy in SCLC. While in the CheckMate 032 trial, tumors with high TMB demonstrated greater sensitivity to nivolumab monotherapy or the combination of ipilimumab plus nivolumab [370], no significant association was observed between blood-based TMB and response to atezolizumab in the IMpower133 trial [198]. This discrepancy may be attributed to the fact that not all mutations generate high-quality or high-quantity neoantigens [343], dynamic changes in therapeutic target proteins [372], as well as the absence of a well-defined threshold for TMB. Besides, chromosomal instability (CIN) markers are associated with chemotherapy sensitivity, and conversely, tumors with stabilizing genomes or up-regulation of FOXM1 [373] tend to be resistant [374].

In SCLC, the role of PD-L1 expression, with an approximately 9.6% positivity rate [375], as a biomarker for response to ICI therapy, similarly remains controversial [376]. Results from the CheckMate 032 and IMpower133 trials [198,343] failed to demonstrate PD-L1-dependent therapeutic benefits in patients receiving nivolumab or atezolizumab. In contrast, in the KEYNOTE-028 and KEYNOTE-158 trials, higher OS was observed in patients with elevated PD-L1 expression in tumor cells or stromal cells [376]. However, there were some patients with CR who exhibited PD-L1-negative tumors, highlighting the limitations of PD-L1 as a definitive predictive marker.

In addition, dynamic and precise biomarkers derived from the TME [358], CTCs [377], or ctDNA [378,379], such as PLCG2 [103], LSD1 [182], SLFN11 [151], HMGB3 [46], B7-H3 [380], and CCNE1 amplification [13], as well as low expression of CASP10 [46], are associated with poor response to immunotherapy, including tarlatamab [377]. In contrast, tumor-resident microbiota [381] or retrovirus [382], gene amplification in the 4q12 region [13], a gene signature—consisting of CD4, MHC class II transactivator (CIITA), and TMB [383]—CCL5 [383], ERVK18 [382] and interferon-induced transmembrane protein 3 (IFITM3) expression [384], and ZFHX3 mutation [46] are correlated with improved prognosis. For patients with high SLFN11 expression, studies have shown that they exhibit heightened sensitivity to a broad spectrum of DNA-damaging agents and inhibitors, such as temozolomide, lurbinectedin [385], and etoposide, with enhanced survival outcomes observed when combined with veliparib (a PARP inhibitor)—an observation validated even in clinical trials [341]. Similarly, in chemosensitive SCLC, lurbinectedin demonstrates more pronounced efficacy as a post-chemotherapy agent, whereas tarlatamab shows the opposite effect [386]. Although these findings have been derived from subgroup or retrospective analyses, they underscore the necessity for careful stratification in the context of immunotherapy combined with chemotherapy.

### Challenges and lessons in the development of SCLC therapies

Over 4 decades, chemotherapy has persisted as the cornerstone treatment for SCLC. Even with the emergence of immunotherapy agents in recent years, their clinical benefit has largely been as additives to first-line chemotherapy [4,199,294], with only modest improvements in OS and a markedly lower proportion of long-term survivors compared to NSCLC (Table 3). Moreover, the extent to which this small but statistically significant numerical difference translates into meaningful clinical benefit requires careful evaluation, particularly in the context of economic toxicity and biological toxicity. Moreover, the development of novel drugs and therapeutic strategies for SCLC has been plagued by a striking number of failures. Multiple endeavors, such as ipilimumab or pembrolizumab combined with chemotherapy [1,387], or both tremelimumab and durvalumab added to chemotherapy [388], all failed to improve OS when compared to chemotherapy alone. Likewise, the addition of bevacizumab to chemotherapy did not yield consistently favorable OS outcomes, despite conflicting results across different studies [389,390].

**Table 3.** Clinical trials failed to be approved or showed negative results for ES-SCLC

Lines of treatment	Stages	Phases	Treatment regimens	Primary endpoints ^a^	ORR	PFS	OS	AEs (≥3 grade)	Trial names or NCT ID
First-line	LS-SCLC	Randomized controlled trial	Pulmonary resection or nonresection, both with radiotherapy to the chest and brain	OS	NA	NA	15.40 months vs. 18.60 months (*P* = 0.78)	NA	NA [545]
	LS-SCLC	III	Concurrent 66 Gy once-daily vs. 45 Gy twice-daily chemoradiotherapy	OS	NA	14.30 months vs. 15.40 months (*P* = 0.26)	25.00 months vs. 30.00 months (*P* = 0.14)	Neutropenia = 65.00% vs. 74.00%, pneumonitis = 2.00% vs. 3.00%	CONVERT [268]
	LS-SCLC	III	70 Gy once-daily vs. 45 Gy twice-daily radiotherapy	OS	84.60% vs. 83.40% (*P* = 0.767)	14.20 months vs. 13.50 months (*P* = 0.785)	30.10 months vs. 28.50 months (*P* = 0.498)	Esophageal toxicity = 17.50% vs. 16.00%	CALGB 30610/RTOG 0538 [280]
	LS-SCLC	III	EP/EC + atezolizumab + CRT vs. EP/EC + CRT	OS	59.60% vs. 60.20% (*P* = 0.40)	12.10 months vs. 11.40 months (*P* = 0.87)	31.10 months vs. 36.10 months (*P* = 0.58)	94.20% vs. 95.80%	Alliance LU005 [279]
	ES-SCLC	III	Pembrolizumab + EP/EC vs. EP/EC	PFS (*P* = 0.0048), OS (*P* = 0.0128)	70.60% vs. 61.80%	4.50 months vs. 4.30 months (*P* = 0.002)	10.80 months vs. 9.70 months (*P* = 0.016)	76.70% vs. 74.90%	KEYNOTE-604 [387]
	ES-SCLC	II	Nivolumab + EP/EC vs. EP/EC	PFS (*P* < 0.1)	77.00% vs. 80.00% (*P* = 0.44)	5.50 months vs. 4.90 months (*P* = 0.083)	11.20 months vs. 8.10 months (*P* = 0.059)	74.00% vs. 64.00%	ECOG-ACRIN EA5161 [302]
	ES-SCLC	II	Atezolizumab + CT + HFRT	Safety	72.70%	8.60 months	NA	22.50%	NCT04636762 [546]
	ES-SCLC	II	Camrelizumab + apatinib + EC	Safety	88.90%	7.30 months	17.30 months	75.00%	NCT05001412 [308]
	ES-SCLC	Ib/II	Toripalimab + surufatinib + EP	PFS > 7.30 months	97.10%	6.90 months	21.10 months	63.20%	NCT04996771 [304]
	ES-SCLC	II	Atezolizumab + bevacizumab + EC	1-year OS > 70.00%	83.30%	6.20 months	12.90 months, 1-year OS 61.80%	64.20%	CeLEBrATE [305]
	ES-SCLC	II	EP + intercalated avelumab (every 2 weeks from cycle 3)	1-year PFS > 25.00%	69.10%	5.80 months 1-year PFS 12.70%	10.30 months	56 .00%	PAVE [547]
	ES-SCLC	Ib/II	Trilaciclib + EP vs. EP	Safety	66.70% vs. 56.80% (*P* = 0.383)	6.20 months vs. 5.00 months (*P* = 0.17)	10.60 months vs. 10.90 months (*P* = 0.611)	50.00% vs. 83.80%	NCT02499770 [427]
	ES-SCLC	III	PTX + EP vs. EP	OS	75.00% vs. 68.00%	6.00 months vs. 5.90 months (*P* = 0.179)	10.60 months vs. 9.90 months (*P* = 0.169)	Grade 5 = 6.50% vs. 2.40%	CALGB 9732 [548]
	ES-SCLC	III	IP vs. EP	OS	48.00% vs. 43.60%	4.10 months vs. 4.60 months (*P* = 0.37)	9.30 months vs. 10.20 months (*P* = 0.74)	Neutropenia = 36.20% vs. 86.50% (*P* < 0.01), diarrhea = 21.30% vs. 0% (*P* < 0.01)	NCT00045162 [289]
	ES-SCLC	III	Topotecan/cisplatin vs. EP	OS (*P* = 0.05)	55.50% vs. 44.50% (*P* = 0.01)	27.40 weeks vs. 24.30 weeks (*P* = 0.01)	44.90 weeks vs. 40.90 weeks (*P* = 0.40)	Neutropenia = 35.70% vs. 35.80%, thrombocytopenia = 18.70% vs. 4.80%	NCT00320359 [549]
	ES-SCLC	III	Palifosfamide + EC vs. EC	OS (*P* = 0.05)	NA	NA	10.03 months vs. 10.37 months (*P* = 0.096)	SAE = 28.30% vs. 27.50%	MATISSE [550]
	ES-SCLC	III	Ipilimumab + EP/EC vs. EP/EC	OS (*P* = 0.05)	62.00% vs. 62.00%	4.60 months vs. 4.40 months (*P* = 0.016)	11.00 months vs. 10.90 months (*P* = 0.378)	48.00% vs. 45.00%	NCT01450761 [551]
	ES-SCLC	III	Durvalumab + tremelimumab + EP vs. durvalumab + EP vs. EP	OS (*P* = 0.04)	58.00% vs. 68.00% vs. 58.00%	4.90 months vs. 5.10 months vs. 5.40 months	10.40 months vs. 12.90 months vs. 10.50 months	73.00% vs. 65.00% vs. 65.00%	CASPIAN [295]
	ES-SCLC	II	Oblimersen + EC vs. EC	1-year OS (*P* ≤ 0.10)	61.00% vs. 60.00%	6.00 months vs. 7.60 months (*P* = 0.07)	8.60 months vs. 10.60 months (*P* = 0.02)	Grade 4 = 63.00% vs. 47.00% (*P* = 0.36)	CALGB 30103 [552]
	ES-SCLC	II	Bevacizumab (15 mg/kg) + EP/EC vs. EP/EC	PFS (HR = 0.7)	58.00% vs. 48.00% (HR = 0.31)	5.50 months vs. 4.40 months (HR = 0.53)	9.40 months vs. 10.90 months (HR = 1.16)	75.00% vs. 60.00%	SALUTE [389]
	ES-SCLC	II/III	Bevacizumab (7.5 mg/kg) + CT vs. CT	ORR at the fourth cycle	91.90% vs. 89.20% (*P* = 1.00)	5.30 months vs. 5.50 months (*P* = 0.82)	11.10 months vs. 13.30 months (*P* = 0.35)	Anemia = 8.60% vs. 16.20%	IFCT-0802 [391]
	ES-SCLC	II	Endostatin+ EC vs. EC	PFS	75.40% vs. 66.70% (*P* = 0.348)	6.40 months vs. 5.90 months (*P* = 0.213)	12.10 months vs. 12.40 months (*P* = 0.82)	Neutropenia = 55.10% vs. 39.10%	NCT00912392 [553]
Maintenance treatment	ES-SCLC	II	Sunitinib vs. placebo	PFS (*P* = 0.15)	NA	3.70 months vs. 2.10 months (*P* = 0.022)	9.00 months vs. 6.90 months (*P* = 0.16)	53.50% vs. 31.70%	CALGB 30504 (Alliance) [554]
	ES-SCLC	II	Pembrolizumab	PFS > 3.00 months	NA	1.40 months	9.60 months	NA	NCT02359019 [555]
	LS-SCLC	II	Nivolumab + ipilimumab vs. observation	PFS	38.00% vs. 47.00%	10.70 months vs. 14.50 months (*P* = 0.93)	NR vs. 32.10 months (*P* = 0.82)	62.00% vs. 25.00%	ETOP/IFCT 4-12 STIMULI [556]
	ES-SCLC	III	Nivolumab + ipilimumab vs. nivolumab vs. placebo	OS	9.10% vs. 11.50% vs. 4.10%	1.70 months vs. 1.90 months vs. 1.40 months (comparative HR = 0.72 and HR = 0.67)	9.20 months vs. 10.40 months vs. 9.60 months (comparative HR = 0.92 and HR = 0.84)	52.20% vs. 11.50% vs. 8.40%	CheckMate 451 [346]
	ES-SCLC	II	TRT (30 Gy/10 F) + atezolizumab vs. atezolizumab	OS	NA	2.40 months vs. 2.60 months (*P* = 0.85)	6.70 months vs. 13.40 months (*P* = 0.34)	61.30% vs. 18.20%	AIO-TRK-0320 [316]
	ES-SCLC	II	TRT (39 Gy) + durvalumab	12-month PFR	87.00%	6.40 months	15.00 months	23.90%	SAKK 15/19 [309]
Second-line and later-line	Relapsed ES-SCLC (>2 lines)	I/II	Pembrolizumab	ORR	19.30%	2.00 months	7.70 months	9.60%	KEYNOTE-158, KEYNOTE-028 [345] ^b^
	Recurrent SCLC	I/II	Nivolumab + ipilimumab vs. nivolumab	ORR	21.90% vs. 11.60% (*P* = 0.03)	1.50 months vs. 1.40 months	4.70 months vs. 5.70 months	37.50% vs. 12.90%	CheckMate 032 [343]
	Relapsed SCLC	II	Cabazitaxel vs. topotecan	PFS	0 vs. 10.00%	1.40 months vs. 3.00 months (*P* < 0.001)	5.20 months vs. 6.80 months (*P* = 0.013)	58.00% vs. 72.00%	NCT01500720 [557]
	Relapsed SCLC	II	Berzosertib + topotecan	ORR > 15.0 0%	5.50%	2.20 months	6.40 months	61.60%	DDRiver SCLC 250 [558]
	Relapsed/refractory SCLC	III	Dinutuximab + irinotecan vs. irinotecan vs. topotecan	OS	17.10% vs. 18.90% vs. 20.20% (*P* = 0.804)	3.50 months vs. 3.00 months vs. 3.40 months (*P* = 0.348)	6.90 months vs. 7.00 months vs. 7.40 months (*P* = 0.313)	Neutropenia = 24.00% vs. 16.60% vs. 40.90%, anemia = 7.10% vs. 9.60% vs. 35.20%	NCT03098030 [342]
	Relapsed SCLC	III	Liposomal irinotecan vs. topotecan	OS (*P* = 0.025)	44.10% vs. 21.60% (*P* < 0.001)	4.00 months vs. 3.30 months (*P* = 0.71)	7.90 months vs. 8.30 months (*P* = 0.31)	42.00% vs. 83.40%	RESILIENT [559]
	Relapsed SCLC	II	Bevacizumab + paclitaxel	PFS > 24.00 weeks	18.10%	14.70 weeks	30.00 weeks	NA	NCT00317200 [560]
	Previously platinum-treated SCLC	II	Topotecan + ziv-aflibercept vs. topotecan	3-month PFS (*P* = 0.1)	2.00% vs. 0% (*P* = 0.52)	24.00% vs. 15.00% (*P* = 0.22)	6.00 months vs. 4.60 months (*P* = 0.36)	65.00% vs. 50.00%	NCT00828139 [561]
	Relapsed/refractory SCLC	II	Paclitaxel + alisertib vs. paclitaxel + placebo	PFS	22.00% vs. 18.00%	3.32 months vs. 2.17 months (*P* = 0.113)	6.86 months vs. 5.58 months (*P* = 0.714)	76.00% vs. 51.00%	NCT02038647 [441]
	Relapsed SCLC	III	Lurbinectedin + doxorubicin vs. CAV or topotecan	OS (*P* = 0.025)	32.00% vs. 30.00%	4.00 months vs. 4.00 months (HR = 0.83)	8.60 months vs. 7.60 months (*P* = 0.90)	48.00% vs. 75.00%	ATLANTIS [336]
	Relapsed SCLC	II	Atezolizumab vs. conventional chemotherapy (topotecan or re-induction of initial chemotherapy)	ORR at 6 weeks (*P* = 0.15)	2.30% vs. 10.00%	1.40 months vs. 4.30 months (*P* = 0.004)	9.50 months vs. 8.70 months (*P* = 0.60)	NA	IFCT-1603 [562]
	Platinum-refractory/resistant ES-SCLC	II	Durvalumab + tremelimumab (arm A) vs. adavosertib + carboplatin (arm B) vs. ceralasertib + olaparib (arm C)	ORR	7.30% vs. 0% vs. 4.80% (all did not meet prespecified criteria)	1.84 months vs. 2.60 months vs. 2.92 months	5.36 months vs. 4.67 months vs. 7.56 months	19.50% vs. 60.00% vs. 28.60%	BALTIC [563]
	Relapsed SCLC	II	Durvalumab + tremelimumab vs. SBRT + durvalumab + tremelimumab	PFS, ORR	0% vs. 28.60%	2.10 months vs. 3.30 months (*P* = 0.137)	2.80 months vs. 5.70 months (*P* = 0.377)	NA	NCT02701400 [349]
	Relapsed SCLC	III	Nivolumab vs. chemotherapy (topotecan or amrubicin)	OS	13.70% vs. 16.50%	1.40 months vs. 3.80 months (HR = 1.41)	7.50 months vs. 8.40 months (*P* = 0.11)	13.80% vs. 73.20%	CheckMate 331 [347]
	Recurrent SCLC	I/II	Plinabulin + nivolumab + ipilimumab	PFS > 3.50 months	6.00%	1.60 months	5.50 months	56.00%	BTCRC-LUN17-127 [564]

AEs, adverse events; BSC, best supportive care; CAV, cyclophosphamide, doxorubicin, vincristine; CRT, chemoradiotherapy; CCTRT, chemotherapy and concurrent thoracic radiotherapy; EC, etoposide/carboplatin; ECOG, Eastern Cooperative Oncology Group; EMA, European Medicine Agency; EP, etoposide/cisplatin; ES-SCLC, extensive-stage small cell lung cancer; FDA, Food and Drug Administration; HFRT, hypofractionated radiotherapy; IP, irinotecan/cisplatin; NA, not applicable; NR, not reached; NMPA, National Medical Products Administration; ORR, objective response rate; OS, overall survival; PFR, progression-free rate; PFS, progression-free survival; SAE, serious adverse event; SBRT, stereotactic body radiotherapy; TRT, thoracic radiotherapy

^a^ All results are considered statistically significant at *P* < 0.05, unless otherwise indicated.

^b^ Strictly speaking, the results cannot be classified as negative. However, they did not include the follow-up data mandated by the FDA.

The failure of therapy in SCLC, in addition to the limited intrinsic antitumor activity of specific drugs such as pembrolizumab and bevacizumab [391] that stems from an insufficient understanding of the biological mechanisms underlying SCLC, may also be attributed to other challenges that merit further consideration, such as improper selection of endpoints for statistical analysis. First and foremost, the critical issue is the lack of effective biomarkers for patient stratification, with many clinical studies failing to enroll participants according to molecular subtypes, which could represent one of the key factors contributing to the marked variability in treatment responses. Consequently, the imperative to implement prospective clinical trials stratified by defined molecular subtypes emerges as a critical prerequisite for refining the precision of antitumoral efficacy assessment, as exemplified in randomized controlled studies targeting SLFN11-positive patient cohorts [392]. Additionally, sociodemographic factors, such as variations in economic capacity, should also be taken into account in clinical trial design, as they may contribute to regional disparities in study outcomes. The reason is that patients who discontinue from the trial and do not receive subsequent antitumoral therapy (restricted to first-line treatment) may artificially inflate the likelihood of a positive trial outcome—as in this setting, PFS is closely correlated with OS—even though such an occurrence does not compromise the validity of the results. In contrast, patients who are able to receive multiple lines of therapy, or even localized interventions, may introduce confounding effects that improve OS outcomes [393]. Geographic disparities may also be driven by the genetic background of race/ethnicity and may be confounded with the aforementioned factors, collectively impacting treatment outcomes [394].

Secondly, the majority of first-line treatments for SCLC consist of microtubule-targeting agents and DNA synthesis inhibitors, such as etoposide in combination with platinum-based chemotherapies. These modalities may share overlapping mechanisms of resistance with the payloads commonly utilized in most ADCs, such as tesirine. Therefore, the application of BiTEs with nonoverlapping resistance mechanisms or ADCs with nonconventional cytotoxic payloads represents an optimal strategy for overcoming drug resistance. Thirdly, during the drug development process, clinical trials may incorporate multiple endpoints—such as PFS and OS—or conduct multiple analyses of the study outcomes, including multiple dose groups, interim analyses at different time points, or subgroup evaluations. Reliance on a single endpoint or analysis to draw conclusions regarding drug efficacy may inevitably lead to multiplicity—a statistical issue associated with an elevated risk of type I error. To mitigate this risk, sponsors should be required, in advance, to prespecify all data analysis methodologies and statistical testing procedures that will be employed for evaluating the predefined endpoints, and to appropriately control the overall type I error rate. For example, in the dual-primary-endpoint trial of pembrolizumab combined with standard chemotherapy [387], prespecified one-sided efficacy thresholds were set at *P* = 0.0048 for PFS and *P* = 0.0128 for OS. The trial failed to meet the statistical significance threshold for OS (*P* = 0.0164), despite achieving a positive result for PFS. Therefore, to preserve adequate statistical power, strategies include increasing sample size, implementing proper α allocation, and minimizing superfluous interim analyses and treatment arms [395].

Finally, exploring strategies to decouple adverse effects from therapeutic efficacy, such as optimizing the therapeutic index or the timing and dosage of drug administration, remains a significant challenge. The recommended phase II dose (RP2D) in drug development is typically determined based on the maximum tolerated dose (MTD) identified in phase I trials, or a dose close to MTD, with the intent of achieving maximal antitumor activity. However, dose-limiting toxicities (DLTs) associated with targeted therapies often manifest at levels significantly higher than those required for clinical benefit. Moreover, MTD is typically determined based on acute toxicities, whereas certain DLTs may only become apparent after prolonged treatment [396], and the mutational signatures induced by chemotherapeutic agents in normal tissues, such as hematopoietic cells, are long-lasting. The ADC targeting DLL3, Rova-T, initially demonstrated efficacy and was highly anticipated. However, in phase II TRINITY and phase III TAHOE [397] as well as MERU [398] trials, no survival benefit was observed. This may primarily be attributed to the toxicity of ADCs, which affects nearly all patients, leading to significant dose compromises. In contrast, BiTEs are associated with milder side effects, although severe CRS may occasionally occur. Another notable finding is that the tolerability of SCLC patients is significantly reduced compared to NSCLC patients and worsens in the second-line setting. Furthermore, given that the majority of SCLC patients exhibit rapid disease progression upon clinical manifestation and are often poorly tolerant of the current 2- to 4-week screening periods during clinical trial accrual, there is an urgent need for the development of SCLC-specific screening protocols. In the future, the application of emerging technologies such as AI and digital twin methodologies to generate synthetic control groups or incorporate real-world external controls, alongside advances in digital pathology [262], has the potential to substantially reduce both the cost and duration of clinical trials [399].

Certainly, beyond drawing lessons from the aforementioned experiences, harnessing novel concepts and technologies to further expand the arsenal of SCLC-specific therapeutic strategies is imperative, as this will accordingly improve the prognosis of SCLC patients [109,154].

### Emerging therapeutic advances

Based on the extensive preclinical research advances in SCLC over the past 50 years, the primary focus of current drug development is on small-molecule compounds, antibodies (in various forms), or antibody-based therapeutics (BiTEs, ADCs, CAR-T cells, etc.), as well as vaccines, oncolytic viruses, and promising emerging modalities like proteolysis-targeting chimeras (PROTACs) (Fig. 7).

**Fig. 7.**
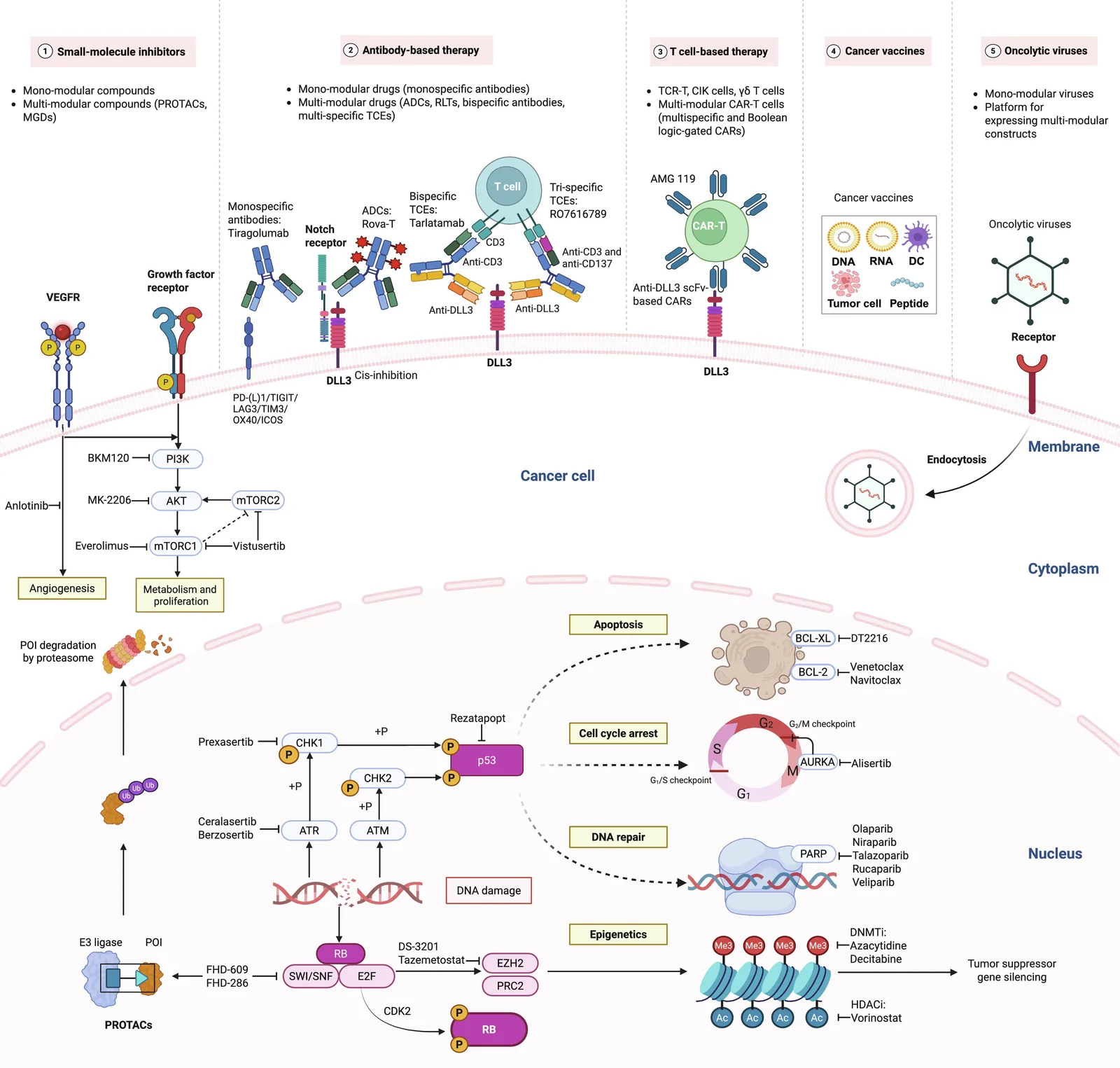
Directions for drug development in SCLC. This figure provides a comprehensive overview of nearly all drug development efforts or anticipated future breakthroughs in SCLC, although the majority are still at an early clinical or preclinical stage. This figure was created using BioRender. ADCs, antibody–drug conjugates; ATM, ataxia telangiectasia mutated; ATR, ATM and rad3-related; AURKA, aurora kinase A; BCL-2, B cell CLL/lymphoma 2; CAR-T cells, chimeric antigen receptor T cells; CDK, cyclin-dependent kinase; CHK, csk-homologous kinase; DC, dendritic cell; DLL3, delta-like ligand 3; DNMTi, DNA methyltransferase inhibitor; E3, E3 ubiquitin ligase; EZH2, enhancer of zeste homolog 2; HDACi, histone deacetylase inhibitor; ICOS, inducible costimulator; LAG-3, lymphocyte-activation gene 3; MGDs, molecular glue degraders; mTORC2, mechanistic target of rapamycin complex 2; PARP, poly (ADP-ribose) polymerase; PI3K, phosphoinositide 3-kinase; POI, protein of interest; PRC2, polycomb repressive complex 2; PROTACS, proteolysis-targeting chimeras; RB, retinoblastoma protein; RLTs, radioligand therapies; SCLC, small cell lung cancer; SWI/SNF, Switch/Sucrose Non-Fermentable; TCEs, T cell engagers; TCR-T, T cell receptor-engineered T cells; TIGIT, T cell immunoglobulin and ITIM domain; TIM3, T cell immunoglobulin and mucin domain-containing protein 3; VEGFR, vascular endothelial growth factor receptor.

#### Application of AI in drug design

Advances in AI technology [400], whether as standalone systems or integrated into AI agents, fundamentally reshape the de novo design, optimization, and repurposing of drugs. This not only improves drug efficacy and reduces side effects [400] but also compresses the discovery timeline from years to months while sustaining high hit rates and maintaining drug quality [400].

In drug discovery, high-quality training datasets serve as a critical foundation for most AI models in accurately predicting drug–target interactions [401], thereby necessitating data curation [402] before further analysis. Another key aspect is data augmentation [403] and addressing class imbalance, as well as variable scaling, encoding categorical features, and even imputing missing values [404].

##### Target identification and structure generation

Currently, various generative AI models driven by machine learning algorithms, such as diffusion or flow-based models, autoregressive models, and energy-based models, as well as generative adversarial networks (GANs) and variational autoencoders (VAEs), are capable of predicting paratope–epitope interactions and identifying cryptic pockets in novel protein targets [405], effectively guiding complex multispecific antibody or protein binder designs with markedly reduced immunogenicity, thereby accelerating the development process while minimizing trial-and-error expenses [406]. Moreover, multiple models can be integrated to enhance overall accuracy and robustness. Notably, the functionalities of various AI models inevitably overlap and synergize, and due to space limitations, this paper is unable to enumerate all models comprehensively.

The groundbreaking advancements of RoseTTAFold [407] and DeepMind’s AlphaFold system [406] signify a monumental shift from manual design to an AI-driven paradigm—a revolution honored with the 2024 Nobel Prize in Chemistry [408]. For antibody design, deep learning-based prediction and joint generation algorithms—representative tools such as AlphaFold 1 to 3 [408], tFold System (tFold-Ab and tFold-Ag) [409], AttABseq [410], and RoseTTAFold All-Atom—generate backbone scaffolds with atomic-coordinate precision and optimal geometric complementarity at the binding interface [411], not only optimizing existing antibody structures but also enabling de novo rational design, thereby outputting novel amino acid sequences that effectively stabilize the target conformation.

The strategic integration of AI in protein-ligand design also extends to small-molecule drug development. First, structure-based virtual screening (SBVS) is employed to identify high-affinity binding sites, such as cavities on protein surfaces. Second, ligand-based virtual screening (LBVS), either alone or in combination with SBVS, enables rapid screening and de novo rational design of potential candidate drug molecules [412]. Modern frameworks, such as contrastive optimization for accelerated therapeutic inference (COATI), further demonstrate exceptional multi-parameter optimization capabilities [413], generating molecules with significantly improved target selectivity and metabolic stability [414], thereby ensuring a broader therapeutic window and fewer side effects.

##### The prediction of protein-ligand binding

Leveraging learning and training from large-scale molecular datasets, AI models perform sampling (ligand sampling and protein flexibility) and scoring to rapidly predict protein-ligand docking outcomes, enabling more efficient identification of lead compounds and significantly shortening the drug development timeline while reducing costs. Representative tools include graph neural network (GNN)-based methods, such as TANKBind [415] and MILCDock, as well as graph matching networks (GMNs) and E(3)-equivariant graph neural networks (E(3)-GNNs) [416], all of which demonstrate outstanding docking prediction performance, although they may be dependent on specific databases.

Once potential drug candidates, such as lead proteins or compounds, are identified, molecular dynamics (MD) simulations become essential to analyze their dynamic physicochemical properties and noncovalent interaction networks. MD tools like AMBER [417], when synergistically employed with AI models [418], offer enhanced and more precise predictions regarding protein viscosity, aggregation, folding, and more. AI-powered modules such as Profhex [419] and Uni-QSAR, leveraging pretrained molecular representation learning models, augment the effectiveness of QSAR predictions.

##### The prediction of drug likeness

Drug likeness, or similarity to existing drugs, is a pivotal criterion in gauging a compound’s clinical translation potential. Models such as MGraphDTA [420], GLAN-DTA, Token-Mol [421], and SynLlama have advanced the assessment of drug-likeness and synthetic accessibility. Pharmacokinetics (PK) and pharmacodynamics (PD) pertain to the mechanisms by which drugs exert their effects and impact other systems in the body. Specifically designed tools, such as ADMET (absorption, distribution, metabolism, excretion, and toxicity)-AI [422] and Deep-PK tools [423], which comprehensively and efficiently assess ADMET, are pivotal in boosting therapeutic efficacy while minimizing adverse events.

##### Outlooks

AI has been extensively integrated into the discovery and optimization of novel anticancer therapeutics, including peptides [424], small proteins [425], and aptamers. However, even the most advanced computational models fall short in accurately handling membrane protein hydrophobic effects and the dynamic conformational aspects critical to affinity measurements. A promising direction for the future may be emerging computational solutions, such as cryo-electron microscopy-guided neural networks, which integrate experimental electron density maps with comprehensive sequence data. Furthermore, closed-loop active learning workflows efficaciously bridge the gap between conventional simulations and experiments, with automation exemplified by robotic crystallography systems continuously feeding real-world structural data back into neural networks for iterative model refinement, optimization, and performance enhancement [426].

#### Small-molecule compounds

Over the past 2 decades, significant progress has been made in identifying specific targets involved in cell cycle regulation, DNA damage repair, epigenetic modification, and angiogenesis. However, targeted therapy has yet to become a prominent component of the SCLC treatment landscape. Notably, in early 2021, the FDA approved trilaciclib, a cyclin-dependent kinase 4 and 6 (CDK4/6) inhibitor, for the mitigation of chemotherapy-induced myelosuppression [427].

##### Mono-modular drugs

In the context of SCLC, restoring the tumor-suppressive function of mutant p53 is a promising therapeutic strategy. Rezatapopt is a first-in-class agent that corrects the conformational defect of the p53 Y220C mutant, thereby effectively reinstating p53-mediated tumor suppression. Preclinical studies have demonstrated robust antitumor efficacy both as a monotherapy and in combination with immunotherapy. In a single case of advanced SCLC treated with rezatapopt as a later-line treatment, a durable disease regression lasting 12 weeks was observed. Currently, rezatapopt is undergoing a registration phase II clinical trial targeting patients with advanced solid tumors harboring TP53 Y220C mutation, which occurs in approximately 1% of cases [428].

Although ASCL1 and NEUROD1 are notoriously difficult to target directly, pharmacological suppression of KPNB1, BET family proteins (e.g., NHWD-870 [429], mivebresib [430], and AZD5153 [431]), the TCF3–CDK2–cyclin A2 axis (e.g., BMS265246 [432]), or USP8 can effectively reduce the levels or nuclear import of ASCL1 and NEUROD1 while enhancing their ubiquitination-dependent degradation. These inhibitory strategies induce significant antitumor effects when used as monotherapies or in combination with mTOR inhibitors or olaparib [433]. The inhibition of YES1 with CH6953755 or dasatinib elicits robust antitumor activity in organoid models as well as in cell line-derived xenograft (CDX) or PDX models.

Key mitotic molecular regulators, such as WEE1 [434], PLK1 [435], AURKA, AURKB [436], CHK1, CDK4, CDK6, and CDK7 [437,438], along with cyclin A and cyclin B RxL motifs [439], are also potential drug targets in SCLC cancer cells, although most have exhibited limited clinical progress. AURKB inhibitor barasertib (AZD1152) and AURKA inhibitors LY3295668 and DBPR278 [440] have demonstrated activity in preclinical models. The combination of the AURKA inhibitor alisertib with paclitaxel has also shown to bring modest improvement in PFS in patients with SCLC [441]. Other AURKB inhibitors, such as BI 811283 and AZD2811, are currently being evaluated in patients with solid tumors, including SCLC. In the SCLC-A subtype characterized by low MYC expression, overexpression of BCL2 family members contributes to resistance against AURKB inhibition; consequently, the supplementary targeting of BCL2 restores sensitivity to AURKB inhibition [442]. Early clinical studies have demonstrated that the exportin-1 (XPO1) inhibitor, selinexor, synergizes with such chemotherapy agents as cisplatin or irinotecan [443] with acceptable tolerability and clinical activity, and is undergoing evaluation in multiple phase II/III clinical trials (such as, NCT02419495 and NCT04256707). A hallmark of SCLC resistance is the reduced expression of BAX and the overexpression of myeloid cell leukemia 1 (MCL1). Hence, venetoclax (an FDA-approved selective BCL2 inhibitor for hematologic malignancies) combined with S63845, an MCL1 inhibitor, has displayed synergistic antitumor effects. Similarly, simultaneous targeting of BCL-XL and MCL1 using DT2216 (a BCL-XL degrader) and AZD8055 (an mTOR inhibitor) in SCLC cell lines can also play a synergistic role [444].

Once the critical regulators of DNA damage repair—such as ATM (ataxia-telangiectasia mutated) and ATR (ATM and Rad3-related protein)—are inhibited by ceralasertib and berzosertib, catastrophic mitosis and apoptosis will be induced [445]. Consequently, the combination of berzosertib with lurbinectedin [446] or topotecan has shown synergistic potential, achieving an objective response rate (ORR) of 36% in SCLC. SCLC exhibits elevated levels of PARP expression, and several studies have demonstrated that PARP inhibitors, including niraparib [447], veliparib, olaparib, rucaparib, and talazoparib, either alone or in combination with other therapies like temozolomide [341], atezolizumab [392], or even radiotherapy [448], significantly improve PFS and ORR, although no significant improvement in OS has been observed [386]. Furthermore, inhibitors of DExD/H-box helicase 9 (DHX9) and DNA-PK are currently being evaluated for their ability to induce the accumulation of DNA damage, either as monotherapy or in combination with other therapeutic modalities [449].

Another class of potential therapeutic targets [450] are epigenetic regulators or enzymes, such as KDM5A [43], LSD1/KDM1A [451], the PRC2–EZH2 complex targeting H3K4/H3K27 [193,452], ZFP36L1 [453], and HDACs [454]. However, a loss of KDM6A induces a conversion of ASCL1 to high expression of NEUROD1 [121], whereas inhibition of KDM4A reduces the levels of both ASCL1 and NEUROD1 [455]. Therefore, these findings highlight the functional differences among various epigenetic regulators, which may lead to distinct therapeutic strategies and outcomes. Moreover, in order to minimize side effects, future epigenetic drugs should be designed to target specific cell types or chromatin marks [115].

Additionally, IMPDH (inosine monophosphate dehydrogenase) inhibitors and the CDC7 inhibitor simurosertib, which modulate MYC signaling, produce stronger antitumor activity when combined with chemotherapy. Likewise, monotherapy targeting the PI3K–AKT–mTOR pathway generally yields only modest clinical gains, while the combinatorial regimen pairing the PI3K/AKT inhibitor PKI-587 with the PARP inhibitor BMN673 emerges as a promising therapeutic approach [456].

Inhibitors targeting DNA replication and DNA damage repair checkpoints, such as SLFN11 [170], NOTCH1 signaling pathway, pyroptosis pathway inducers, and chemotherapy/radiotherapy, can enhance antigen expression and presentation and DNA damage accumulation, improving the immune microenvironment [457]. Their combinations [434,458] with ICIs are also being evaluated for safety and efficacy in SCLC. Indeed, lurbinectedin has been demonstrated to activate the cyclic GMP-AMP synthase (cGAS)–STING pathway, leading to PD-L1 expression, and exhibits synergistic effects with immunotherapy [311,335]. Similarly, inhibitors targeting transcriptional and epigenetic regulatory pathways, such as PRC2 [452], EZH2, and LSD1 [459], as well as AURKA [460], are being investigated in clinical trials in combination with immunotherapy [154].

Angiogenesis has become a major focus in drug development due to its close association with the growth, metastasis, and treatment resistance of SCLC. However, apart from the 4-drug regimen of anlotinib, which has demonstrated survival benefit in patients with untreated ES-SCLC, other anti-angiogenic agents such as bevacizumab [305], axitinib, or cediranib [389] have not yielded consistent improvements in OS, regardless of their use in first-line combination therapies or maintenance treatments [461], whereas the combination of durvalumab and anlotinib or surufatinib, an angiogenic and immunomodulatory tyrosine kinase inhibitor, and toripalimab [462] holds promising clinical development potential [463].

Finally, a growing body of preclinical evidence suggests that pharmacological inhibition of Bruton tyrosine kinase (BTK), c-Kit, EGFR, IGF1R, c-MET tyrosine kinase receptor, Src family kinases [383], ARID1A, TRAF2 and NCK-interacting kinase (TNIK [161]), and adrenoceptor β2 (ADRB2) [48], along with the repurposing of hydroxychloroquine, could elicit antitumor activity in SCLC. These agents are being explored as monotherapies and in synergistic combinations with other therapeutic modalities.

##### Multi-modular drugs

PROTACs are composed of 2 distinct ligands or referred to as modules herein: one that binds to the target protein, and another that interacts with E3 ubiquitin ligases such as cereblon (CRBN) or von Hippel-Lindau (VHL), thereby initiating the degradation of the former through the ubiquitin–proteasome system. In addition, molecular glue degraders (MGDs) can induce ternary complex formation (ligase–degrader–target modules), leveraging intramolecular bivalent bridging or template-assisted covalent degradation strategies to reprogram the ubiquitin–proteasome system and facilitate the selective degradation of target proteins [464]. Multi-modular protein degradation agents possess the capacity to achieve functionalities that remain inaccessible to conventional single-modular targeted inhibitors, particularly in the degradation of otherwise undruggable proteins such as transcription factors and scaffold proteins. PROTACs exhibit a broad therapeutic target spectrum and demonstrate significant efficacy even at sub-stoichiometric doses, thereby offering enhanced potency compared to traditional small-molecule inhibitors. A growing number of degraders targeting key oncogenic drivers are currently in clinical development [464], including, but not limited to, IKAROS family zinc finger 3 (IKZF3), BCL-XL, SMARCA2, and signal transducer and activator of transcription 3 (STAT3). The degrader FHD-609, which targets BRD9—a subunit of the SWI/SNF complex—exerts antitumor activity against non-NE cells [131], whereas FHD-286, an inhibitor of SMARCA4—a catalytic subunit of the SWI/SNF complex—demonstrates efficacy against both NE and non-NE cells, and exhibits synergistic effects with the ERBB inhibitor afatinib. Similarly, ASP3082 induces the highly selective degradation of the KRAS G12D protein by forming a ternary complex comprising KRAS G12D, ASP3082, and VHL, leading to tumor regression in multiple xenograft models harboring KRAS G12D mutations. While these agents hold promise for future development as monotherapies or in combination with standard therapeutic regimens, no specific clinical trials targeting SCLC have yet been reported.

However, a significant challenge in PROTAC development lies in the fact that this advantage is less pronounced for proteins with short half-lives. Furthermore, due to their larger molecular weight compared to most conventional therapeutics, PROTACs often exhibit poor solubility, increased renal clearance, and enhanced efflux from biological compartments, thereby presenting greater bottlenecks in terms of oral bioavailability and blood–brain barrier penetration [465].

#### Antibody-based therapies

Since the FDA approved the first antibody-based drug in 1986, over 100 such therapeutics have been licensed, with many more currently under development. The first category comprises mono-modular agents, such as conventional antibodies, which directly block or activate the function of single or multiple soluble or membrane-bound molecules [466]. The second category involves multi-modular constructs, such as ADCs, radioligands (RLTs), and degrader–antibody conjugates (DACs) [467], which induce spatial proximity between the target protein and an E3 ligase, thereby facilitating tumor cell death through proteolysis. A third category includes antibodies that engage specific cell surface targets while simultaneously interacting with T cells or NK cells, thereby activating the latter to eliminate the tumor. The final category includes antibody fragments that target specific cell surface markers to generate CAR-T cells [468]. Notably, along with the evolution of therapeutic design and development, hybrid molecular architectures that transcend traditional classification boundaries have emerged. For example, the latter 2 classes can also be considered multi-modular drugs herein.

##### Mono-modular drugs

Simple pure antibody drugs, beyond PD-(L)1 inhibitors, including other target inhibitors such as lymphocyte activation gene 3 (LAG3), T cell immunoglobulin and mucin-3 (TIM3), T cell immunoglobulin and ITIM domain (TIGIT), OX40, and inducible T cell costimulator (ICOS), are currently under investigation in clinical studies for SCLC. Contradictorily, recent findings suggest that sustained ICOS costimulation limits CD8^+^ T cell responses during prolonged antigen exposure [469]. Notably, the phase III randomized trial SKYSCRAPER-02 [470] compared the standard regimen of carboplatin + etoposide combined with atezolizumab to the combination plus tiragolumab (a TIGIT inhibitor) in treatment-naïve patients, and no significant clinical benefit was observed, despite the latter regimen demonstrating acceptable tolerability. In parallel with the recognition of the pivotal role of NOTCH signaling pathway in tumor initiation and progression, a number of inhibitors targeting this pathway—such as demcizumab, tarextumab, GSI MK0752, R04929097, and PF-63084014 [44]—have been explored for therapeutic potential. Nevertheless, to date, no clinical studies reporting their application in SCLC have yet been published.

##### Multi-modular drugs

Current antibody-based drugs increasingly exhibit multifunctionality-containing different functional modules that can specifically bind to or act on 2 or more targets (such as protein domains/epitopes, lipids, or nucleic acids), or perform distinct functions (such as guidance and cytotoxic activities). This, in fact, represents a paradigm-shifting advancement, whereby mechanisms of action previously unattainable by conventional mono-modular or specific agents can be achieved by bispecific antibodies (lomvastomig [471] and bintrafusp alfa [472]), BiTEs, ADCs [473], or peptide–drug conjugates (PDCs) [474]. Certainly, the FDA has approved 70 such drugs to date, with more than half of them approved after 2020, indicating that the wave of multi-specific or multi-modular drugs is already underway [467,473].

DLL3 has garnered significant attention in the development of targeted therapeutic strategies for SCLC, including bispecific T cell engagers (TCEs), ADCs, and CAR-T cells (which will be discussed in the following section). These agents, employed either as monotherapies or in combination, are currently under extensive clinical investigation. Following the successful development of tarlatamab, a range of novel bispecific TCEs (such as obrixtamig and QLS31904 [152]), tri-specific TCEs (such as HPN328), and multi-specific TCEs that engage T cells through multiple interactions [475] have demonstrated favorable tolerability and clinical activity [476]. Recently, glycan-dependent T cell recruiters (GlyTRs) have been developed against a broad range of tumor-associated carbohydrate antigens (TACAs), demonstrating potent T cell-dependent anticancer immune responses across multiple cancer types [477]. Furthermore, diverse delivery approaches for TCEs are being explored, including localized expression through nanomaterials, oncolytic viruses, ex vivo engineering using CAR-T cells, or mRNA-based systems.

Although the phase III TAHOE trial [397] and the MERU trial [398] of Rova-T targeting DLL3 yielded negative results, other ADCs are currently under active investigation. Given the high expression of B7-H3 across various molecular subtypes of SCLC [478], preliminary clinical trials involving ifinatamab deruxtecan (I-DXd)—specifically in studies such as IDeate-PanTumor01 and IDeate-Lung01 [479]—along with HS-20093 [480], demonstrated ORR of 48.2% and 52.3%, respectively, in ES-SCLC, with a favorable safety profile. The final outcomes of the ongoing phase III trials are highly anticipated. Other DLL3-targeted ADCs, such as ZL-1310, as well as ADCs targeting a variety of different antigens (including SEZ6 [481], NCAM (CD56), and TROP2 [154], as well as EGFR-HER3 [482]), have demonstrated promising preliminary data when applied as monotherapies or in combination with other therapeutic modalities. In the future, the development of ADCs will require further optimization in several key areas, including the identification of novel target antigens, the refinement of antibody formats, the choice of intelligent linkers, and the optimization of drug loading. Moreover, in phase III trials, patient stratification based on molecular characteristics, alongside the prediction and management of adverse events, will be essential [397]. Additionally, the combination of radiopharmaceutical therapy (RPT) targeting somatostatin receptors or other targets with ADCs [483] is currently under preclinical investigation.

#### T cell-based therapies

Over the past 2 decades, significant progress has been made in tumor-targeted immunotherapy, such as CAR-T therapy for hematologic malignancies and advanced gastric cancer, and TIL therapy for melanoma [484]. Nevertheless, overall progress in solid tumors has remained largely confined to the exploratory phase of clinical investigation.

In SCLC, the high tumor-specific expression of DLL3 has emerged as a compelling target for CAR-T cell therapy [485]. Recently, in a phase I clinical trial, AMG 119, a DLL3-directed CAR-T cell product, demonstrated manageable safety and promising antitumor activity [486]. However, given the limited efficacy of CAR-T monotherapy, combination strategies with chemotherapy, radiotherapy, targeted therapy [487], and/or other immunotherapeutic agents [488] are being actively pursued to achieve synergistic therapeutic effects. For instance, combination strategies involving CAR-T therapy with the EZH2 inhibitor tazemetostat [489], or the EZH1/EZH2 inhibitor valemetostat, as well as the blockade of TIGIT and PD-1 [490], are currently being developed for SCLC and other solid tumors. Similarly, preclinical studies have shown preliminary antitumor activity of CAR-T cells targeting stemness-associated antigens (including CD133 [491], B7-H3 [492], B7H6 [493], and DLL3 [494]), either as single agents or in combination with anti-PD-1 antibodies and/or CD73 inhibitors. These findings warrant further clinical evaluation to determine their translational potential in SCLC.

The main challenges of CAR-T cell therapy include the lack of truly tumor-specific antigens or antigen loss, as well as impaired migration, infiltration, expansion, and effector function within the hostile TME. Currently, at least 50 strategies have been proposed to overcome these challenges, primarily involving the use of AI algorithms for multidimensional screening of tumor neoantigens or epitopes, and the construction of multi-modular CARs with high specificity and logical gate configurations [484] through high-throughput CRISPR screening and antibody engineering [495], or the induction of stem cell-like features to enhance the antitumor efficacy of CAR-T cells. Indeed, BCL2 mutant B7H6–CAR-T cells synergized with venetoclax [493] have demonstrated potent anti-SCLC effects both in vitro and in vivo.

Finally, CAR-γδ T, CAR-NK, and CAR-M [496] have been investigated in solid tumors to enhance therapeutic efficacy while minimizing adverse effects [497]. Currently, various delivery platforms are being explored in preclinical studies for in vivo generation of CAR-T cells, including lipid nanoparticles (LNPs), polymeric nanoparticles, bio-instructive implantable scaffolds, Cas9-packaged envelope delivery vehicles (Cas9-EDVs), virus-mimicking fusion nanovesicles (FuNVs), and lentiviral or adeno-associated viral (AAV) delivery systems. Although their application in SCLC has not yet advanced to clinical trials [498], they represent promising alternatives in the field of immunotherapy.

#### Oncolytic viruses

Oncolytic viruses (OVs) can selectively infect and lyse tumor cells by exploiting either increased sensitivity to viral sensing pathways or defective antiviral responses mediated by pattern recognition receptors, such as interferon regulatory mechanisms [499]. In SCLC, the modified vaccinia virus MYXV has demonstrated potent tumor-specific cytotoxicity while inducing immune cell infiltration. This could be the result of virus-induced immunogenic cell death, which releases damage-associated molecular patterns (DAMPs) as well as pro-inflammatory cytokines and chemokines, thereby promoting the recruitment of DCs, NK cells, and T cells to the site of infection.

Given the limited durability of responses to OVs as monotherapy, and the capacity of OVs to reprogram the immunologically “cold” TME, combining them with immunotherapeutic agents or other standard treatment modalities is an attractive strategy, regardless of whether the regimen is administered locally or systemically. Another appealing feature of OVs is their potential as a platform for expressing multi-modular constructs, such as human granulocyte-macrophage colony-stimulating factor (GM-CSF) or various cytokines (e.g., IL-12), PD-1 and PD-L1 antibodies, or BiTEs, all of which are designed to enhance robust cytotoxic effects. In addition, several studies have explored the use of OVs to express tumor-associated antigens (TAAs), which can then be combined with CAR-T cells capable of specifically recognizing these antigens. In summary, clinical trials utilizing OVs for cancer treatment have demonstrated promising efficacy in SCLC. With the emergence of ICIs and tarlatamab, combining OVs with these agents in the future to treat SCLC may have a distinctive and compelling therapeutic potential [500].

#### Tumor vaccines

Cancer vaccines function by inducing tumor-specific immune responses and immunological memory, thereby initiating the cancer-immune cycle. These vaccines can be classified based on the type of antigen they deliver, including DNA/RNA vaccines, peptide vaccines (such as whole-tumor cell lysates), or DC vaccines [501]. However, in general, the low immunogenicity of antigens, coupled with limited antigen specificity and the presence of an immunosuppressive TME, significantly hampers the antitumor efficacy of such vaccines.

Peptide-based cancer vaccines or DNA vaccines, which present or express antigens composed of 8 to 35 amino acids, also face challenges such as insufficient protein expression levels and low immunogenicity [502]. In contrast, personalized mRNA vaccines driven by genome-wide screening [503], due to their simplified and flexible manufacturing processes and the avoidance of genomic integration, are often more effective and safer [504], and thus represent a major focus of current research and development. Recently, CARVac—a vaccine containing uridine nucleoside mRNA encoding claudin 6 (CLDN6)—combined with CLDN6-specific CAR-T cells, demonstrated favorable tolerability and an ORR of 33% (7/21) [505] in relapsed or refractory solid tumors, including a case of CR.

Another strategy to enhance the efficacy of cancer vaccines is the application of appropriate delivery systems, such as nanoparticles, micelles, polymers [506], and especially hydrogels, which possess unique and superior hydrophilicity and a porous network structure that enables the free entry and exit of immune molecules and ingredients. Overall, the development of vaccines against SCLC is still nascent. Future research will continue to prioritize the identification and refinement of immunogenic antigens through AI-assisted screening approaches, multi-adjuvant modulation [507], and the rational design of costimulatory molecules.

### Challenges in SCLC drug development

The identification of highly specific molecular targets remains a critical challenge in the treatment of SCLC. Many approved drugs, or those currently under clinical research (Table S1), are developed through simple extrapolation from other cancers, such as a large number of ADCs, rather than based on highly specific targets. Moreover, drug development is frequently conducted for the same target, such as DLL3 and PD-1/PD-L1, although they have the advantages in shortening development time and lowering costs. As a result, once the resistance mechanism involves the loss of targets or a decrease in binding affinity, the entire drug series will eventually fail to be applied—that is, the prosperity of drug quantity cannot substantially improve patient prognosis, aside from the possibility of increasing drug accessibility through price competition. Therefore, given that the targets of current BiTEs and ADCs are not involved in their regulatory functions, but instead serve as specific binding sites for cell recognition, strategies combining NOTCH pathway blockade may represent a promising research direction in the future. Furthermore, the application of the AI-powered Dig model for genome-wide mapping to identify novel mutations or therapeutic targets [508], the models of ProteinMPNN and ESMFold to design novel protein antibodies [509], and to predict drug conjugation linker types, conjugation sites, and drug-to-antibody ratios, along with the interpretation of experimental data, can lead to the development of optimal solutions and significantly reduce the number of iterative cycles [510].

Secondly, the biology of SCLC exhibits significant crosstalk, primarily within the immune TME, where the functions of various cells, protein kinases, or metabolic enzymes are multifaceted and sometimes even contradictory. For example, during DC maturation, immunogenic and regulatory modules are often co-up-regulated, T cell activation and exhaustion occur simultaneously, and the functions of tumor-specific Tregs and peripheral Tregs are antagonistic in a context-dependent manner [215]. Next, although chemotherapy, ADCs, and radiotherapy for SCLC elicit significant antitumor responses, they may also lead to increased cellular plasticity and select for more aggressive subclones, even promoting metastasis [511]. Finally, the efficacy and toxicity of immunotherapy are deeply intertwined. Therefore, how to decouple or address the trade-off between these conflicting aspects—such as optimizing dosage, modulating CD3ε affinity [475], or activating adiponectin [512]—in order to reduce adverse effects without compromising therapeutic efficacy, represents a key direction for future research.

Concurrently, elucidating cancer-specific driver features and therapeutic vulnerabilities, in combination with biomaterial-based approaches, such as the targeted delivery of intelligent, programmable nanomaterials, developing more effective and less toxic targeted or immunotherapeutic agents, or investigating low-dose extratumoral irradiation (ILDR) at subtherapeutic doses (≥1 and ≤3 Gy) [513], can help expand the pharmacological arsenal. However, although a wide variety of complex nanomaterials can achieve functional diversification, only a small number progress to clinical evaluation, possibly due to overengineering, which often increases batch-to-batch variability and manufacturing complexity [506].

Thirdly, in contemporary drug development, the integration of distinct functional modules or components to construct multi-specific therapeutics may give rise to unforeseen interactions. While such interactions can, in certain instances, markedly enhance therapeutic efficacy, they may also result in diminished activity or elicit unexpected toxicities. Nevertheless, this approach, although still in its nascent phase in the context of SCLC, represents a promising frontier in the evolution of next-generation therapeutics.

## Conclusion and Perspectives

After 5 decades of stagnation, SCLC treatment has finally entered a new era defined by immunotherapy, although chemotherapy remains firmly entrenched as the cornerstone of treatment. At the same time, the rapid emergence of drug resistance and metastasis remains 2 formidable challenges in the management of SCLC.

In translational research, advancing strategies emblematic of late-line therapies further into the frontline, or even neoadjuvant and adjuvant treatment paradigms for SCLC, represents an attainable objective. Nevertheless, crucially, radioisotope-laden [514] and agent-laden ADCs that integrate therapeutic and diagnostic capabilities in real time [515], TCEs, and CAR-T [516] or CAR-NK [201], coupled with nanotechnology-enabled high-efficiency delivery systems [517], are ushering in a new era for SCLC treatment. Meanwhile, implementing multidimensional and profound blockades against pivotal targets represents another promising strategy in the context of SCLC, drawing inspiration from successes in breast cancer therapy—where the combination of antibodies and ADCs targeting the HER-2 antigen has led to significantly prolonged PFS beyond 40 months [518], effectively transitioning patients into a chronic disease state. Supplementing these efforts, the exogenous augmentation of target antigens also warrants further investigation, through which both the quality and quantity of antigens are improved, thereby addressing the dynamic plasticity [20] and heterogeneity characteristic of SCLC.

To enhance the translational efficiency of clinical research, AI agents with advanced autonomous capabilities, such as the GPT-5.4 and GPT-5.5 series [519], Google’s Gemini and Gemma, Mistral AI, and China’s DeepSeek v4 inference model, as well as “hybrid” AI platforms, are expected to enable the automation of the entire clinical research workflow [520,521]. For instance, AI agents enable autonomous generation of comprehensive patient case summaries and rapid analysis of multidimensional data encompassing clinical records, imaging, genomic information, and even cost considerations, as well as the recommendation of personalized treatment plans based on genetic markers [522,523] through accessing external information sources—including the latest research evidence, draft patient communication materials tailored to individual health literacy levels, and even aid in the identification and matching of patients to ongoing clinical trials [524]. Individual AI agents can interconnect to form “multi-agent systems”, replicating multidisciplinary tumor board consultations in clinical settings [525] (Fig. 8). While proof-of-concept studies have been published demonstrating the feasibility of their successful implementation, no AI agents have been formally integrated into routine oncological clinical practice as of yet. Therefore, to facilitate broader translational deployment of these models for SCLC, further refinements are needed to enhance their generalizability, functional robustness, and predictive output accuracy [526].

**Fig. 8.**
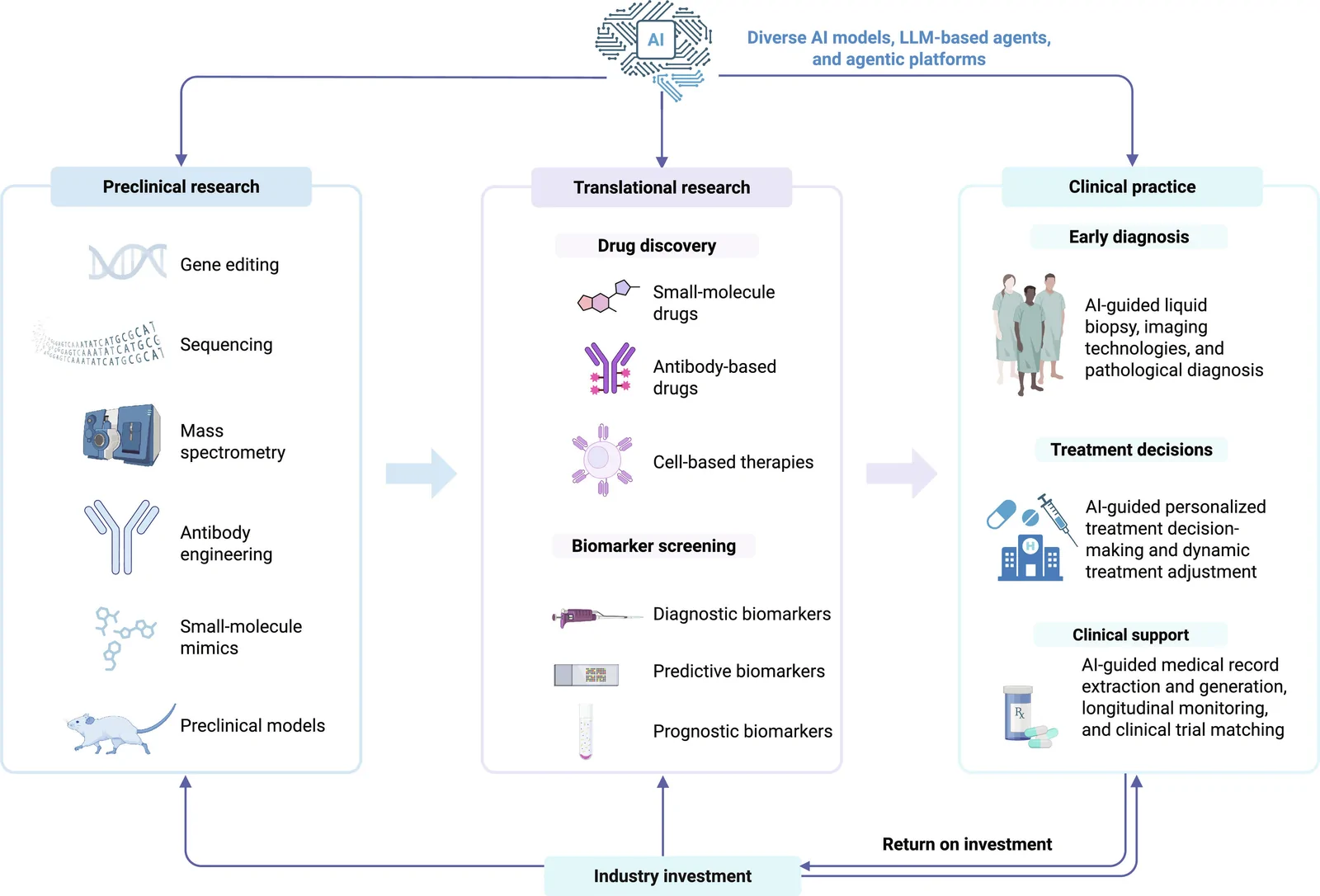
A panorama of strategies for fighting SCLC. Through multimodal embedding and generative AI, the efficacy of drugs can be evaluated, trial designs optimized, and intelligent preclinical and clinical research facilitated, thereby accelerating the drug development process. AI is employed to analyze clinical and trial data, including electronic health records, wearable devices, genomic and proteomic data, as well as imaging data. AI integrates and interprets real-world and clinical trial data, enabling clinicians to make informed adjustments to treatment strategies based on therapeutic outcomes and adverse events. This figure was created using BioRender. AI, artificial intelligence; DL, deep learning; LLM, large language model; ML, machine learning.

However, even the most advanced models, when confronted with the messy, heterogeneous data of the real world, may synthesize and analyze fabricated datasets, yielding erroneous outputs—hallucinations. Another unexpected challenge is sycophancy—the tendency to prioritize aligning with user perspectives over maintaining independent reasoning. Consequently, AI agent design principles must aim to augment, not supplant, human judgment, ensuring that ultimate oversight and accountability for decisions remain firmly in the hands of healthcare professionals. Lastly, while AI recommendations can enhance efficiency, they also heighten the risk of overreliance, potentially eroding essential professional skills and clinical judgment capabilities of practitioners over time [527]. The application of AI requires substantial computational infrastructure—whether on-premises or cloud-based—and highly skilled engineers, which may further strain already limited healthcare budgets [528]. Furthermore, the economy constitutes an integral component of the superstructure and thus shapes the pace of research progress. The poor prognosis of SCLC leads to low returns on investment for pharmaceutical companies, with only approximately 5% of research efforts dedicated to SCLC compared to NSCLC. Increased government investment in public health, such as the Recalcitrant Cancer Research Act and the 21st Century Cures Act [529] or enhanced social financing, may help break this vicious cycle.

Nonetheless, driven by the integration of data science, materials science, biosciences, and clinical sciences, optimistically, within the next decade, novel transformative drugs and therapeutic strategies may once again stir waves in the long-stagnant waters of SCLC treatment—even if the path forward proves more arduous than that of NSCLC.
