# Supplementary material for: The Five-Decade Journey of Small Cell Lung Cancer
Source: Cancer Commun (Lond). 2026 Oct 6;46:0052. doi: 10.34133/cancomm.0052 (PMC13639146; doi:10.34133/cancomm.0052)
Supplement: Supplementary file 1 [file cancomm.0052.f1.docx]

**Supplementary table S1. Clinical studies evaluation for SCLC (phases Ⅱ-Ⅲ)**

| **Drug types** | **Intervention or treatment regimens** | **Line of treatment** | **Phases** | **Primary endpoints** | **Trial names (NCT ID)** |
| --- | --- | --- | --- | --- | --- |
| Immune checkpoints | Adebrelimab + chemotherapy + synchronous LDRT vs. adebrelimab + chemotherapy | First-line | Ⅲ | PFS | NCT06610734 |
|  | Serplulimab + EC vs. atezolizumab + EC | First-line US patients | Ⅲ | OS | NCT05468489 |
|  | BNT327 + EC vs. atezolizumab + EC | First-line | Ⅲ | OS | NCT06712355 |
|  | BMS-986489 + EC vs. atezolizumab + EC | First-line | Ⅲ | OS | NCT06646276  TIGOS |
|  | Durvalumab + tremelimumab + EP vs. durvalumab + EP vs. EP | First-line | Ⅲ | OS | NCT03043872  CASPIAN |
|  | Atezolizumab + tiragolumab + EC vs. atezolizumab + EC | First-line | Ⅲ | PFS, OS | NCT04665856  SKYSCRAPER-02C |
|  | Atezolizumab + tiragolumab+ EC vs. atezolizumab + EC | First-line | Ⅲ | PFS, OS | NCT04256421  SKYSCRAPER-02 |
|  | MK-7684A + EP → MK-7684A vs. atezolizumab + EP → atezolizumab | First-line | Ⅲ | OS | NCT05224141  MK-7684A-008 KEYVIBE-008 |
|  | RT + durvalumab + EC vs. durvalumab + EC | First-line | Ⅲ | OS | NCT05223647  TRIPLEX |
|  | RT + atezolizumab or durvalumab vs. atezolizumab or durvalumab | First-line and maintenance therapy | Ⅲ | OS | NCT06719336  STONE-001 |
|  | Atezolizumab +RT vs. atezolizumab | First-line and maintenance therapy | Ⅱ/Ⅲ | PFS (Ⅱ)  OS (Ⅲ) | NCT04402788  RAPTOR Trial |
|  | Adebrelimab + RT vs. adebrelimab | First-line and maintenance therapy | Ⅲ | PFS, OS | NCT06672133  RALLY |
|  | EP → toripalimab + anlotinib | First-line and maintenance therapy | Ⅱ | PFS | NCT04363255 |
|  | PF-08634404 + chemotherapy vs. atezolizumab + chemotherapy | First-line | Ⅱ/Ⅲ | ORR, OS | NCT07226999 |
|  | lvonescimab + EP | First-line | Ⅱ | ORR | NCT07057791 |
|  | Bemotuzumab + EC/EP + anlotinib → bemotuzumab + anlotinib + RT (5Gy/5F) | First-line | Ⅱ | PFS | NCT07358676 |
|  | Tislelizumab + liposome irinotecan + platinum → liposome irinotecan + toripalimab + anlotinib | First-line and maintenance therapy | Ⅱ | PFS | NCT07376499 |
|  | Tislelizumab + EP → tislelizumab + TRT | First-line and maintenance therapy | Ⅱ | PFS | NCT07425795 |
|  | LDRT (15Gy/10F) + EP/EC + adebrelimab → adebrelimab | First-line and maintenance therapy | Ⅱ | PFS | NCT07583550 |
|  | lparomlimab + tuvonralimab + chemotherapy → TRT → lparomlimab + tuvonralimab | First-line and maintenance therapy | Ⅱ | PFS | NCT07349225 |
|  | IBI363 | At least second-line | Ⅱ | ORR | NCT07289048 |
| Chemotherapy | Lurbinectedin + atezolizumab vs. atezolizumab | First-line and maintenance therapy | Ⅲ | PFS, OS | NCT05091567  GO43104 |
|  | JK-1201I vs. topotecan | Second-line | Ⅲ | OS | NCT06581380 |
|  | Lurbinectedin + irinotecan vs. topotecan | Second-line | Ⅲ | OS | NCT06496048 |
|  | LY01610 vs. topotecan | Second-line | Ⅲ | OS | NCT06128837 |
|  | Lurbinectedin vs. lurbinectedin + irinotecan vs. topotecan or irinotecan | Second-line | Ⅲ | OS | NCT05153239  LAGOON |
| BiTEs | Tarlatamab + durvalumab + EC vs. durvalumab + EC | First-line | Ⅲ | OS | NCT07005128  DeLLphi-312 |
|  | Tarlatamab vs. SOC (topotecan, amrubicin, or lurbinectedin) | Second-line | Ⅲ | OS | NCT05740566  DeLLphi-304 |
|  | Obrixtamig + atezolizumab + EC | First-line | Ⅲ | OS | NCT07472517 |
|  | Tarlatamab | First-line | Ⅱ | PFS | NCT07423585 |
|  | ZG006 vs. SOC (topotecan or other) | Second-line | Ⅲ | OS | NCT07189455 |
| ADCs | Sacituzumab govitecan vs. SOC (topotecan or amrubicin) | Previously treated | Ⅲ | OS | NCT06801834 |
|  | I-DXd vs. TPC (topotecan, amrubicin, or lurbinectedin) | Second-line | Ⅲ | OS | NCT06203210  IDeate-Lung02 |
|  | BL-B01D1 vs. topotecan | Second-line | Ⅲ | OS | NCT06500026 |
|  | YL201 vs. topotecan | Second-line | Ⅲ | OS | NCT06612151 |
|  | HS-20093 vs. topotecan | Second-line | Ⅲ | OS | NCT06498479 |
|  | MHB088C vs. TPC (topotecan, irinotecan, or paclitaxel) | Second-line or third-line | Ⅲ | OS | NCT06954246 |
|  | GSK5764227 vs. topotecan | Second-line | Ⅲ | ORR, OS | NCT07099898 |
|  | BL-B01D1 + tislelizumab vs. tislelizumab + EC | First-line | Ⅲ | OS | NCT07502300 |
|  | ABBV-706 + atezolizumab vs. SOC (etoposide, carboplatin, atezolizumab, and optional lurbinectedin) | First-line | Ⅱ | AEs, PFS | NCT07155174 |
|  | Arm 1: lvonescimab + AK117 + EC  Arm 2: lvonescimab + cadonilimab + EC  Arm 3: lvonescimab + EC | First-line | Ⅱ | ORR | NCT07245446 |
|  | Part 1: SKB500 + KL-A167  Part 2: SKB500 + KL-A167+ carboplatin | Second-line (part 1)  First-line (part 2) | Ⅱ | AEs, PFS | NCT07296809 |
|  | ZL-1310 vs. SOC (topotecan, lurbinectedin, or amrubicin) | Second-line | Ⅲ | ORR, OS | NCT07218146 |
|  | ABBV-706 vs. SOC (topotecan, lurbinectedin, or amrubicin) | Previously treated | Ⅲ | ORR, OS | NCT07365241 |
|  | HLX43 + serplulimab | Previously treated | Ⅱ | ORR | NCT07253142 |
|  | Sacituzumab tirumotecan + anlotinib | Previously treated | Ⅱ | ORR | NCT07388875 |
| Target therapy | AL8326 vs. placebo | At least third-line | Ⅲ | OS | NCT06247605 |
|  | Chidamide + PD-L1 inhibitor + EC | First-line | Ⅱ | PFS | NCT07373964 |

Abbreviations: AEs, adverse events; EC, etoposide/Carboplatin; EP, etoposide/cisplatin; LDRT, low-dose radiation therapy; OS, overall survival; ORR, objective response rate; PFS, progression-free survival; RT, radiation therapy; SOC, standard of care; TRT, thoracic radiation therapy; TPC, treatment of physician’s choice.
